# An Optogenetic Arrhythmia Model—Insertion of Several Catecholaminergic Polymorphic Ventricular Tachycardia Mutations Into *Caenorhabditis elegans* UNC-68 Disturbs Calstabin-Mediated Stabilization of the Ryanodine Receptor Homolog

**DOI:** 10.3389/fphys.2022.691829

**Published:** 2022-03-25

**Authors:** Marcial Alexander Engel, Yves René Wörmann, Hanna Kaestner, Christina Schüler

**Affiliations:** ^1^Buchmann Institute for Molecular Life Sciences, Goethe University Frankfurt, Frankfurt, Germany; ^2^Institute of Biophysical Chemistry, Goethe University Frankfurt, Frankfurt, Germany

**Keywords:** CPVT, RyR2, calsequestrin, arrhythmia model, optogenetics, S107, Calstabin2/FKBP12.6, *C. elegans*

## Abstract

Catecholaminergic polymorphic ventricular tachycardia (CPVT) is an inherited disturbance of the heart rhythm (arrhythmia) that is induced by stress or that occurs during exercise. Most mutations that have been linked to CPVT are found in two genes, i.e., ryanodine receptor 2 (RyR2) and calsequestrin 2 (CASQ2), two proteins fundamentally involved in the regulation of intracellular Ca^2+^ in cardiac myocytes. We inserted six CPVT-causing mutations *via* clustered regularly interspaced short palindromic repeats (CRISPR)-Cas9 into *unc-68* and *csq-1*, the *Caenorhabditis elegans* homologs of RyR and CASQ, respectively. We characterized those mutations *via* video-microscopy, electrophysiology, and calcium imaging in our previously established optogenetic arrhythmia model. In this study, we additionally enabled high(er) throughput recordings of intact animals by combining optogenetic stimulation with a microfluidic chip system. Whereas only minor/no pump deficiency of the pharynx was observed at baseline, three mutations of UNC-68 (S2378L, P2460S, Q4623R; RyR2-S2246L, -P2328S, -Q4201R) reduced the ability of the organ to follow 4 Hz optogenetic stimulation. One mutation (Q4623R) was accompanied by a strong reduction of maximal pump rate. In addition, S2378L and Q4623R evoked an altered calcium handling during optogenetic stimulation. The 1,4-benzothiazepine S107, which is suggested to stabilize RyR2 channels by enhancing the binding of calstabin2, reversed the reduction of pumping ability in a mutation-specific fashion. However, this depends on the presence of FKB-2, a *C. elegans* calstabin2 homolog, indicating the involvement of calstabin2 in the disease-causing mechanisms of the respective mutations. In conclusion, we showed for three CPVT-like mutations in *C. elegans* RyR a reduced pumping ability upon light stimulation, i.e., an arrhythmia-like phenotype, that can be reversed in two cases by the benzothiazepine S107 and that depends on stabilization *via* FKB-2. The genetically amenable nematode in combination with optogenetics and high(er) throughput recordings is a promising straightforward system for the investigation of RyR mutations and the selection of mutation-specific drugs.

## Introduction

Catecholaminergic polymorphic ventricular tachycardia (CPVT) is a condition of abnormal heart rhythm (arrhythmia), induced by stress or physical activity. If untreated, CPVT is highly lethal. It often remains unnoticed due to normal baseline electrocardiograms and structurally normal hearts. The exact prevalence of CPVT is not known but is estimated to be around 1:10,000 (Liu et al., 2008). The mean age of symptom onset is between 7 and 8 years (Leenhardt et al., 1995). Most mutations that have been linked to CPVT are found in two genes, i.e., ryanodine receptor 2 (RyR2) and calsequestrin 2 (CASQ2), two proteins fundamentally involved in the regulation of intracellular Ca^2+^ in cardiac myocytes (Priori et al., 2002; Faggioni and Knollmann, 2012). The cardiac RyR2 is a Ca^2+^ release channel located in the sarcoplasmic reticulum (SR). In vertebrates, three RyR isoforms are present, whereas *Caenorhabditis elegans* has a single RyR gene encoded by the *unc-68* locus (Maryon et al., 1998). Therefore, our model may recapitulate general ryanodinopathy, even when CPVT-related RyR2 mutations are chosen. The UNC-68 protein shares about 45% sequence identity and 63% homology with the human RyR2. The expression of UNC-68 was shown by immunostaining in several muscle cell types, including pharyngeal muscles, where it was detected in the terminal bulb and posterior isthmus (Maryon et al., 1998; Hamada et al., 2002). The expression of a GFP reporter gene construct was also observed in head neurons (Maryon et al., 1998). Two EF-hand motifs and the C-terminus of UNC-68 were demonstrated to be Ca^2+^ binding regions, and based on these results, a proposed model for the functional domains of UNC-68 was found to agree well with a model of mammalian skeletal RyR (Hamada et al., 2002). Null mutants of *unc-68* are not lethal, but they move slowly and exhibit a languid, incomplete flaccid paralysis, and pharyngeal pumping is weaker than in wild type (Maryon et al., 1996). This suggests that UNC-68 is not essential for excitation-contraction coupling in nematodes but acts to amplify a calcium signal that is sufficient for contraction (Maryon et al., 1996). Calsequestrin is the major Ca^2+^ binding protein in the SR, where it forms linear chains. Only one isoform is present in *C. elegans*, CSQ-1, which shows about 31% sequence identity and 55% homology to the human CASQ2. Reporter gene analysis revealed CSQ-1 expression in body-wall muscles (BWM), vulval muscles, and in the isthmus and terminal bulb regions of the pharynx (Cho et al., 2000). A *csq-1* null-mutant showed no obvious defects in *C. elegans* muscle function or development but is highly sensitive to perturbation of Ca^2+^ homeostasis (Cho et al., 2007). Polymerization *via* back-to-back and front-to-front interaction, as well as the relevant residues, are conserved in *C. elegans* (Cho et al., 2007). While junctin and triadin are implicated in anchoring CASQ2 to RyR2 (Guo and Campbell, 1995; Jones et al., 1995), there are, based on the genome sequence, no obvious homologs of junctin and triadin in *C. elegans*. Therefore, a direct interaction of the positively charged CSQ-1 C-terminus with the negatively charged luminal loops of UNC-68 is postulated (Cho et al., 2007).

Many different (>170) CPVT mutations and polymorphisms are known (Medeiros-Domingo et al., 2009). These mutations result in a Ca^2+^ leakage from the SR, which leads to cytosolic Ca^2+^ overload, generating delayed afterdepolarizations (DADs), triggered activity, and ventricular arrhythmias, in particular, under adrenergic conditions (Leenhardt et al., 2012). There are different hypotheses about how mutations in RyR2 lead to CPVT. One possibility is that mutations in RyR2 affect the ability of calstabin2 [a.k.a. FK506-binding protein 12.6 (FKBP12.6)] to bind and stabilize RyR2 in its closed state (Wehrens et al., 2003, 2004). Recent cryo-EM of rabbit RyR2 shows that FKBP12.6 is bound to a hollow formed by the handle, Spore lysis A (SplA) kinase and Ryanodine Receptor (SPRY)1 and SPRY3 domains (Dhindwal et al., 2017). In addition, crystal structures, as well as FKBP binding and docking studies, implicate a hydrophobic cluster within a SPRY1 loop as a major FKBP binding determinant (Yuchi et al., 2015). Those areas are widely conserved in *unc-68*. A second hypothesis suggests instability *via* defective interdomain interactions, evoking so-called “unzipping” of the domains, which results in a calcium leak (George et al., 2006). However, either in combination with one of the proposed mechanisms or on its own, a sensitization of RyR2 channels to SR luminal calcium, leading to store overload-induced calcium release (SOICR), is also discussed (Jiang et al., 2004).

In this study, we aimed to investigate the effects of CPVT-related mutations on pharyngeal pumping after insertion of known mutations *via* clustered regularly interspaced short palindromic repeats (CRISPR)-Cas9 into the *C. elegans csq-1* and *unc-68* loci. Using the *C. elegans* pharynx, a rhythmically active muscular pump acting as the feeding organ of the nematode, enabled us to study their effects on contraction and intracellular Ca^2+^ concentration in an entire organ of a living animal. Obvious similarities exist between the heart and the pharynx, i.e., both are tubes that pump materials along their lumina, both possess gap junctions to synchronize their contractions that can continue without neuronal input and the fact that the pharynx expresses orthologs of most of the proteins involved in human cardiac muscle physiology. These similarities suggest that the pharynx and the myocardium may represent convergent evolution between two muscular pumps faced with similar biological roles (Mango, 2007). The pharynx itself consists of six sections which are, from anterior to posterior, the buccal cavity, procorpus, metacorpus, the isthmus, the terminal bulb, and the pharyngeal-intestinal valve (Albertson and Thomson, 1976; Mango, 2007). *C. elegans* is a filter feeder, a complex sequence of contractions and relaxations transports food particles in two successive trap stages before passage into the terminal bulb and the intestine (Fang-Yen et al., 2009). The grinder, located in the terminal bulb, crushes bacteria before passage to the intestine. The pump activity is maintained by action potentials, which qualitatively bear a resemblance to vertebrate cardiac action potentials (Franks et al., 2002). *C. elegans* lacks a voltage-gated sodium channel that initiates the action potential in the heart. In the pharyngeal muscle instead, its role is played by CCA-1, a T-type calcium channel (Shtonda and Avery, 2005). EGL-19, the *C. elegans* L-type voltage-dependent Ca^2+^channel α1-subunit, maintains depolarization during the plateau phase (∼200 ms) and activates muscle contraction. EGL-19 slowly inactivates, and EXP-2, a voltage-gated K^+^-channel, causes membrane repolarization and action potential termination (Shtonda and Avery, 2005). Though homology is low, EXP-2 is functionally similar to the human ether-a-go-go-related gene (hERG) channel (Davis et al., 1999).

We previously established the pharynx as an optogenetically controlled arrhythmia test system (Schuler et al., 2015; Fischer et al., 2017). Since spontaneous pharynx pumping is too irregular to allow the detection of arrhythmic events, we optically paced the pharynx using channelrhodopsin-2 (ChR2) to yield millisecond-precise and stable rhythmicity (Schuler et al., 2015). Blue light stimulation of pumping was achieved with a gain-of-function variant of ChR2, ChR2(H134R), that was directed to the plasma membrane of the pharyngeal muscle cells (PMCs) by the specific promoter *pmyo-2*. Importantly, we were able to show previously that the optogenetically paced pharynx, in contrast to spontaneous pumping on food, is not appreciably affected by neuronal input (Schuler et al., 2015). Furthermore, we were able to demonstrate the conservation of function between human RyR and *C. elegans* UNC-68. Incubation with caffeine (1 mM), a well-known activator of RyR, increased the spontaneous pump rate in EPG recordings of wild-type animals, while no effect of caffeine was observed in two different mutants lacking UNC-68 (Fischer et al., 2017). A deletion of *unc-68* (allele *r1162*) led to severe effects on optogenetically 4 Hz paced pumping in our pharynx model and was reversed by an expression of wild type UNC-68 in the deletion background (Fischer et al., 2017). In addition, UNC-68 was recently established to study human myopathic mutations of the skeletal muscle channel RyR1 (Graham et al., 2020). The conservation of functionality between RyR1 and UNC-68 is emphasized by an increased sensitivity to the inhalational anesthetic halothane of all tested RyR1 equivalent variants and an increased caffeine sensitivity for variants corresponding to malignant hyperthermia and central core disease (Nicoll Baines et al., 2017; Graham et al., 2020).

*Caenorhabditis elegans* is highly suited to study the effects of disease-causing mutations, not only because it is a genetically amenable experimental system but also because it enables, straightforward mass cultivation, with a 3-day life cycle. Therefore, the effects of mutations that cause human diseases can be investigated in *C. elegans* to the point that high-throughput screening becomes possible, which cannot be easily achieved in other *in vivo* models. Certainly, there are limitations: the small nematode is due to the lack of a heart and a vascular system not suitable for studying several aspects of human cardiac function and diseases including chamber development, fibrosis, hypertrophy, remodeling, and hemodynamics (Benian and Epstein, 2011). Further limitations of the pharynx regarding the analysis of CPVT mutations are that no adrenergic receptor is expressed in its muscle cells (Sanyal et al., 2004) and that the possible involvement of regulatory mechanisms by NCX or SERCA in the optogenetic arrhythmia model is not yet investigated.

We have chosen one *csq-1* mutation and five *unc-68* mutations. All chosen CPVT mutations occur in homologous regions of the respective proteins (see RyR alignment: Supplementary Information) and have been found in human patients (Table 1). Furthermore, their mechanism has been described by other groups in animal models or cell cultures (Table 1). In the case of CSQ-1, we selected a mutation bearing a putative mechanism other than triadin/junction interaction. For RyR2 mutations, we selected mutations with different putative mechanisms. In addition, the selected RyR2-mutations are distributed over the three hotspot regions known to bear CPVT-mutations: the N-terminal (aa 62–466), the central (aa 2113–2534), and the C-terminal region (aa 3778–4959), including the channel domain (Leenhardt et al., 2012). Many proteins, for example, protein kinase A or calmodulin, are associated with the N-terminal region of RyR2, whereas calsequestrin, junctin, and triadin are linked to the C-terminus (see also Leenhardt et al., 2012). We inserted the mutations into *C. elegans via* different CRISPR-Cas9 approaches (Ward, 2015; Prior et al., 2017) and crossed them with our strain expressing ChR2 in PMCs. In contrast to extrachromosomal arrays, genome-editing should most closely resemble an expression level and protein distribution reflecting the wild-type condition.

**TABLE 1:** Catecholaminergic polymorphic ventricular tachycardia (CPVT) mutations found in human characterized in this study with their homologous sites in *Caenorhabditis elegans unc-68* and their suggested mechanism.

Human	*C. elegans*	Suggested mechanism
RyR2-R420Q (Medeiros-Domingo et al., 2009; van der Werf et al., 2011; Arad et al., 2012)	*unc-68(R414Q)*	Reorients the first two N-terminal domains relative to the third domain, destabilizing intersubunit interactions (Kimlicka et al., 2013)
RyR2-S2246L (Priori et al., 2001; Tester et al., 2004; Medeiros-Domingo et al., 2009)	*unc-68(S2378L)*	Abnormally tight local sub-domain interaction, defective interaction between N-terminal and central domains (Suetomi et al., 2011) Decreased affinity for calstabin2 (Wehrens et al., 2003)
RyR2-P2328S (Lehnart et al., 2004; Medeiros-Domingo et al., 2009)	*unc-68(P2460S)*	Decreased binding of calstabin2 (Lehnart et al., 2004)
RyR2-Q4201R (Lehnart et al., 2004; Medeiros-Domingo et al., 2009)	*unc-68(Q4623R)*	Decreased binding of calstabin2 (Lehnart et al., 2004)
RyR2-I4867M (Priori et al., 2002)	*unc-68(I5231M)*	Increases channel sensitivity to activation by luminal Ca^2+^ (Jiang et al., 2005)
CASQ2-K206N (Kirchhefer et al., 2010)	*csq-1(K214N)*	Hyperglycosylation, altered cellular calcium handling (Kirchhefer et al., 2010)

We recorded conventional electropharyngeograms (EPGs), extracellular measurements of electrical events in the pharynx. The excitation phase consists of two positive peaks, the first one smaller than the second, that correlate with muscle contraction. The relaxation phase consists of two negative peaks, the second one smaller than the first, that correlate with muscle relaxation. A plateau phase is between the two phases (Raizen and Avery, 1994). In addition, we have established EPG recordings of intact nematodes with optogenetic stimulation by combining an Arduino-controlled high-power LED with a microfluidic system (ScreenChip™, *InVivo* Biosystems) that enables higher throughput than conventional electrophysiological approaches. This method allows a precise light stimulation during recordings while registering pharyngeal potentials, similar to an electrocardiogram. Several mutations of UNC-68 led to a reduced ability to follow light stimulation. This effect was reversed in a mutation-specific way by the 1,4-benzothiazepine S107, which is suggested to stabilize RyR channels by enhancing the binding of calstabin (Bellinger et al., 2008; Lehnart et al., 2008; Kushnir et al., 2020). In addition, we explored the effects of the CPVT mutations on intracellular Ca^2+^ concentration *in vivo* by using RCaMP1h, a genetically encoded Ca^2+^ indicator in the pharynx. The red-shifted RCaMP1h can be used in combination with ChR2 under optogenetic stimulation (Akerboom et al., 2013).

Since no homologs of triadin and junctin are present in *C. elegans*, we were also interested in the mode of interaction between CSQ-1 and UNC-68. This is especially because mutations of triadin are as well known to cause CPVT (Roux-Buisson et al., 2012; Rooryck et al., 2015). Thus, we disturbed the proposed electrostatic interaction by a replacement *via* CRISPR-Cas9 of negatively charged amino acids of a UNC-68 luminal loop with neutral ones.

In conclusion, we showed for several of the inserted CPVT mutations a reduction in the ability of pharyngeal pumping under optogenetic pacing and an altered Ca^2+^ handling, which can be designated here as an arrhythmia-like phenotype. The benzothiazepine S107 reversed the pumping ability of those strains in a mutation-specific manner and therefore we suggest the involvement of a FKB-2 dependent stabilization mechanism.

## Materials and Methods

### *Caenorhabditis elegans* Cultivation and Transgenic Strains

Worms were cultivated on nematode growth medium (NGM) plates (55 mm, 8 ml NGM), seeded with an *E. coli* OP50-1 strain. We used the following strains: N2 (wild type), **ZX1423:** *zxEx795 [pmyo-2::RCaMP1h; pmyo-3::CFP]*, **ZX1662:** *zxIs20 [pmyo-2::ChR2(H134R)::mCherry; pges-1::nls::GFP]*, **GE24:** *pha-1(e2123) III*, and **RB2222:** *fkb-2(ok3007) I*. Strains generated by SunyBiotech, China (SunyBiotech nomenclature in brackets): **ZX2655:** *csq-1(zx7[K214N])* [**PHX2058:** *csq-1(syb2058)*], **ZX2745:** *unc-68(zx9[neutral luminal loop])* [**PHX2420:** *unc-68(syb2420)*], **ZX2747:** *unc-68(zx10[I5231M])* [**PHX2421:** *unc-68(syb2421*)], and **ZX2749:** *unc-68(zx11[S2378L])* [**PHX2494:** *unc-68(syb2494)*].

We generated the following strains: **ZX2255:** *fkb-2(ok3007) I; zxIs20 [pmyo-2::ChR2(H134R)::mCherry; pges-1::nls::GFP]*, **ZX2256:** *pha-1(zx2); unc-68(zx4[Q4623R])*, **ZX2257:** *pha-1(zx2); unc-68(zx4[Q4623R]); zxIs20 [pmyo2::ChR2(H134R)::mCherry; pges1::nls::GFP]*, **ZX2258:** *pha-1(zx2)*, **ZX2259:** *pha-1(zx2); zxIs20 [pmyo2::ChR2(H134R)::mCherry; pges1::nls::GFP]*, **ZX2260:** *pha-1(zx2); zxIs20 [pmyo-2::ChR2(H134R)::mCherry; pges1::nls::GFP]; zxIs124[pmyo-2::RCaMP1h; pmyo-3::CFP]*, **ZX2261:** *zxEx795 [pmyo-2::RCaMP1h; pmyo-3::CFP]; zxIs20 [pmyo2::ChR2(H134R)::mCherry; pges1::nls::GFP]*, **ZX2264:** *zxIs124[pmyo-2::RCaMP1h; pmyo-3::CFP]*, **ZX2266:** *pha-1(zx2); unc-68(zx5[R414Q])*, **ZX2267:** *pha-1(zx2); unc-68(zx5[R414Q]); zxIs20 [pmyo-2::ChR2(H134R)::mCherry; pges-1::nls::GFP]*, **ZX2269:** *pha-1(zx2); unc-68(zx4[Q4623R]); zxIs20 [pmyo-2::ChR2(H134R)::mCherry; pges-1::nls::GFP]; zxIs20 [pmyo-2::ChR2(H134R)::mCherry; pges-1::nls::GFP]; zxIs124[pmyo-2::RCaMP1h; pmyo-3::CFP]*, ***ZX2270:*** *fkb-2(ok3007) I; unc-68(zx4[Q4623R]); zxIs20 [pmyo2::ChR2(H134R)::mCherry; pges1::nls::GFP]*, **ZX2567:** *csq-1(ok2672); zxIs20[pmyo-2::ChR2(H134R)::mCherry; pges-1::nls::GFP]; zxIs124[pmyo-2::RCaMP1h; pmyo-3::CFP]*, **ZX2568:** *unc-68(r1162)V; zxIs20[pmyo-2::ChR2(H134R)::mCherry; pges-1::nls::GFP]; zxIs124[pmyo-2::RCaMP1h; pmyo-3::CFP]*, **ZX2707:** *csq-1(zx7[K214N]); zxIs20 [pmyo-2::ChR2(H134R)::mCherry; pges-1::nls::GFP]*, **ZX2708:** *unc-68(zx8[P2460S])*, **ZX2718:** *unc-68(zx8[P2460S]); zxIs20 [pmyo-2::ChR2(H134R)::mCherry; pges-1::nls::GFP]*, **ZX2743:** *fkb-2(ok3007) I; unc-68(zx8[P2460S]); zxIs20 [pmyo-2::ChR2(H134R)::mCherry; pges-1::nls::GFP]*, **ZX2744:** *unc-68(zx8[P2460S]); zxIs20 [pmyo-2::ChR2(H134R)::mCherry; pges-1::nls::GFP]; zxIs124[pmyo-2::RCaMP1h; pmyo-3::CFP]*, **ZX2746:** *unc-68(zx9[neutral luminal loop]); zxIs20 [pmyo-2::ChR2(H134R)::mCherry; pges-1::nls::GFP]*, **ZX2748:** *unc-68(zx10[I5231M]); zxIs20 [pmyo-2::ChR2(H134R)::mCherry; pges-1::nls::GFP]*, **ZX2750:** *unc-68(zx11[S2378L]); zxIs20 [pmyo-2::ChR2(H134R)::mCherry; pges-1::nls::GFP]*, **ZX2775:** *unc-68(zx9[neutral luminal loop]); zxIs20 [pmyo-2::ChR2(H134R)::mCherry; pges-1::nls::GFP]; zxIs124[pmyo-2::RCaMP1h; pmyo-3::CFP]*, **ZX2777:** *unc-68(zx11[S2378L]); zxIs20 [pmyo-2::ChR2(H134R)::mCherry; pges-1::nls::GFP]; zxIs124[pmyo-2::RCaMP1h; pmyo-3::CFP]*, **ZX2778:** *fkb-2(ok3007) I; unc-68(zx11[S2378L]); zxIs20 [pmyo-2::ChR2(H134R)::mCherry; pges-1::nls::GFP]*.

### Generation of Point Mutation Alleles

Using a temperature-sensitive embryonic lethal mutation *pha-1(e2123)* co-conversion approach, ZX2256, ZX2258, and ZX2266 were generated (Ward, 2015). Several sgRNA templates were tested for one mutation site. Primers for the generation of these linear PCR-derived sgRNA templates can be found in Supplementary Information. Injection mixes consisted of pJW1285 (60 ng/μl), *pha-1(e2123)* repair template RT01 (50 ng/μl), fusion PCR derived sgRNA template targeting the desired locus (25 ng/μl), and a linear oligo as a repair template carrying the desired mutation (50 ng/μl). Animals that were not embryonic lethal at 25°C were PCR genotyped for successful CRISPR events.

Through mainly following the protocol described by Prior et al. (2017), ZX2708 was generated. sgRNA was generated by annealing TrueGuide™ tracrRNA with crRNA (both Invitrogen, United States), targeting the region of interest (AGAGCAACACCAAGGCAUUC) according to the manufacturer’s manual. The injection mix consisted of sgRNA (5 μM), Cas9 nuclease (5 μM) (TrueCut Cas9 v2, Invitrogen, United States), linear oligo as a repair template (50 ng/μl), *pmyo-3::CFP* (50 ng/μl) as an injection marker, KCl (300 mM), and HEPES (20 mM). Fluorescent progeny was picked and PCR genotyped for successful CRISPR events. For both methods, sgRNA target sites were predicted with the online tool CRISPOR (Concordet and Haeussler, 2018), taking the distance to the point mutation site (< 20 bp), the specificity score, and as few off-targets as possible into account. As repair templates synthesized oligos (Eurofins Genomics, Germany) carrying the desired point mutation as well as 40 bp homology arms were used. The PAM site was deactivated by introducing a silent mutation or, if not possible, by introducing several silent mutations in the sgRNA recognition site. Allele identity was verified by sequencing. Every strain derived from CRISPR needed to be outcrossed with N2 animals before subsequent crossing with ZX1662, ZX2255, ZX2260, and ZX2261. For detailed information on plasmids and repair templates for CRISPR and oligos for sgRNA template generation and genotyping (see Supplementary Information).

### Generation of Transgenic Strains

By integrating the extrachromosomal array of ZX1423 *via* UV radiation (two pulses of 33.3 mJ each) and subsequent backcrossing (4x) with N2 strain, ZX2261 was obtained.

### Determination of Spontaneous Pump Rate on Food and Swimming Cycles in Liquid

One day before experiments, L4 larvae were picked on NGM dishes seeded with OP50-1 culture. Spontaneous pumping of animals on food was video recorded (Powershot G9, Canon, Tokyo, Japan). The NGM dish was manually directed to allow a continuous video recording of a freely moving animal. The grinder movements (pumps) were visually counted for 20 s, to gain a representative number of pumps per animal (about 80–100 pumps for wild type). Loss of focal plane, e.g., due to animals crossing borders of the bacterial lawn, and concomitant impossibility to count pumping, led to exclusion of single animals from the analysis. For the recording of swimming cycles, a 96-well plate was filled with 100 μl NGM and 200 μl M9 buffer. Up to ten young adult animals were picked into one well. Swimming cycles were video recorded (Powershot G9, Canon, Japan) for 60 s and visually counted.

### Optical Paced Electropharyngeogram-Recording With the ScreenChip™ System

One day before experiments, L4 larvae were transferred onto NGM plates seeded with OP50-1 from bacterial suspensions supplemented with ATR (Sigma-Aldrich, United States) with a final concentration of 0.3 mM. Prior to recording, young adult animals were collected in M9 buffer and washed three times. S107 (MedChemExpress, United States) was added in a final concentration of 50 μM (0.1% DMSO) and incubated for 30 min. EPGs were recorded with the ScreenChip™ System (*InVivo* Biosystems (formerly Nemametrix), United States) according to the manufacturer’s manual. Worms were sucked into ScreenChips™ 30 or 40, with respect to their size. Pharyngeal pumping was induced by applying 1.5 mW/mm^2^ of 470 nm light in 50 ms pulses through an inverse microscope (Leica DM IL LED) equipped with a 470 nm LED (KSL 70, Rapp OptoElectronic, Germany), 10x objective (Leica HI Plan I 10x/0.22 ∞/- Ph1), as well as a GFP filter set (450–490 nm excitation, dichroic mirror 510 nm, 515 nm long pass emission, Leica, Germany). Worms in the reservoir of the ScreenChip™ were protected from blue light with red foil (color filter, foil, primary red, Frederiksen, Denmark). Stimulation was performed with 4 Hz pulses for 60 s. Recordings were performed with the software NemAcquire and analyzed with NemAnalysis (both open source from *InVivo* Biosystems). Further analysis was performed in OriginPro (OriginLab, United States).

### Conventional Electropharyngeogram-Recording With Optical Pacing

As for the ScreenChip™ recordings, L4 larvae were transferred 1 day before experiments onto NGM plates seeded with OP50-1 supplemented with 0.3 mM ATR. We performed EPG recordings on cut head preparations; therefore, the head of an animal was cut away from the body with a scalpel (Braun Aesculap, Germany) directly posterior to the terminal bulb. Electrophysiology was performed as described in the study of Schuler et al. (2015). The tip of the worm head was sucked into an EPG-suction electrode. For optical pacing with a 470 nm LED (KSL-70, Rapp Optoelectronics, Germany), the pharynx was positioned below a 60x water immersion objective (LUMIplan FI/IR, 0.9 NA) and an EGFP-ET filter set (AHF Analysentechnik AG, Germany) was used. Recording of EPGs and triggering of light pulses were synchronized by the PatchMaster software (Heka, Germany). The pharynx was stimulated with 470 nm light pulses (1.5–2 mW/mm^2^) in a stress test. The stimulation frequency was increasing stepwise: 1–7 Hz; pulse duration: 35 ms, over a period of 5 s each. The initial stimulation frequency of 1 Hz was held for 15 s to allow a stable rhythm without additional spontaneous pumps. The Review software (Bruxton Corporation, Seattle, WA, United States) was used to translate PatchMaster files. The pump rate was analyzed by AutoEPG (Dillon et al., 2009) (kindly provided by Dr. Christopher James, Embody Biosignals Ltd., United Kingdom), afterward every recording was checked for possible misdetected EPGs. Every EPG showing a clear E (contraction) and R (relaxation) peak was marked and added for subsequent analysis. The highest stimulation frequency (1, 2, 3, 4, 5, 6, or 7 Hz), where every light pulse was followed by each one EPG/pump (for the complete 5 s), was selected as the maximal pump rate per animal.

### Optically Paced Calcium Imaging

We have chosen a genetically encoded calcium indicator (GECI) to specifically target PMCs. Additionally, the nematodes cuticle would prevent uptake of bath-applied dyes. RCaMP1h is a red-shifted GECI that allows a parallel blue-light stimulation of ChR2 (Akerboom et al., 2013).

A day before experiments, L4 larvae were transferred onto NGM plates seeded with OP50-1 from bacterial suspensions supplemented with ATR with a final concentration of 0.3 mM. For imaging of RCaMP1h in *C. elegans* pharynx, young adult animals were placed onto 10% agarose pads (M9 buffer) mounted on microscope slides and were immobilized with polystyrene beads (0.1 μm diameter, at 2.5% w/v, Sigma-Aldrich, United States). Imaging was performed on an inverted microscope (Zeiss Axio Observer Z1), equipped with 40x oil immersion objective (Zeiss EC Plan-NEOFLUAR 40x/N.A. 1.3, Oil DIC ∞/0.17), a 470 nm LED for ChR2 excitation (light pulses of 50 ms, 1.5 mW/mm^2^, 4 Hz), and a 590 nm LED for RCaMP1h excitation (∼0.3 mW/mm^2^) (both KSL-70, Rapp OptoElectronic, Hamburg, Germany), a 80R/20T beam splitter (F21-002, AHF Analysentechnik, Germany), and an EMCCD Camera (Evolve 512 Delta, Photometrics; EM-gain: 25), as well as the following filters: GFP/mCherry (479/585 nm) Dualband ET Filter was combined with a 647/57 nm emission filter and a 605 nm beamsplitter (F56-019, F37-647, and F38-605, respectively, all AHF Analysentechnik, Germany). The terminal bulb of one pharynx was focused. Movies were acquired at 2 ms exposure (ca. 156 fps) and a binning of 4 × 4. The blue-light stimulation started 3 s after the onset of the video-recording and lasted for 10 s to enable the achievement of a plateau phase. Raw image sequences were analyzed *via* ROI selection of signal (terminal bulb of the pharynx) and background, a multi-measure function in ImageJ (National Institutes of Health, United States). Changes in fluorescence were calculated as ΔF/F_0_. The amplitude of ΔF/F_0_ was analyzed during the plateau phase (10–13 s). Importantly, only worms that were able to follow light stimulation have been included for analysis. Experiments were performed in the dark and illumination protocols were applied as short as possible to avoid degradation of photosensitive proteins or substances.

### Statistics

Mean values, SEM, and further statistics (two-sample *t*-test or 1-way ANOVA with the Bonferroni *post-hoc* test) were calculated with OriginPro (OriginLab, United States). Based on experimental settings, every animal was tested only once.

## Results

### Effects of the Insertion of Homologous Catecholaminergic Polymorphic Ventricular Tachycardia Mutations on Spontaneous Pumping and Swimming

After working with extrachromosomal arrays in a previous study (Fischer et al., 2017), we now inserted six different CPVT mutations into *C. elegans unc-68* or *csq-1* (Table 1) *via* different CRISPR-Cas9 approaches (Ward, 2015; Prior et al., 2017) and introduced them into our optogenetic arrhythmia model, a *C. elegans* strain expressing ChR2 in PMCs (Schuler et al., 2015; Fischer et al., 2017).

**FIGURE 1:**
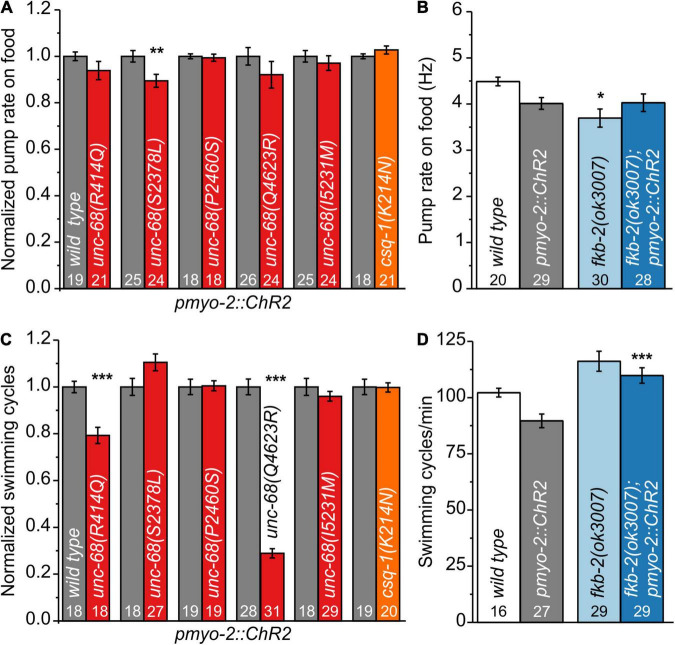
Effect of catecholaminergic polymorphic ventricular tachycardia (CPVT) alleles and *fkb-2* deletion on pharyngeal muscle cells (PMCs) and body-wall muscles (BWMs). **(A)** *Unc-68(S2378L)* shows a reduced normalized pump rate compared to the respective wild type (wt) control strain (young adult animals). **(B)** Deletion of *fkb-2* [*fkb-2(ok3007*)] has no/minor effect on spontaneous pump rate on food. **(C)** Alleles R414Q and Q4623R show a reduction of normalized swimming cycles. **(D)** No significant effect of *fkb-2* deletion on numbers of swimming cycles in comparison to the respective wild-type control. Whereas *fkb-2* deletion in the *pmyo-2::ChR2* background shows an increased number of swimming cycles in comparison to *pmyo-2::ChR2*. Bar graphs display mean values ± SEM. One-way ANOVA with the Bonferroni *post-hoc* test **(B,D)** or two-sample *t*-test for the comparison of only two mean values **(A,C)**: *p ≤ 0.05, **p ≤ 0.01, and ***p ≤ 0.001.

We characterized those genome-edited strains regarding their spontaneous pump rate on food to assess their effect on pharyngeal muscles under normal, non-stressed conditions (Figure 1A). The usual pump rate of young adult wild-type animals is about 4.5 Hz (Figure 1B). One allele, S2378L, showed a significant reduction of the spontaneous pump rate in comparison to wild-type allele (Supplementary Videos 1, 2). Analysis of swimming cycles, reflecting the influence of UNC-68 on the function of BWM and the nervous system, revealed a reduction of the swimming ability in R414Q and Q4623R (Figure 1C). Either the need for movement in the fluid substrate (stress) or the different type of muscle cells, as well as the influence of the nervous system present for BWM, may play a role in these diverse effects. Importantly, we have shown previously that the pharyngeal nervous system does not influence EPG parameters during optogenetically paced pumping (Schuler et al., 2015).

For several CPVT mutations, a disturbance of calstabin2 binding is suggested (Wehrens et al., 2003; Lehnart et al., 2004; Meli et al., 2011). For one of those mutations, R4497C, we observed in a previous study effects in *C. elegans* (Fischer et al., 2017). Thus, we now investigated the effect of calstabin in our model. Calstabin2 stabilizes the closed conformation of RyR (Brillantes et al., 1994). A clear calstabin2 homolog is yet to be identified in *C. elegans*, which contains 8 members of the *fkb* gene class. We tested the presumably best homolog FKB-2 (Fischer et al., 2017) and found, as for the majority of our tested CPVT mutations, minor/no reduction of spontaneous pump rate on food and swimming (Figures 1B,D), indicating no severe effects on BWM or under basal pumping conditions.

### Three Mutations of UNC-68 Cause an Arrhythmic Phenotype

**FIGURE 2:**
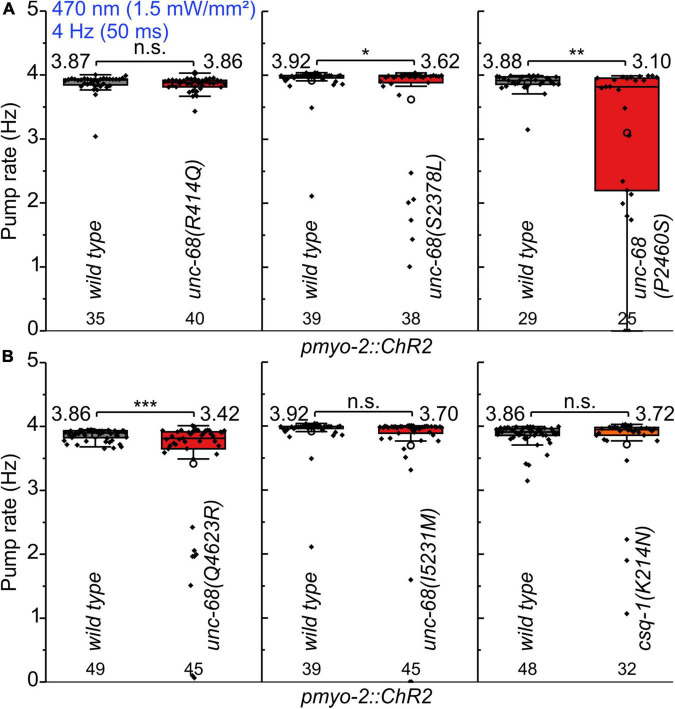
Pumping ability of CPVT mutation bearing strains upon 4 Hz stimulation. **(A,B)** In ScreenChip™ recordings, S2378L, P2460S, and Q4623R show a reduced pump rate compared to wild type in *pmyo-2::ChR2* background. Box plot (25%, 75%; whisker: 1.5 IQR) includes every data point (rhomb), mean (circle), and median (line). Mean values, N-numbers, and significance levels (asterisks) are next to the corresponding box. Two-sample *t*-test: n.s., not significant, *P ≤ 0.05, **P ≤ 0.01, and ***P ≤ 0.001.

**FIGURE 3:**
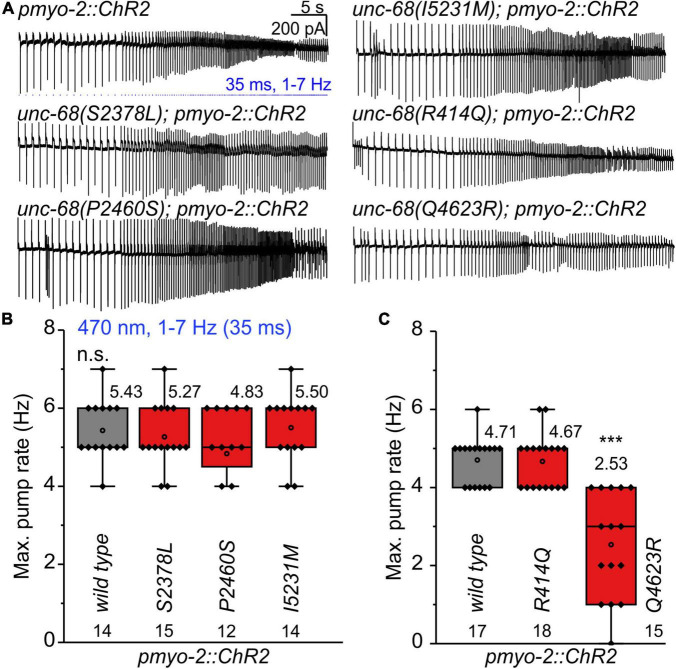
Stress test (1–7 Hz) to determine the maximal pump rate of CPVT mutation-bearing strains. **(A)** Original recordings of electropharyngeograms (EPGs) obtained by electrophysiological characterization of cut-head preparations. Application of 1 Hz blue light pulses (1.5 mW/mm^2^, 35 ms) for 15 s followed by a stepwise increase (every 5 s) from 2 to 7 Hz. **(B,C)** *unc-68(Q4623R)* shows a reduced maximal pump rate in comparison to wt. Since pharyngeal pump ability sometimes varies among different experimental days, likely due to experimental conditions (room temperature, light-sensitivity of ATR), mutant alleles were always compared to the control animals recorded in the same batch of experiments. Box plots include every data point (rhomb), mean (open circle), and median (line). N-numbers, mean values, and significance levels (asterisks), referring to respective wt controls, are located either below or above the corresponding boxes. One-way ANOVA with the Bonferroni *post-hoc* test was performed: n.s., not significant, and ***P ≤ 0.001.

We performed electrophysiological measurements with a microfluidic ScreenChip™ system (*InVivo* Biosystems, United States) that enables higher throughput EPG recordings of intact nematodes in combination with optogenetic stimulation. We observed a significantly reduced ability to follow 4 Hz pacing over a period of 60 s for three of the CPVT alleles *unc-68(S2378L)*, *(P2460S)*, and *(Q4623R)* (Figure 2 and Supplementary Figure 1). This was indicated either by an inability to follow more than about every second stimulus with a pump movement or by a complete stop of pumping. In addition, the maximal pump rate of the different alleles was determined in a stress test with conventional EPG recordings on single cut-head preparations (Raizen and Avery, 1994; Cook et al., 2006). The frequency of light pulses was increased here stepwise from 1 to 7 Hz (Figure 3). Wild-type animals and most of the mutants are able to follow 5 Hz pacing, while *unc-68(Q4623R)*, similar as already shown for *unc-68(R4743C)* (Fischer et al., 2017), follows only about every second pump or stops pumping altogether.

The electrophysiological findings of ScreenChip™ and stress test recordings manifest a “worm arrhythmia” phenotype of several CPVT-related mutations in our optogenetic arrhythmia model. For all alleles showing reduced ability to follow the optogenetic stimulation, a decreased binding of calstabin2 was detected by another group (Wehrens et al., 2003; Lehnart et al., 2004). Nevertheless, a reduced calstabin2 binding of those mutations could not be verified by different groups and is still discussed controversially (Jiang et al., 2005; George et al., 2006).

### Stabilization *via* FKB-2 Is Involved in the Disease-Causing Mechanism of Q4623R and P2460S

**FIGURE 4:**
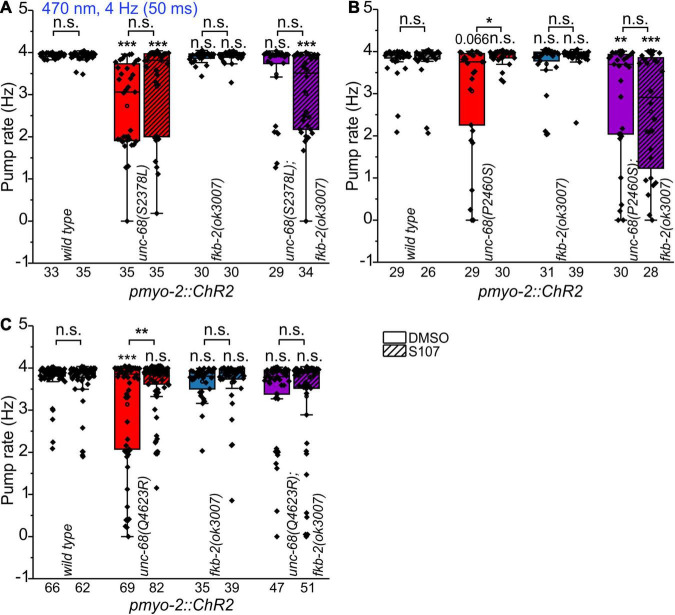
Effect of the benzothiazepine S107. In ScreenChip™ recordings of EPGs under blue light stimulation (4 Hz, 50 ms), the effect of S107 (50 μM, 30 min pre-incubation, shaded boxes) in comparison to the vehicle DMSO (0.1% in M9 buffer) on pump rate of UNC-68 mutants S2378L **(A)**, P2460S **(B)** and Q4623R **(C)** was determined. Alleles have been crossed with a *fkb-2* deletion [*fkb-2(ok3007*), violet boxes] to check for drug-induced UNC-68 stabilization *via* FKB-2. S107 rescues UNC-68 function of Q4623R and P2460S only in FKB-2 wt background. Box plots include mean (open circle) and median (line). Significance levels (asterisks), referring to respective controls (wild type, either with or without S107), are located above the corresponding boxes. Comparison of each strain with and without S107 treatment is indicated above the line pointing on the respective boxes. N-numbers as indicated. One-way ANOVA with the Bonferroni *post-hoc* test was performed: n.s., not significant, *P ≤ 0.05, **P ≤ 0.01, and ***P ≤ 0.001.

To shed light on the disease-causing mechanism of the three CPVT mutations that show effects on pharyngeal pump ability in *C. elegans*, we decided to use a 1,4-benzothiazepine known to enhance calstabin binding and to reduce leakiness of RyR. The 1,4-benzothiazepine derivative JTV519 (a.k.a. K201) is a non-specific blocker of sodium, potassium, and calcium channels (Kaneko et al., 2009), and there are “mixed” results for JTV519, with side-effects outweighing benefits (Driessen et al., 2014). Thus, we decided to test a RyR2-specific compound (S107, CAS 927871-76-9) (Bellinger et al., 2008). A more specific 1,4-benzothiazepine derivative, which in the rodent model enhanced the binding of calstabin2 to the mutant RyR2-R2474S channel, inhibited the channel leak and prevented cardiac arrhythmias (Lehnart et al., 2008). In a previous study, we found, after a 30 min incubation in S107 (50 μM), a complete rescue of the pumping ability of extrachromosomal R4743C mutation bearing nematodes (Fischer et al., 2017). In addition, there was no rescue of pumping ability in the deletion mutant, indicating an *unc-68* (RyR) specific effect of S107, that was not elicited *via* other, unknown channels (Fischer et al., 2017). In ScreenChip™ recordings, during 4 Hz blue light stimulation, we detected a significant rescue of the pumping ability after S107 treatment in two of the three CPVT variants tested (P2460S and Q4623R) (Figure 4). Importantly, this rescue was absent for P2460S in the *fkb-2* deletion background (Figure 4B), indicating that the drug requires the presence of calstabin/FKB-2 to exert beneficial effects on the RyR. While in the case of Q4623R, no significant loss of pumping ability was observed in the *fkb-2* deletion background at all (Figure 4C), as well indicating the involvement of calstabin2 in the disease-causing mechanism. Variant S2378L did not show a rescue upon S107 incubation (Figure 4A), and no effects of S107 appeared in variant I5231M (Supplementary Figure 2). A mutant that *per se* showed no reduction of pump ability in the arrhythmia model (Figure 2).

In line with our results showing the FKB-RyR stabilizing effect of the 1,4-benzothiazepine derivative S107 in UNC-68 variants R4743C (Fischer et al., 2017), P2460S, and Q4623R (homologous to RyR2-R4497C, P2328S, and Q4201R), a decreased binding affinity for calstabin2 is discussed by another group for the respective RyR2 mutations (Wehrens et al., 2003; Lehnart et al., 2004). Whereas other groups could not confirm an alteration of the calstabin2-RyR2 interaction for RyR2-R4497C, -S2246L, -R2474S, -Q4201R, and -I4867M (Jiang et al., 2005; George et al., 2006). In the case of RyR2-I4867M (Figure 2) and -R2474S (Fischer et al., 2017), we observed no “worm arrhythmia” phenotype in EPG recordings of the related nematode allele. Possibly, this could be based on the evocation of not only mild Ca^2+^ handling alterations in those two mutant alleles but also differences between the structure of UNC-68 and RyR2, as well as FKB-2 and calstabin2, might be possible reasons. Nevertheless, in this study, we were able to shed light on the mechanism of UNC-68-P2460S and -Q4623R and proved the controversially discussed involvement of FKB-2/calstabin2 in functional assays in an intact organ of a living animal.

### Several Mutations of *unc-68* Cause Alterations in Ca^2+^ Handling

**FIGURE 5:**
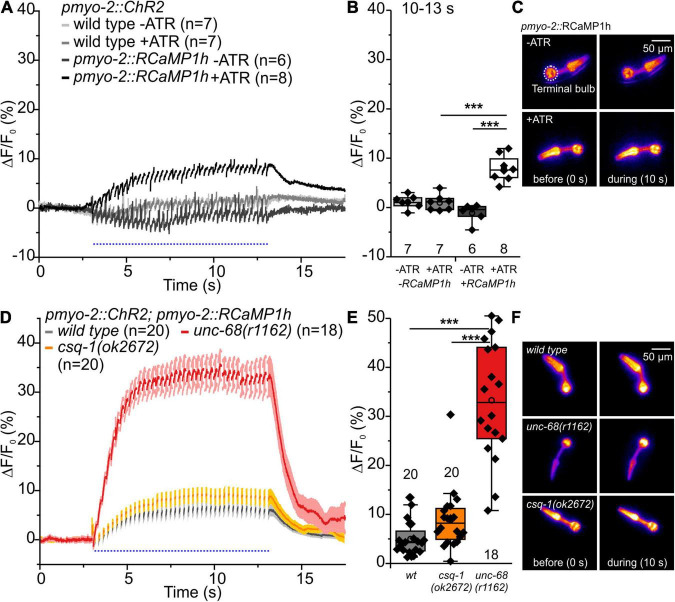
RCaMP1h Ca^2+^ imaging in PMC of *unc-68* and *csq-1* alleles. **(A)** Increase of ΔF/F_0_ during stimulation (4 Hz, 50 ms, 470 nm) and **(B)** amplitude of ΔF/F_0_ during plateau phase (time period: 10-13 s). An increase of ΔF/F_0_ (%) upon light stimulation is only present in ChR2 and RcaMP1h co-expressing pharynxes and in the presence of the ChR2 cofactor ATR. **(C)** False-color representations of fluorescence intensity of ChR2 and RcaMP1h co-expressing pharynxes raised with and without ATR before blue light stimulation (0 s) and during stimulation (∼10 s). The terminal bulb of the pharynx, which is used for analysis, is labeled with a circle. **(D,E)** The null allele of *unc-68*, but not of *csq-1*, leads to an increase of cytosolic Ca^2+^ concentration. **(F)** Exemplary false-color representation of fluorescence intensity. Only worms that were able to follow 4 Hz stimulation have been included for analysis. Depicted is the mean of ΔF/F_0_ (%) without **(A)** or with SEM **(D)**, time periods (∼50 ms), including unspecific peaks induced by blue light stimulation are not displayed here for a better outline. Box plots include every data point (rhomb), mean (open circle), and median (line). N-numbers and significance levels (asterisks) are located either below or above the corresponding boxes. One-way ANOVA with the Bonferroni *post-hoc* test was performed for comparisons of the mean values during the plateau phase of the fluorescence rise (10–13 s): ***P ≤ 0.001.

**FIGURE 6:**
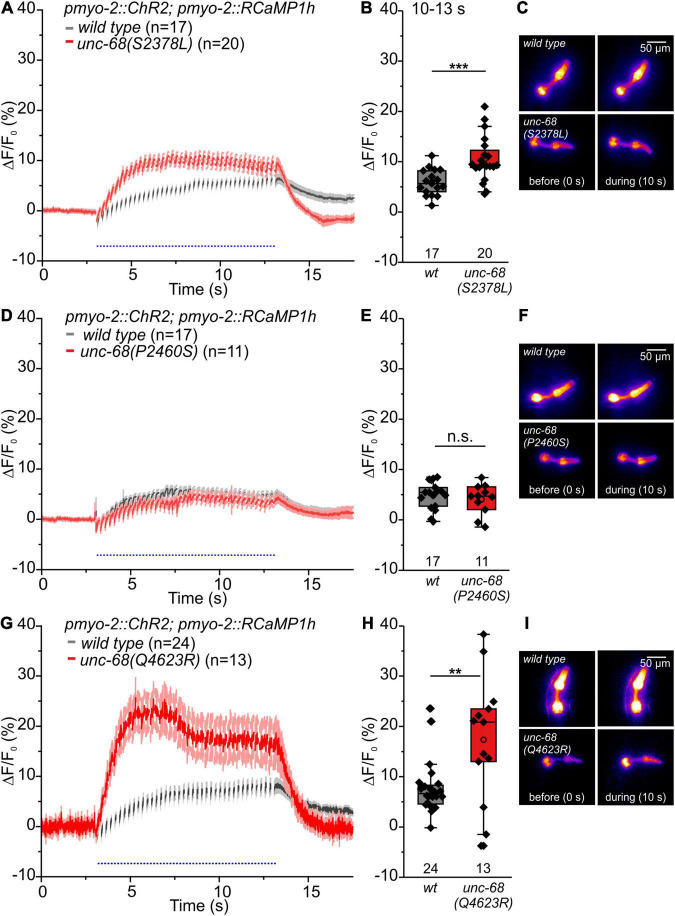
RCaMP1h Ca^2+^ imaging in PMC reveals effects of CPVT mutations inserted into *unc-68* on cytosolic Ca^2+^ concentration. **(A,B,G,H)** Alleles Q4623R and S2378L show an increase of ΔF/F_0_ in comparison to wt animals upon light stimulation (10–13 s). **(D,E)** Whereas allele P2460S does not exhibit a significant change (10–13 s). **(C,F,I)** Exemplary false-color representation of fluorescence intensity. Only worms that were able to follow 4-Hz stimulation have been included for analysis. Depicted is the mean of ΔF/F_0_ (%) with SEM, time periods (∼50 ms), including unspecific peaks induced by blue light stimulation, are not displayed here for better outline **(A,D,G)**. Box plots include every data point (rhomb), mean (open circle), and median (line). N-numbers and significance levels (asterisks) are located either below or above the corresponding boxes. *T*-test was performed for comparisons of the mean values during the plateau phase of the fluorescence rise (10–13 s): n.s., not significant, **P ≤ 0.01, and ***P ≤ 0.001.

We used Ca^2+^ imaging with genetically encoded indicators that we have established earlier (Akerboom et al., 2013) and that can be used in combination with optogenetics to examine intracellular calcium alterations evoked by CPVT mutations. In the present study, we used the Ca^2+^ indicator RCaMP1h, expressed in PMCs of respective CPVT-related alleles, together with ChR2. We stimulated the pharynxes with 50 ms blue light pulses at 4 Hz for a period of 10 s (Figures 5, 6). This allows the generation of a comparable number of pumps (and pump frequency) that can be easily compared within the different alleles and control animals. The light stimulation induced a significant increase of the change (Δ) of F/F_0_ RCamP1h fluorescence, but only when all-trans-retinal (ATR, the cofactor of ChR2) was present (Figures 5A–C). This demonstrates the ability to measure the cytosolic entry of Ca^2+^ during ChR2 stimulation.

Upon blue light stimulation, the *unc-68* null allele *r1162* shows a strong rise of fluorescence (ΔF/F_0_) (Figures 5D–F). Since UNC-68 is not present, this increase likely depends on other proteins, e.g., the voltage-gated Ca^2+^ channel, and demonstrates the disturbance of cytosolic Ca^2+^ concentration and its regulation by compensatory effects in this allele. Indeed, an application of Nemadipine-A, a specific inhibitor of the *C. elegans* voltage-gated Ca^2+^ channel EGL-19 (Kwok et al., 2006; Schuler et al., 2015), reversed the strong Ca^2+^ rise in the *unc-68* null allele (20 μM, Supplementary Figures 3A,C,D). We did not expect a major effect of EGL-19 in CPVT-related mutations since the variants express UNC-68 protein with only one point-mutation, in contrast to the “channel null” allele r1162 that lacks a 3.7 kb area including all transmembrane domains. The null allele exhibits strong effects on swimming and pumping on food (Fischer et al., 2017), while the three CPVT-related mutations show either no or less effect on swimming cycles and no or up to only ∼10% reduction of spontaneous pumping (Figure 1). Thus, their effects, especially in pharynx muscle, are weaker and thus implicating only a minor need for compensatory effects. Consistently, the application of Nemadipine-A (20 μM) does not reverse the strong Ca^2+^ rise of the Q4623R allele (Supplementary Figures 3B–D).

No significant difference of the amplitude of ΔF/F_0_ in comparison to wild type was observed for the *csq-1* deletion, while reduced spontaneous pumping on food and reduced ability to follow optogenetic pacing have previously been shown for this allele. Nevertheless, one has to take into account that the effect of *csq-1(ok2672)* on pumping ability during light stimulation is about 3-fold smaller in comparison to *unc-68(r1162)* (Fischer et al., 2017).

Alleles S2378L and Q4623R exhibit a significant increase of the amplitude of ΔF/F_0_ (Figures 6A–C,G–I), presumably based on UNC-68 leakiness during light stimulated pumping. In the case of allele Q4623R, this increase is reversed by the 1,4-benzothiazepine derivative S107 (Supplementary Figure 4).

In contrast, the amplitude of ΔF/F_0_ of allele P2460S is not increased compared with wild type (Figures 6D–F). Allele P2460S showed the strongest arrhythmic phenotype during 4 Hz stimulation (Figure 2), thus the reduced changes of absolute fluorescence might result from impaired or weaker pumping during stimulation. Also, the deletion of *fkb-2* leads to the reduced amplitude of ΔF/F_0_ (Supplementary Figure 5), suggesting either involvement of a possible second FKB protein or, as discussed for the P2460S allele, due to impaired pumping. We observed no change in the amplitude of ΔF/F_0_ for CPVT-related alleles without an arrhythmic phenotype (Supplementary Figure 6).

### Possible Electrostatic Interaction of UNC-68 and CSQ-1 Plays No Role in Pharyngeal Muscle Cells

We were also interested in the interaction of UNC-68 and CSQ-1 in *C. elegans* pharynx, as differences in the interaction between the two proteins are known for human and *C. elegans*. In mammals, CASQ2 is linked to the RyR through interactions with triadin (Guo and Campbell, 1995) and junctin (Jones et al., 1995). Whereas for junctin no CPVT-causing mutations exist so far, several mutations of triadin have been identified that lead to decreased levels of the protein itself and cause the disease (Roux-Buisson et al., 2012; Rooryck et al., 2015). For the skeletal RyR1, three negatively charged residues of the luminal loop appear to be critical for the association with triadin (Lee et al., 2004). Based on the genomic sequence, there are no obvious homologs of junctin and triadin in *C. elegans*. Therefore, Cho et al. (2007) suggested a physical interaction between UNC-68 and CSQ-1, which might be due to positively charged residues at the C-terminal end of CSQ-1, interacting with negatively charged residues of two luminal UNC-68 loops. In an *in vitro* binding assay, they could show with GST-RyR1 loop 1 and 2 fusion proteins an interaction with CSQ-1. Because of the new insights into RyR structure (Yan et al., 2015; Zalk et al., 2015), there seems only one large luminal loop between S5 and S6 (RyR2: aa 4792-4808), which is also shorter than the formerly suggested loop 2. We replaced *via* CRISPR-Cas9 genome editing negatively charged residues of the proposed S5-S6 luminal loop, which is conserved in *C. elegans unc-68* (aa5156-YVQ**E**G**EE**G**EE**P**D**RKC-5170), with the neutral residue alanine (YVQ**A**G**AA**G**AA**P**A**RKC) to prevent an electrostatic interaction with the positively charged residues of CSQ-1.

**FIGURE 7:**
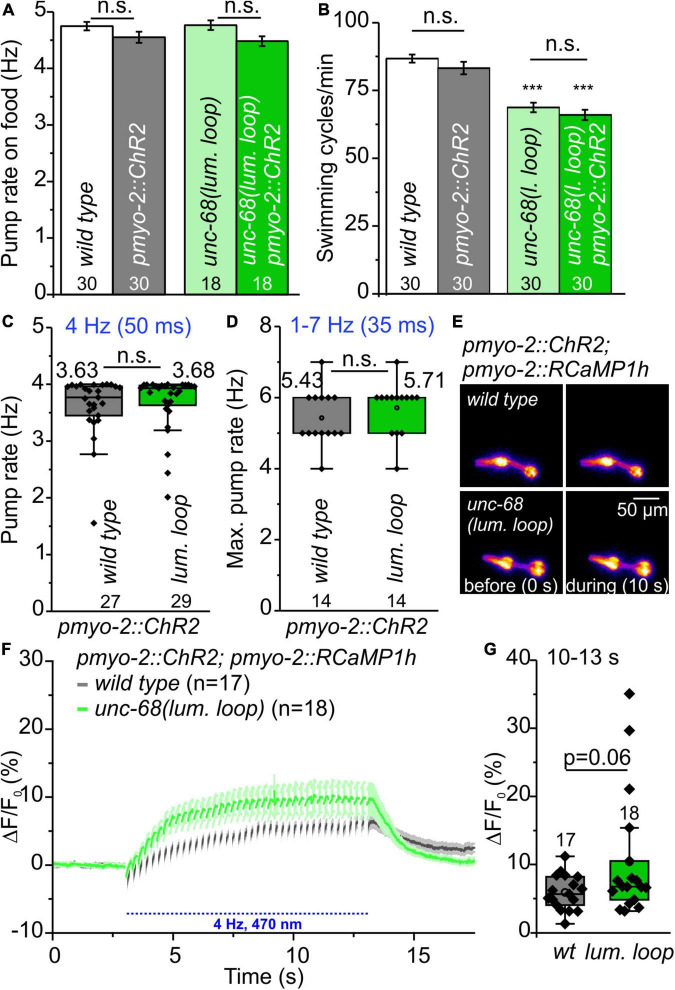
Characterization of neutral amino acid insertion into UNC-68 luminal loop (lum. loop). **(A,B)** No effect on spontaneous pumping on food and reduced number of swimming cycles/min evoked by neutral luminal loop insertion. **(C)** Pumping ability is not reduced in the ScreenChip™ system (4 Hz stimulation, 60 s). **(D)** No effect on maximal pump rate of cut-head preparations in the stress test. **(E)** Exemplary false-color representation of fluorescence intensity. **(F)** Mean ± SEM of the ΔF/F_0_ of RCaMP1h fluorescence before and during light stimulation. **(G)** The amplitudes of ΔF/F_0_ (mean: 10–13 s) show no significant difference between wild-type and neutral luminal loop-expressing animals. Bar graphs represent mean ± SEM, box plots include data points (rhomb), mean (circle), and median (line). N-numbers and significance levels (asterisks) are displayed. One-way ANOVA with the Bonferroni test **(A,B)** or a two-sample *t*-test **(C,D,G)**: n.s., not significant, and ***P ≤ 0.001.

In functional assays, we then investigated the effect of UNC-68 mutated in the putative CSQ direct interaction site. No effect of mutated UNC-68 was observed on spontaneous pumping on food (Figure 7A), whereas swimming was significantly impaired (Figure 7B). Through ScreenChip™ EPG recordings of arrhythmic events, no reduced ability to follow a 4 Hz long-term light stimulation in the optogenetic arrhythmia model was observed (Figure 7C). In addition, the maximal pump rate of cut-head preparations obtained in EPG recordings was not affected (Figure 7D). Ca^2+^ imaging of the UNC-68 neutral luminal loop mutant reveals, as for the *csq-1* deletion (Figures 5D–F), no significant change of intracellular Ca^2+^ concentration (*p* = 0.06) based on RCaMP1h fluorescence (ΔF/F_0_) upon light stimulation (Figures 7E–G). Since the proposed interaction of positively charged residues at the C-terminal end of CSQ-1 and negatively charged residues of UNC-68 luminal loop should be eliminated after an exchange with neutral luminal loop residues, a decreased amount of Ca^2+^ near the channel pore for transport *via* UNC-68 would be expected, subsequently resulting in a decrease of the cytosolic Ca^2+^ concentration. Combined with a lack of obvious differences in the distribution of CSQ-1::CFP in PMCs and BWMs (Supplementary Figure 7), there is likely no influence on CSQ-1 interaction in PMCs by the exchange of luminal loop residues.

## Discussion

We have previously established the *C. elegans* pharynx for the analysis of arrhythmogenic CPVT-mutations, but also of Long QT-8 mutations affecting the Ca_V_1.2 voltage-gated calcium channel (Schuler et al., 2015; Fischer et al., 2017). An optogenetic stimulation of the pharynx *via* ChR2(H134R) enables pacing up to 6 Hz for periods of > 1 min. In the present study, we established the insertion of CPVT mutations *via* CRISPR-Cas9 genome-editing into the optogenetic arrhythmia model. In contrast to extrachromosomal expression, genome-editing allows a stable expression level and a protein distribution that reflects the wild-type condition. In addition, there is no requirement of a manual preselection of transgenic animals, which facilitates higher throughput experiments. For the first time, we implemented here higher throughput electrophysiological recordings of *C. elegans* with optogenetic stimulation by combining a microfluidic ScreenChip™ system (*InVivo* Biosystems) with an Arduino controlled high power LED. This allows precise light stimulation in combination with EPG recordings, succeeded by an automated analysis of the recordings. In sum, it is an excellent option for patient-specific drug testing.

In this study, three of the CPVT alleles *unc-68(S2378L)*, *(P2460S)*, and *(Q4623R)* show an arrhythmic phenotype characterized by a significantly reduced ability to follow 4 Hz pacing in EPG recordings (Figure 2). In addition, alleles S2378L and Q4623R exhibit a significant increase of the amplitude of ΔF/F_0_ in RCaMP1h Ca^2+^ imaging (Figure 6). Corresponding results have been observed in induced pluripotent stem cell-derived cardiomyocytes (iPSC-CM), expressing two different CPVT mutations. Analysis of local Ca^2+^ release events in iPSC-CM loaded with the Ca^2+^ indicator Fluo-4 showed that CPVT cardiomyocytes bearing a mutation in a proposed calstabin binding site (RyR2-F2483I, not conserved in *unc-68*) exhibit higher amplitudes and longer durations of spontaneous local Ca^2+^ release events already at a basal state (Fatima et al., 2011). Also, arrhythmias in iPSC-CM carrying RyR2-R420Q and CASQ2-D307H, evoked by isoproterenol, a synthetic catechol compound, or even sometimes pacing alone, were associated with an elevation in the resting level, probably resulting from a prominent diastolic intracellular Ca^2+^ rise (Novak et al., 2012, 2015).

Up to now, β-blockers are the first therapeutic option for patients with CPVT. Although these substances, which block the binding sites for adrenaline and noradrenaline on β-adrenergic receptors, reduce the occurrence of ventricular tachycardia, 30% of patients still experience cardiac arrhythmias and eventually require cardioverter-defibrillator implantation to prevent cardiac arrest (Priori et al., 2002; Liu et al., 2008). *C. elegans* itself lacks (nor)adrenalin as well as the respective receptors (Sanyal et al., 2004). Tyramine receptor 3 (*tyra-3*), a G-protein-coupled catecholamine receptor for the invertebrate noradrenaline-like neurotransmitters tyramine and octopamine, acts in sensory neurons but is not expressed in PMCs (Bendesky et al., 2011). Nevertheless, since there is a strong motivation to identify more specific drugs to treat CPVT arrhythmias that act directly on the RyR2, particularly to enable treatment with respect to the various mutations affecting the different domains of RyR2 or CASQ2, and their interactions with other proteins, *C. elegans* is a valid model in this respect.

The rycal S107, a 1,4-benzothiazepine derivative, is such a RyR-specific compound (Bellinger et al., 2008). S107 enhances the binding of calstabins to the RyRs. In the rodent model, it inhibited the channel leak and prevented cardiac arrhythmias by enhancing the binding of calstabin2 to mutant RyR2-R2474S channels (Lehnart et al., 2008). Also, in iPSCs carrying the CPVT mutation I4587V, the development of DADs in the presence of isoproterenol was significantly suppressed by S107 (Sasaki et al., 2016). Furthermore, there are observations that fixing RyR2-mediated ER Ca^2+^ leak with the S107 improves cognitive and locomotor function in a murine model of Huntington’s disease (Dridi et al., 2020). In the treatment of RYR1-related myopathies, S107 normalizes an increased calcium leak and activity of calcium-activated proteases (Kushnir et al., 2020). S107 fixes Ca^2+^ leak *via* RyR1 and improves exercise capacity in aging (Andersson et al., 2011) and may represent a new therapeutic approach for Duchenne muscular dystrophy (Bellinger et al., 2009). In a previous study, we found that S107 rescues the pharyngeal pumping ability of CPVT-mutation R4497C [UNC-68(R4743C)] carrying nematodes (Fischer et al., 2017). This is a specific effect of S107 on RyR since no rescue of pumping ability was observed in an *unc-68* deletion mutant (Fischer et al., 2017). For the R4497C mutation, a disturbance of calstabin2 binding is suggested (Wehrens et al., 2003) but is controversially discussed (Liu et al., 2006; Fernández-Velasco et al., 2009). In the present study, three out of five chosen and tested RyR2 mutations are suggested to cause a disturbance of calstabin2 mediated RyR stabilization. In contrast to the two other CPVT mutations included in this study, we observed only for those suggested to bear disturbed calstabin binding an arrhythmia-like phenotype, characterized by a reduced ability to continuously follow light stimulated 4 Hz pacing with a pharyngeal pump movement. The CPVT alleles *unc-68(S2378L)*, *(P2460S)*, and *(Q4623R)* (RyR2-S2246L, -P2328S and -Q4201R, respectively) had a significantly reduced ability to follow 4 Hz pacing over a period of 60 s, indicated by either following about every second stimulus with a pump or a complete stop of pumping (Figure 2). In addition, Q4623R showed a 46% reduced maximal pump rate in comparison to wild type (Figure 3). S107 treatment completely rescued the pumping ability of alleles P2460S and Q4623R (Figure 4). In the case of P2460S, only if FKB-2 was present, which strongly suggests an involvement of calstabin2-mediated stabilization in the disease-causing mechanism of this CPVT-mutation. Interestingly, the allele Q4623R showed no significant reduction of pump rate in the *fkb-2* deletion background, which may hint at different strengths or modes of FKB-2 stabilization involved in the tested alleles. We observed no rescue in nematodes bearing RyR2 mutation S2246L (*unc-68(S2378L)*) after S107 treatment. This is consistent with results obtained in a murine knock-in model of RyR2-S2246L, where treatment with JTV519 (a.k.a. K201), a more unspecific 1,4-benzothiazepine derivative, was without effect on the inducible VT, presumably owing to the fact that the interaction between the 2246 domain (2234–2750 region) and the K201 binding domain (2114–2149 region) was so tight that the drug-binding domain was inaccessible (Suetomi et al., 2011). The respective group could not observe changes in calstabin2 binding but suggests that introduction of CPVT mutation S2246L into the mouse RyR2 induces an aberrant activation of channel gating by forming abnormally tight domain-domain interaction between the two sub-domains located in the central domain, which produces a defective domain unzipping between the N-terminal and the central domain (Suetomi et al., 2011). For RyR2-P2328S and -Q4201R, beneficial effects of a JTV519 treatment have been shown in cell culture (Lehnart et al., 2004; Zhang et al., 2021). Accordingly, mutation-specific drugs may be identified in our optogenetic arrhythmia model that could prevent cardiac arrhythmias based on different mechanisms. Modified 1,4-benzothiazepine derivatives should be tested to access the drug-binding domain of S2246L or possible further mutations and clarify the involvement of a calstabin-mediated stabilization also in those variants.

Interestingly, the deletion of FKB-2 itself had no effect on light stimulated paced pumping (Figure 4) and had only minor effects on spontaneous pumping on food in young adult animals (Figure 1B). Murine knockout models exist for calstabin2 and display different phenotypes. Some calstabin2-deficient mice show no structural cardiac abnormalities and no electrocardiogram abnormalities or arrhythmias at rest but consistently exhibited exercise-induced ventricular arrhythmias that are similar to those observed in patients with CPVT (Wehrens et al., 2003). Another calstabin2-null mouse line showed mild, sex-dependent cardiac hypertrophy but exhibited no stress-induced ventricular tachycardia (Xin et al., 2002; Xiao et al., 2007; Liu et al., 2011). Possibly, differences in the genetic background may account here for the different phenotypes. For *C. elegans*, no clear calstabin2 homolog is known yet and little is known about pharyngeal FKB protein expression. Only for FKB-6, a weak expression in PMCs was shown so far (Richardson et al., 2007). Both, calstabin1 (FKBP12) and calstabin2 (FKBP12.6), bind RyR2 in the heart at a stoichiometry of 4 FKBP per homotetramer (Timerman et al., 1993, 1996). Calstabin1 binds RyR2 with lower affinity but has a higher expression level (Jeyakumar et al., 2001). This suggests that, although calstabin1‘s affinity for RyR2 is lower than that of calstabin2, the greater abundance may result in more calstabin1 bound to RyR2 and could explain why RyR2 is not strongly affected in calstabin2 deficient mice in one of the studies (see review Gonano and Jones, 2017). Possibly, as for RyR2, there might be more than one FKB protein interacting with UNC-68 in PMCs which would explain our results for the FKB-2 deletion.

There must be differences in the interaction of RyR and calsequestrin between *C. elegans* and human, as obvious homologs of junctin and triadin are missing in the *C. elegans* genome. We were not able to prove with functional assays a suggested electrostatic interaction between negatively charged residues of UNC-68 luminal loop and positively charged residues at the C-terminus of CSQ-1 (Cho et al., 2007) in PMCs. Another group observed for a nearby mutation, *unc-68(R5153H)*, strong effects on swimming (Graham et al., 2020). This allele, homologous to a known RyR1-mutation, behaved in liquid as a null mutant but is clearly not a null (Graham et al., 2020). It lies within the transmembrane, pore-forming domain of RyR (Yan et al., 2015) and might be critical to channel function. Thus, possible effects on channel function itself, as seen in swimming assays (Figure 7B), by the exchange of several close-by residues of the luminal loop cannot be excluded here. There is also the possibility that effects might occur in other muscle cells or neurons of the nematode, e.g., due to alternative splicing (Marques et al., 2020), whereas PMCs are not affected. Nevertheless, as demonstrated by a *csq-1* deletion allele, calsequestrin itself influences the pumping and the ability to follow pacing in the optogenetic arrhythmia model (Fischer et al., 2017). Instead of a postulated regulation of the RyR *via* the complex of CASQ, triadin, and/or junctin (Györke and Terentyev, 2008; Qin et al., 2008), only the loss of Ca^2+^ binding and buffering capacity in the *csq-1* deletion might be involved in *C. elegans* PMCs. Based on the differences in the interaction of RyR and calsequestrin between human and *C. elegans*, results obtained for CPVT-related calsequestrin mutations in the pharynx should be carefully considered.

## Conclusion

In conclusion, we were able to shed some light on the mechanism of those CPVT mutations showing effects in *C. elegans* and, hence, to prove the involvement of FKB-2-mediated stabilization of UNC-68 in PMCs. The stabilizing mechanism of calstabin is suggested to be involved here because the specific calstabin-RyR stabilizing drug S107 rescues the pumping ability of P2460S and Q4623R mutations in the nematode, its involvement is already described (or discussed) for the homologous human mutations. Nevertheless, a combination of the calstabin mechanism with one of the other discussed CPVT mechanisms cannot be excluded. Furthermore, the selective effect of S107 on the different CPVT-mutations emphasizes the importance of the development of patient-specific drugs that could be tested in the *C. elegans* optogenetic arrhythmia model with high(er) throughput.

## Data Availability Statement

The raw data supporting the conclusions of this article will be made available by the authors, without undue reservation.

## Author Contributions

CS designed and directed the project. ME, YW, HK, and CS performed the experiments and analyzed the data. ME and CS wrote the article. All authors contributed to the article and approved the submitted version.

## Conflict of Interest

The authors declare that the research was conducted in the absence of any commercial or financial relationships that could be construed as a potential conflict of interest.

## Publisher’s Note

All claims expressed in this article are solely those of the authors and do not necessarily represent those of their affiliated organizations, or those of the publisher, the editors and the reviewers. Any product that may be evaluated in this article, or claim that may be made by its manufacturer, is not guaranteed or endorsed by the publisher.

## References

[B1] Akerboom J. Carreras Calderón N. Tian L. Wabnig S. Prigge M. Tolö J. (2013). Genetically encoded calcium indicators for multi-color neural activity imaging and combination with optogenetics. *Front. Mol. Neurosci.* 6:2. 10.3389/fnmol.2013.00002 23459413 PMC3586699

[B2] Albertson D. G. Thomson J. N. (1976). The pharynx of *Caenorhabditis elegans*. *Philos. Trans. R. Soc. Lond. B Biol. Sci.* 275 299–325. 10.1098/rstb.1976.0085 8805

[B3] Andersson D. C. Betzenhauser M. J. Reiken S. Meli A. C. Umanskaya A. Xie W. (2011). Ryanodine receptor oxidation causes intracellular calcium leak and muscle weakness in aging. *Cell Metab.* 14 196–207. 10.1016/j.cmet.2011.05.014 21803290 PMC3690519

[B4] Arad M. Glikson M. El-Ani D. Monserrat-Inglesias L. (2012). A family with recurrent sudden death and no clinical clue. *Ann. Noninvasive Electrocardiol.* 17 387–393. 10.1111/anec.12024 23094885 PMC6932698

[B5] Bellinger A. M. Reiken S. Carlson C. Mongillo M. Liu X. Rothman L. (2009). Hypernitrosylated ryanodine receptor calcium release channels are leaky in dystrophic muscle. *Nat. Med.* 15 325–330. 10.1038/nm.1916 19198614 PMC2910579

[B6] Bellinger A. M. Reiken S. Dura M. Murphy P. W. Deng S. X. Landry D. W. (2008). Remodeling of ryanodine receptor complex causes “leaky” channels: a molecular mechanism for decreased exercise capacity. *Proc. Natl. Acad. Sci. U.S.A.* 105 2198–2202. 10.1073/pnas.0711074105 18268335 PMC2538898

[B7] Bendesky A. Tsunozaki M. Rockman M. V. Kruglyak L. Bargmann C. I. (2011). Catecholamine receptor polymorphisms affect decision-making in C. elegans. *Nature* 472 313–318. 10.1038/nature09821 21412235 PMC3154120

[B8] Benian G. M. Epstein H. F. (2011). Caenorhabditis elegans muscle: a genetic and molecular model for protein interactions in the heart. *Circ. Res.* 109 1082–1095. 10.1161/CIRCRESAHA.110.237685 21998299

[B9] Brillantes A. B. Ondrias K. Scott A. Kobrinsky E. Ondriasová E. Moschella M. C. (1994). Stabilization of calcium release channel (ryanodine receptor) function by FK506-binding protein. *Cell* 77 513–523. 10.1016/0092-8674(94)90214-3 7514503

[B10] Cho J. H. Ko K. M. Singaruvelu G. Lee W. Kang G. B. Rho S. H. (2007). Functional importance of polymerization and localization of calsequestrin in C. elegans. *J. Cell Sci.* 120 1551–1558. 10.1242/jcs.001016 17405817

[B11] Cho J. H. Oh Y. S. Park K. W. Yu J. Choi K. Y. Shin J. Y. (2000). Calsequestrin, a calcium sequestering protein localized at the sarcoplasmic reticulum, is not essential for body-wall muscle function in *Caenorhabditis elegans*. *J. Cell Sci.* 113 3947–3958. 11058082 10.1242/jcs.113.22.3947

[B12] Concordet J. P. Haeussler M. (2018). CRISPOR: intuitive guide selection for CRISPR/Cas9 genome editing experiments and screens. *Nucleic Acids Res.* 46 W242–W245. 10.1093/nar/gky354 29762716 PMC6030908

[B13] Cook A. Franks C. J. Holden-Dye L. (2006). “Electrophysiological recordings from the pharynx,” in *The C. elegans Research Community*, ed. WormBook (WormBook). Available online at: http://www.wormbook.org 10.1895/wormbook.1.110.1 PMC4781340 18050442

[B14] Davis M. W. Fleischhauer R. Dent J. A. Joho R. H. Avery L. (1999). A mutation in the C. elegans EXP-2 potassium channel that alters feeding behavior. *Science* 286 2501–2504. 10.1126/science.286.5449.2501 10617464 PMC3791429

[B15] Dhindwal S. Lobo J. Cabra V. Santiago D. J. Nayak A. R. Dryden K. (2017). A cryo-EM-based model of phosphorylation- and FKBP12.6-mediated allosterism of the cardiac ryanodine receptor. *Sci. Signal.* 10:eaai8842. 10.1126/scisignal.aai8842 28536302

[B16] Dillon J. Andrianakis I. Bull K. Glautier S. O’Connor V. Holden-Dye L. (2009). AutoEPG: software for the analysis of electrical activity in the microcircuit underpinning feeding behaviour of *Caenorhabditis elegans*. *PLoS One* 4:e8482. 10.1371/journal.pone.0008482 20041123 PMC2795780

[B17] Dridi H. Liu X. Yuan Q. Reiken S. Yehia M. Sittenfeld L. (2020). Role of defective calcium regulation in cardiorespiratory dysfunction in Huntington’s disease. *JCI Insight* 5:e140614. 10.1172/jci.insight.140614 32897880 PMC7566717

[B18] Driessen H. E. Bourgonje V. J. van Veen T. A. Vos M. A. (2014). New antiarrhythmic targets to control intracellular calcium handling. *Neth. Heart J.* 22 198–213. 10.1007/s12471-014-0549-5 24733689 PMC4016334

[B19] Faggioni M. Knollmann B. C. (2012). Calsequestrin 2 and arrhythmias. *Am. J. Physiol. Heart Circ. Physiol.* 302 H1250–H1260. 10.1152/ajpheart.00779.2011 22198169 PMC3311477

[B20] Fang-Yen C. Avery L. Samuel A. D. (2009). Two size-selective mechanisms specifically trap bacteria-sized food particles in *Caenorhabditis elegans*. *Proc. Natl. Acad. Sci. U.S.A.* 106 20093–20096. 10.1073/pnas.0904036106 19903886 PMC2785297

[B21] Fatima A. Xu G. Shao K. Papadopoulos S. Lehmann M. Arnáiz-Cot J. J. (2011). In vitro modeling of ryanodine receptor 2 dysfunction using human induced pluripotent stem cells. *Cell Physiol. Biochem.* 28 579–592. 10.1159/000335753 22178870 PMC3709175

[B22] Fernández-Velasco M. Rueda A. Rizzi N. Benitah J. P. Colombi B. Napolitano C. (2009). Increased Ca^2+^ sensitivity of the ryanodine receptor mutant RyR2*^R4496C^* underlies catecholaminergic polymorphic ventricular tachycardia. *Circ. Res.* 104 201–209. 10.1161/CIRCRESAHA.108.177493 19096022 PMC2796688

[B23] Fischer E. Gottschalk A. Schuler C. (2017). An optogenetic arrhythmia model to study catecholaminergic polymorphic ventricular tachycardia mutations. *Sci. Rep.* 7 17514. 10.1038/s41598-017-17819-8 29235522 PMC5727474

[B24] Franks C. J. Pemberton D. Vinogradova I. Cook A. Walker R. J. Holden-Dye L. (2002). Ionic basis of the resting membrane potential and action potential in the pharyngeal muscle of *Caenorhabditis elegans*. *J. Neurophysiol.* 87 954–961. 10.1152/jn.00233.2001 11826060

[B25] George C. H. Jundi H. Walters N. Thomas N. L. West R. R. Lai F. A. (2006). Arrhythmogenic mutation-linked defects in ryanodine receptor autoregulation reveal a novel mechanism of Ca^2+^ release channel dysfunction. *Circ. Res.* 98 88–97. 10.1161/01.RES.0000199296.70534.7c 16339485

[B26] Gonano L. A. Jones P. P. (2017). FK506-binding proteins 12 and 12.6 (FKBPs) as regulators of cardiac ryanodine receptors: insights from new functional and structural knowledge. *Channels (Austin)* 11 415–425. 10.1080/19336950.2017.1344799 28636428 PMC5626368

[B27] Graham B. Shaw M. A. Hope I. A. (2020). Single amino acid changes in the ryanodine receptor in the human population have effects. *Front. Genet.* 11:37. 10.3389/fgene.2020.00037 32174957 PMC7054344

[B28] Guo W. Campbell K. P. (1995). Association of triadin with the ryanodine receptor and calsequestrin in the lumen of the sarcoplasmic reticulum. *J. Biol. Chem.* 270 9027–9030. 10.1074/jbc.270.16.9027 7721813

[B29] Györke S. Terentyev D. (2008). Modulation of ryanodine receptor by luminal calcium and accessory proteins in health and cardiac disease. *Cardiovasc. Res.* 77 245–255. 10.1093/cvr/cvm038 18006456

[B30] Hamada T. Sakube Y. Ahnn J. Kim D. H. Kagawa H. (2002). Molecular dissection, tissue localization and Ca^2+^ binding of the ryanodine receptor of *Caenorhabditis elegans*. *J. Mol. Biol.* 324 123–135. 10.1016/s0022-2836(02)01032-x 12421563

[B31] Jeyakumar L. H. Ballester L. Cheng D. S. McIntyre J. O. Chang P. Olivey H. E. (2001). FKBP binding characteristics of cardiac microsomes from diverse vertebrates. *Biochem. Biophys. Res. Commun.* 281 979–986. 10.1006/bbrc.2001.4444 11237759

[B32] Jiang D. Wang R. Xiao B. Kong H. Hunt D. J. Choi P. (2005). Enhanced store overload-induced Ca^2+^ release and channel sensitivity to luminal Ca^2+^ activation are common defects of RyR2 mutations linked to ventricular tachycardia and sudden death. *Circ. Res.* 97 1173–1181. 10.1161/01.RES.0000192146.85173.4b 16239587

[B33] Jiang D. Xiao B. Yang D. Wang R. Choi P. Zhang L. (2004). RyR2 mutations linked to ventricular tachycardia and sudden death reduce the threshold for store-overload-induced Ca^2+^ release (SOICR). *Proc. Natl. Acad. Sci. U.S.A.* 101 13062–13067. 10.1073/pnas.0402388101 15322274 PMC516517

[B34] Jones L. R. Zhang L. Sanborn K. Jorgensen A. O. Kelley J. (1995). Purification, primary structure, and immunological characterization of the 26-kDa calsequestrin binding protein (junctin) from cardiac junctional sarcoplasmic reticulum. *J. Biol. Chem.* 270 30787–30796. 10.1074/jbc.270.51.30787 8530521

[B35] Kaneko N. Matsuda R. Hata Y. Shimamoto K. (2009). Pharmacological characteristics and clinical applications of K201. *Curr. Clin. Pharmacol.* 4 126–131. 10.2174/157488409788184972 19442077 PMC2841427

[B36] Kimlicka L. Tung C. C. Carlsson A. C. Lobo P. A. Yuchi Z. Van Petegem F. (2013). The cardiac ryanodine receptor N-terminal region contains an anion binding site that is targeted by disease mutations. *Structure* 21 1440–1449. 10.1016/j.str.2013.06.012 23871484

[B37] Kirchhefer U. Wehrmeister D. Postma A. V. Pohlentz G. Mormann M. Kucerova D. (2010). The human CASQ2 mutation K206N is associated with hyperglycosylation and altered cellular calcium handling. *J. Mol. Cell Cardiol.* 49 95–105. 10.1016/j.yjmcc.2010.03.006 20302875

[B38] Kushnir A. Todd J. J. Witherspoon J. W. Yuan Q. Reiken S. Lin H. (2020). Intracellular calcium leak as a therapeutic target for RYR1-related myopathies. *Acta Neuropathol.* 139 1089–1104. 10.1007/s00401-020-02150-w 32236737 PMC7788518

[B39] Kwok T. C. Ricker N. Fraser R. Chan A. W. Burns A. Stanley E. F. (2006). A small-molecule screen in C. elegans yields a new calcium channel antagonist. *Nature* 441 91–95. 10.1038/nature04657 16672971

[B40] Lee J. M. Rho S. H. Shin D. W. Cho C. Park W. J. Eom S. H. (2004). Negatively charged amino acids within the intraluminal loop of ryanodine receptor are involved in the interaction with triadin. *J. Biol. Chem.* 279 6994–7000. 10.1074/jbc.M312446200 14638677

[B41] Leenhardt A. Denjoy I. Guicheney P. (2012). Catecholaminergic polymorphic ventricular tachycardia. *Circ. Arrhythm. Electrophysiol.* 5 1044–1052. 10.1161/CIRCEP.111.962027 23022705

[B42] Leenhardt A. Lucet V. Denjoy I. Grau F. Ngoc D. D. Coumel P. (1995). Catecholaminergic polymorphic ventricular tachycardia in children. A 7-year follow-up of 21 patients. *Circulation* 91 1512–1519. 10.1161/01.cir.91.5.1512 7867192

[B43] Lehnart S. E. Mongillo M. Bellinger A. Lindegger N. Chen B. X. Hsueh W. (2008). Leaky Ca^2+^ release channel/ryanodine receptor 2 causes seizures and sudden cardiac death in mice. *J. Clin. Invest.* 118 2230–2245. 10.1172/JCI35346 18483626 PMC2381750

[B44] Lehnart S. E. Wehrens X. H. Laitinen P. J. Reiken S. R. Deng S. X. Cheng Z. (2004). Sudden death in familial polymorphic ventricular tachycardia associated with calcium release channel (ryanodine receptor) leak. *Circulation* 109 3208–3214. 10.1161/01.CIR.0000132472.98675.EC 15197150

[B45] Liu N. Colombi B. Memmi M. Zissimopoulos S. Rizzi N. Negri S. (2006). Arrhythmogenesis in catecholaminergic polymorphic ventricular tachycardia: insights from a RyR2 R4496C knock-in mouse model. *Circ. Res.* 99 292–298. 10.1161/01.RES.0000235869.50747.e1 16825580

[B46] Liu N. Ruan Y. Priori S. G. (2008). Catecholaminergic polymorphic ventricular tachycardia. *Prog. Cardiovasc. Dis.* 51 23–30. 10.1016/j.pcad.2007.10.005 18634915

[B47] Liu Y. Chen H. Ji G. Li B. Mohler P. J. Zhu Z. (2011). Transgenic analysis of the role of FKBP12.6 in cardiac function and intracellular calcium release. *Assay Drug Dev. Technol.* 9 620–627. 10.1089/adt.2011.0411 22087651 PMC3232634

[B48] Mango S. E. (2007). “The C. elegans pharynx: a model for organogenesis,” in *The C. elegans Research Community*, ed. WormBook (WormBook). Available online at: http://www.wormbook.org 10.1895/wormbook.1.129.1 PMC4781022 18050503

[B49] Marques F. Thapliyal S. Javer A. Shrestha P. Brown A. E. X. Glauser D. A. (2020). Tissue-specific isoforms of the single C. elegans Ryanodine receptor gene unc-68 control specific functions. *PLoS Genet.* 16:e1009102. 10.1371/journal.pgen.1009102 33104696 PMC7644089

[B50] Maryon E. B. Coronado R. Anderson P. (1996). unc-68 encodes a ryanodine receptor involved in regulating C. elegans body-wall muscle contraction. *J. Cell Biol.* 134 885–893. 10.1083/jcb.134.4.885 8769414 PMC2120954

[B51] Maryon E. B. Saari B. Anderson P. (1998). Muscle-specific functions of ryanodine receptor channels in *Caenorhabditis elegans*. *J. Cell Sci.* 111 2885–2895. 10.1242/jcs.111.19.2885 9730981

[B52] Medeiros-Domingo A. Bhuiyan Z. A. Tester D. J. Hofman N. Bikker H. van Tintelen J. P. (2009). The RYR2-encoded ryanodine receptor/calcium release channel in patients diagnosed previously with either catecholaminergic polymorphic ventricular tachycardia or genotype negative, exercise-induced long QT syndrome: a comprehensive open reading frame mutational analysis. *J. Am. Coll. Cardiol.* 54 2065–2074. 10.1016/j.jacc.2009.08.022 19926015 PMC2880864

[B53] Meli A. C. Refaat M. M. Dura M. Reiken S. Wronska A. Wojciak J. (2011). A novel ryanodine receptor mutation linked to sudden death increases sensitivity to cytosolic calcium. *Circ. Res.* 109 281–290. 10.1161/CIRCRESAHA.111.244970 21659649 PMC3690513

[B54] Nicoll Baines K. Ferreira C. Hopkins P. M. Shaw M. A. Hope I. A. (2017). Aging Effects of *Caenorhabditis elegans* ryanodine receptor variants corresponding to human myopathic mutations. *G3 (Bethesda)* 7 1451–1461. 10.1534/g3.117.040535 28325813 PMC5427508

[B55] Novak A. Barad L. Lorber A. Gherghiceanu M. Reiter I. Eisen B. (2015). Functional abnormalities in iPSC-derived cardiomyocytes generated from CPVT1 and CPVT2 patients carrying ryanodine or calsequestrin mutations. *J. Cell Mol. Med.* 19 2006–2018. 10.1111/jcmm.12581 26153920 PMC4549051

[B56] Novak A. Barad L. Zeevi-Levin N. Shick R. Shtrichman R. Lorber A. (2012). Cardiomyocytes generated from CPVTD307H patients are arrhythmogenic in response to β-adrenergic stimulation. *J. Cell Mol. Med.* 16 468–482. 10.1111/j.1582-4934.2011.01476.x 22050625 PMC3822924

[B57] Prior H. Jawad A. K. MacConnachie L. Beg A. A. (2017). Highly efficient, rapid and Co-CRISPR-independent genome editing in.*Caenorhabditis elegans*. *G3 (Bethesda)* 7 3693–3698. 10.1534/g3.117.300216 28893845 PMC5677160

[B58] Priori S. G. Napolitano C. Memmi M. Colombi B. Drago F. Gasparini M. (2002). Clinical and molecular characterization of patients with catecholaminergic polymorphic ventricular tachycardia. *Circulation* 106 69–74. 10.1161/01.cir.0000020013.73106.d8 12093772

[B59] Priori S. G. Napolitano C. Tiso N. Memmi M. Vignati G. Bloise R. (2001). Mutations in the cardiac ryanodine receptor gene (hRyR2) underlie catecholaminergic polymorphic ventricular tachycardia. *Circulation* 103 196–200. 10.1161/01.cir.103.2.196 11208676

[B60] Qin J. Valle G. Nani A. Nori A. Rizzi N. Priori S. G. (2008). Luminal Ca^2+^ regulation of single cardiac ryanodine receptors: insights provided by calsequestrin and its mutants. *J. Gen. Physiol.* 131 325–334. 10.1085/jgp.200709907 18347081 PMC2279168

[B61] Raizen D. M. Avery L. (1994). Electrical activity and behavior in the pharynx of *Caenorhabditis elegans*. *Neuron* 12 483–495. 8155316 10.1016/0896-6273(94)90207-0 PMC4460247

[B62] Richardson J. M. Dornan J. Opamawutthikul M. Bruce S. Page A. P. Walkinshaw M. D. (2007). Cloning, expression and characterisation of FKB-6, the sole large TPR-containing immunophilin from C. elegans. *Biochem. Biophys. Res. Commun.* 360 566–572. 10.1016/j.bbrc.2007.06.080 17610845

[B63] Rooryck C. Kyndt F. Bozon D. Roux-Buisson N. Sacher F. Probst V. (2015). New Family With catecholaminergic polymorphic ventricular tachycardia linked to the triadin gene. *J. Cardiovasc. Electrophysiol.* 26 1146–1150. 10.1111/jce.12763 26200674

[B64] Roux-Buisson N. Cacheux M. Fourest-Lieuvin A. Fauconnier J. Brocard J. Denjoy I. (2012). Absence of triadin, a protein of the calcium release complex, is responsible for cardiac arrhythmia with sudden death in human. *Hum. Mol. Genet.* 21 2759–2767. 10.1093/hmg/dds104 22422768 PMC3363337

[B65] Sanyal S. Wintle R. F. Kindt K. S. Nuttley W. M. Arvan R. Fitzmaurice P. (2004). Dopamine modulates the plasticity of mechanosensory responses in *Caenorhabditis elegans*. *EMBO J.* 23 473–482. 10.1038/sj.emboj.7600057 14739932 PMC1271763

[B66] Sasaki K. Makiyama T. Yoshida Y. Wuriyanghai Y. Kamakura T. Nishiuchi S. (2016). Patient-Specific human induced pluripotent stem cell model assessed with electrical pacing validates S107 as a potential therapeutic agent for catecholaminergic polymorphic ventricular tachycardia. *PLoS One* 11:e0164795. 10.1371/journal.pone.0164795 27764147 PMC5072719

[B67] Schuler C. Fischer E. Shaltiel L. Steuer Costa W. Gottschalk A. (2015). Arrhythmogenic effects of mutated L-type Ca ^2+^-channels on an optogenetically paced muscular pump in *Caenorhabditis elegans*. *Sci. Rep.* 5 14427. 10.1038/srep14427 26399900 PMC4585839

[B68] Shtonda B. Avery L. (2005). CCA-1, EGL-19 and EXP-2 currents shape action potentials in the *Caenorhabditis elegans* pharynx. *J. Exp. Biol.* 208 2177–2190. 10.1242/jeb.01615 15914661 PMC1351090

[B69] Suetomi T. Yano M. Uchinoumi H. Fukuda M. Hino A. Ono M. (2011). Mutation-linked defective interdomain interactions within ryanodine receptor cause aberrant Ca^2 +^release leading to catecholaminergic polymorphic ventricular tachycardia. *Circulation* 124 682–694. 10.1161/CIRCULATIONAHA.111.023259 21768539 PMC3153588

[B70] Tester D. J. Spoon D. B. Valdivia H. H. Makielski J. C. Ackerman M. J. (2004). Targeted mutational analysis of the RyR2-encoded cardiac ryanodine receptor in sudden unexplained death: a molecular autopsy of 49 medical examiner/coroner’s cases. *Mayo. Clin. Proc.* 79 1380–1384. 10.4065/79.11.1380 15544015

[B71] Timerman A. P. Ogunbumni E. Freund E. Wiederrecht G. Marks A. R. Fleischer S. (1993). The calcium release channel of sarcoplasmic reticulum is modulated by FK-506-binding protein. *J. Biol. Chem.* 268 22992–22999. 10.1016/s0021-9258(19)49416-7 7693682

[B72] Timerman A. P. Onoue H. Xin H. B. Barg S. Copello J. Wiederrecht G. (1996). Selective binding of FKBP12.6 by the cardiac ryanodine receptor. *J. Biol. Chem.* 271 20385–20391. 10.1074/jbc.271.34.20385 8702774

[B73] van der Werf C. Kannankeril P. J. Sacher F. Krahn A. D. Viskin S. Leenhardt A. (2011). Flecainide therapy reduces exercise-induced ventricular arrhythmias in patients with catecholaminergic polymorphic ventricular tachycardia. *J. Am. Coll. Cardiol.* 57 2244–2254. 10.1016/j.jacc.2011.01.026 21616285 PMC3495585

[B74] Ward J. D. (2015). Rapid and precise engineering of the *Caenorhabditis elegans* genome with lethal mutation co-conversion and inactivation of NHEJ repair. *Genetics* 199 363–377. 10.1534/genetics.114.172361 25491644 PMC4317648

[B75] Wehrens X. H. Lehnart S. E. Huang F. Vest J. A. Reiken S. R. Mohler P. J. (2003). FKBP12.6 deficiency and defective calcium release channel (ryanodine receptor) function linked to exercise-induced sudden cardiac death. *Cell* 113 829–840. 10.1016/s0092-8674(03)00434-3 12837242

[B76] Wehrens X. H. Lehnart S. E. Reiken S. R. Deng S. X. Vest J. A. Cervantes D. (2004). Protection from cardiac arrhythmia through ryanodine receptor-stabilizing protein calstabin2. *Science* 304 292–296. 10.1126/science.1094301 15073377

[B77] Xiao J. Tian X. Jones P. P. Bolstad J. Kong H. Wang R. (2007). Removal of FKBP12.6 does not alter the conductance and activation of the cardiac ryanodine receptor or the susceptibility to stress-induced ventricular arrhythmias. *J. Biol. Chem.* 282 34828–34838. 10.1074/jbc.M707423200 17921453 PMC2760432

[B78] Xin H. B. Senbonmatsu T. Cheng D. S. Wang Y. X. Copello J. A. Ji G. J. (2002). Oestrogen protects FKBP12.6 null mice from cardiac hypertrophy. *Nature* 416 334–338. 10.1038/416334a 11907581

[B79] Yan Z. Bai X. Yan C. Wu J. Li Z. Xie T. (2015). Structure of the rabbit ryanodine receptor RyR1 at near-atomic resolution. *Nature* 517 50–55. 10.1038/nature14063 25517095 PMC4338550

[B80] Yuchi Z. Yuen S. M. Lau K. Underhill A. Q. Cornea R. L. Fessenden J. D. (2015). Crystal structures of ryanodine receptor SPRY1 and tandem-repeat domains reveal a critical FKBP12 binding determinant. *Nat. Commun.* 6 7947. 10.1038/ncomms8947 26245150 PMC4530471

[B81] Zalk R. Clarke O. B. des Georges A. Grassucci R. A. Reiken S. Mancia F. (2015). Structure of a mammalian ryanodine receptor. *Nature* 517 44–49. 10.1038/nature13950 25470061 PMC4300236

[B82] Zhang X. H. Wei H. Xia Y. Morad M. (2021). Calcium signaling consequences of RyR2 mutations associated with CPVT1 introduced via CRISPR/Cas9 gene editing in human-induced pluripotent stem cell-derived cardiomyocytes: Comparison of RyR2-R420Q, F2483I, and Q4201R. *Heart Rhythm* 18 250–260. 10.1016/j.hrthm.2020.09.007 32931925 PMC7893824

