# Supplementary material for: An Optogenetic Arrhythmia Model—Insertion of Several Catecholaminergic Polymorphic Ventricular Tachycardia Mutations Into Caenorhabditis elegans UNC-68 Disturbs Calstabin-Mediated Stabilization of the Ryanodine Receptor Homolog
Source: Front Physiol. 2022 Mar 25;13:691829. doi: 10.3389/fphys.2022.691829 (PMC8990320; doi:10.3389/fphys.2022.691829)
Supplement: Supplementary file 3 [file Data_Sheet_1.DOCX]

# Plasmids

| **Name** | **description** | **purpose** | **source** |
| --- | --- | --- | --- |
| **pJW1285** | peft-3:::Cas9; pU6::pha-1 sgRNA template | pha-1(e2123) co-conversion CRISPR approach | Addgene #61252 |
| **pJW1310** | pU6 | Template for cloning U6 promoter to generate new PU6::sgRNAs by PCR-fusion | Addgene #61253 |
| **pJW1311** | sgRNA template scaffhold | Template for cloning sgRNA(F+E) to generate new PU6::sgRNAs by PCR-fusion | Addgene #61254 |

# Repair templates

| **Name** | **Sequence** | **Purpose and strain of generation** |
| --- | --- | --- |
| **RT01** | Caaaatacgaatcgaagactcaaaaagagtatgctgtatgattacagatgttcatcaagttatt cataaatcattgatag | Co-conversion  ZX2258: *pha-1(zx2)* |
| **RT07** | Ctttcatcaatttctgtgaagacactatttttgaaatgcgtcatgccgcagcgatttcttctggaga tagtgacaccaag | ZX2256: *unc-68(zx4[Q4623R])* |
| **RT11** | Ctttagaagaagaatccaaatcagctagagtcattcagaaatgttcatcggtgcttaacaagtttt taaaaggaatcgatgcattgcaacttgaaggaaaccaat | ZX2266: *unc-68(zx5[R414Q])* |
| **RT15** | Agaaaatgcaaatcttgtcattcgtctgctgattcgcagaagcgaatgtcttggtgttgctctgaa aggagaaggacaaggattgttctct | ZX2708: *unc-68(zx8[P2460S])* |

# Oligos

| **Name** | **Sequence** | **Description/purpose** |
| --- | --- | --- |
| **oME016** | attgtgttcgttgagtgacc | PU-6 forward primer for sgRNA template |
| **oME017** | aagacatctcgcaataggagg | PU-6 reverse primer for sgRNA template |
| **oME018** | aaaaataggcgtatcacgagg | sgRNA template reverse primer |
| **oME020** | cctcctattgcgagatgtcttgATGCCGCGGCGATTTCTTCTgtttaagagctatgctgg | *unc-68(Q4623R)* sgRNA template 1 |
| **oME021** | cctcctattgcgagatgtcttgTGCCGCGGCGATTTCTTCTGgtttaagagctatgctgg | *unc-68(Q4623R)* sgRNA template 2 |
| **oME022** | cctcctattgcgagatgtcttgTTTTGAAATGCAGCATGCCGgtttaagagctatgctgg | *unc-68(Q4623R)* sgRNA template 3 |
| **oME024** | cgatggaaaagttgactacatg | Genotyping *unc-68(Q4623R)* forward primer |
| **oME025** | ctgcagaagtttgttgcaag | Genotyping *unc-68(Q4623R)* reverse primer |
| **oME026** | caatttggcagccattcatgtg | Genotyping *pha-1(e2123)* forward primer |
| **oME027** | tcgcgcactactgaatcagagtc | Genotyping *pha-1(e2123)* reverse primer |
| **oME028** | cacgattgctccggcttttg | Genotyping *fkb-2(ok3007)* forward primer |
| **oME029** | tatggagaggttgcacacgg | Genotyping *fkb-2(ok3007)* reverse primer |
| **oME035** | cctcctattgcgagatgtcttgCCTCCTATTGCGAGATGTCTTGgtttaagagctatgctgg | *unc-68(R414Q)* sgRNA template 1 |
| **oME036** | cctcctattgcgagatgtcttgCCAAATCAGCTAGAGTCATTgtttaagagctatgctgg | *unc-68(R414Q)* sgRNA template 2 |
| **oME037** | cctcctattgcgagatgtcttgAGTCATTAGGAAATGTTCATgtttaagagctatgctgg | *unc-68(R414Q)* sgRNA template 3 |
| **oME038** | gaaagatggtcacatggatg | Genotyping *unc-68(R414Q)* forward primer |
| **oME039** | cgattaatccagcgaaatcag | Genotyping *unc-68(R414Q)* reverse primer |
| **oME089** | cctcctattgcgagatgtcttgAGAGCAACACCAAGGCATTCGGGgtttaagagctatgctgg | *unc-68(P2460S)* sgRNA template 1 |
| **oME090** | cctcctattgcgagatgtcttgCAGAGCAACACCAAGGCATTCGGgtttaagagctatgctgg | *unc-68(P2460S)* sgRNA template 2 |
| **oME091** | cctcctattgcgagatgtcttgCTCCTTTCAGAGCAACACCAAGGgtttaagagctatgctgg | *unc-68(P2460S)* sgRNA template 3 |
| **oME092** | cctcctattgcgagatgtcttgATTCGCAGACCCGAATGCCTTGGgtttaagagctatgctgg | *unc-68(P2460S)* sgRNA template 4 |
| **oME093** | gagatgaattgaaagagaaag | Genotyping *unc-68(P2460S)* forward primer |
| **oME094** | ctgtatagccattggatcag | Genotyping *unc-68(P2460S)* reverse primer |
