# Supplementary material for: An Optogenetic Arrhythmia Model—Insertion of Several Catecholaminergic Polymorphic Ventricular Tachycardia Mutations Into Caenorhabditis elegans UNC-68 Disturbs Calstabin-Mediated Stabilization of the Ryanodine Receptor Homolog
Source: Front Physiol. 2022 Mar 25;13:691829. doi: 10.3389/fphys.2022.691829 (PMC8990320; doi:10.3389/fphys.2022.691829)
Supplement: Supplementary file 4 [file Data_Sheet_2.PDF]

```
oldUNC-68     1 MADKEEQGGGEQDDVSFLRTGDIVCLSCVASHNRDGVLGSERVCLCTEGFGNRMCTLENV
UNC-68        1 MADKEEQGGGEQDDVSFLRTGDIVCLSCVASHNRDGVLGSERVCLCTEGFGNRMCTLENV
RYR1_HUMAN    1 -----MGDAEGEDEVQFLRTDDEVVLQCSATVLKE----QLKLCLAAEGFGNRLCFLEPT
RYR2_HUMAN    1 ---MADGGEG-EDEIQFLRTDDEVVLQCTATIHKE----QQKLCLAAEGFGNRLCFLEST
RYR3_HUMAN    1 ---MAEGGEGGEDEIQFLRTEDEVVLQCIATIHKE----QRKFCLAAEGLGNRLCFLEPT

oldUNC-68    61 SD-KDIPPDIAMCMLYIDNALSMRALQEMMSADSDHKSA-------------SGAGGHKT
UNC-68       61 SD-KDIPPDIAMCMLYIDNALSMRALQEMMSADSDHKSA-------------SGAGGHKT
RYR1_HUMAN   52 SNAQNVPPDLAICCFVLEQSLSVRALQEMLANTVEAGVE------------SSQGGGHRT
RYR2_HUMAN   53 SNSKNVPPDLSICTFVLEQSLSVRALQEMLANTVEKSEGQVDVEKWKFMMKTAQGGGHRT
RYR3_HUMAN   54 SEAKYIPPDLCVCNFVLEQSLSVRALQEMLANTGENGGE-----------GAAQGGGHRT

oldUNC-68   107 LLYGHAVQLKHVQSEMYLACLSSCSS-NDKLAFDVGVQETNEGEACWWTIHPASKQRSEG
UNC-68      107 LLYGHAVQLKHVQSEMYLACLSSCSS-NDKLAFDVGVQETNEGEACWWTIHPASKQRSEG
RYR1_HUMAN  100 LLYGHAILLRHAHSRMYLSCLTTSRSMTDKLAFDVGLQEDATGEACWWTMHPASKQRSEG
RYR2_HUMAN  113 LLYGHAILLRHSYSGMYLCCLSTSRSSTDKLAFDVGLQEDTTGEACWWTIHPASKQRSEG
RYR3_HUMAN  103 LLYGHAVLLRHSFSGMYLTCLTTSRSQTDKLAFDVGLREHATGEACWWTIHPASKQRSEG

oldUNC-68   166 EKVRVGDDVILVSVATERYLHMAYSKG-YMVIASFHQTLWNIQSVSSGSMRTRNMGFLFG
UNC-68      166 EKVRVGDDVILVSVATERYLHMAYSKG-YMVIASFHQTLWNIQSVSSGSMRTRNMGFLFG
RYR1_HUMAN  160 EKVRVGDDIILVSVSSERYLHLSTASGELQVDASFMQTLWNMNPICS----RCEEGFVTG
RYR2_HUMAN  173 EKVRVGDDLILVSVSSERYLHLSYGNGSLHVDAAFQQTLWSVAPISSG--SEAAQGYLIG
RYR3_HUMAN  163 EKVRIGDDLILVSVSSERYLHLSVSNGNIQVDASFMQTLWNVHPTCSG--SSIEEGYLLG

oldUNC-68   225 NDVLRLFHGN-DECLTIPENWSEHPQHNMVIYEGGAAVTQARSLWRVELIRMKWHGALVG
UNC-68      225 NDVLRLFHGN-DECLTIPENWSEHPQHNMVIYEGGAAVTQARSLWRVELIRMKWHGALVG
RYR1_HUMAN  216 GHVLRLFHGHMDECLTISPAD-SDDQRRLVYYEGGAVCTHARSLWRLEPLRISWSGSHLR
RYR2_HUMAN  231 GDVLRLLHGHMDECLTVPSGEHGEEQRRTVHYEGGAVSVHARSLWRLETLRVAWSGSHIR
RYR3_HUMAN  221 GHVVRLFHGH-DECLTIPSTDQNDSQHRRIFYEAGGAGTRARSLWRVEPLRISWSGSNIR

oldUNC-68   284 WEQVFRIKHITSGRYLGVLDN-SVQLYHKEKADFDLTAFVMCQNK--DPKKQMLDEKEEE
UNC-68      284 WEQVFRIKHITSGRYLGVLDN-SVQLYHKEKADFDLTAFVMCQNK--DPKKQMLDEKEEE
RYR1_HUMAN  275 WGQPLRVRHVTTGQYLALTEDQGLVVVDASKAHTKATSFCFRIS---KEKLDVAPKRDVE
RYR2_HUMAN  291 WGQPFRLRHVTTGKYLSLMEDKNLLLMDKEKADVKSTAFTFRSS---KEKLDVGVRKEVD
RYR3_HUMAN  280 WGQAFRLRHLTTGHYLALTEDQGLILQDRAKSDTKSTAFSFRASKELKEKLDSSHKRDIE

oldUNC-68   341 GMGNATIRYGETNAFIQHVKTQLWLSYQTTEVTKKGLGKVEEKKAVALKDGHMDDCYTFF
UNC-68      341 GMGNATIRYGETNAFIQHVKTQLWLSYQTTEVTKKGLGKVEEKKAVALKDGHMDDCYTFF
RYR1_HUMAN  332 GMGPPEIKYGESLCFVQHVASGLWLTYAAPDPKALRLGVLK-KKAMLHQEGHMDDALSLT
RYR2_HUMAN  348 GMGTSEIKYGDSVCYIQHVDTGLWLTYQSVDVKSVRMGSIQ-RKAIMHHEGHMDDGISLS
RYR3_HUMAN  340 GMGVPEIKYGDSVCFVQHIASGLWVTYKAQDAKTSRLGPLK-RKVILHQEGHMDDGLTLQ

oldUNC-68   401 MALEEESKSARVIRKCSSVLNKFLKGIDALQLEGNQST--DWTRVDLNEVLKLMEDLIEY
UNC-68      401 MALEEESKSARVIRKCSSVLNKFLKGIDALQLEGNQST--DWTRVDLNEVLKLMEDLIEY
RYR1_HUMAN  391 RCQQEESQAARMIHSTNGLYNQFIKSLDSFSGKPRGSGPPAGTALPIEGVILSLQDLIIY
RYR2_HUMAN  407 RSQHEESRTARVIRSTVFLFNRFIRGLDALSKKAKAST----VDLPIESVSLSLQDLIGY
RYR3_HUMAN  399 RCQREESQAARIIRNTTALFSQFVS-----GNNRTAAP----ITLPIEEVLQTLQDLIAY

oldUNC-68   459 FAQPNDEQDFEEKQNHLRALRSRQDLFQEEGVLNMILDTIDKFSQMEALPDFAGLIGEET
UNC-68      459 FAQPNDEQDFEEKQNHLRALRSRQDLFQEEGVLNMILDTIDKFSQMEALPDFAGLIGEET
RYR1_HUMAN  451 FEPPSEDLQHEEKQSKLRSLRNRQSLFQEEGMLSMVLNCIDRLNVYTTAAHFAEFAGEEA
RYR2_HUMAN  463 FHPPDEHLEHEDKQNRLRALKNRQNLFQEEGMINLVLECIDRLHVYSSAAHFADVAGREA
RYR3_HUMAN  450 FQPPEEEMRHEDKQNKLRSLKNRQNLFKEEGMLALVLNCIDRLNVYNSVAHFAGIAREES

oldUNC-68   519 HVKWEQISTYLYLLVAAMIKGNHYNCAQFASAQRLDWLFGRLSNPQSAEGILDVLYCVLT
UNC-68      519 HVKWEQISTYLYLLVAAMIKGNHYNCAQFASAQRLDWLFGRLSNPQSAEGILDVLYCVLT
RYR1_HUMAN  511 AESWKEIVNLLYELLASLIRGNRSNCALFS--TNLDWLVSKLDRLEASSGILEVLYCVLI
RYR2_HUMAN  523 GESWKSILNSLYELLAALIRGNRKNCAQFS--GSLDWLISRLERLEASSGILEVLHCVLV
RYR3_HUMAN  510 GMAWKEILNLLYKLLAALIRGNRNNCAQFS--NNLDWLISKLDRLESSSGILEVLHCILT
```

```
oldUNC-68    579 ESPEALNMINEGHIRSVISLLEKVGRDPKVLDVLSSLCEGNGMAVRSSQNLITQYLLPGK
UNC-68       579 ESPEALNMINEGHIRSVISLLEKVGRDPKVLDVLSSLCEGNGMAVRSSQNLITQYLLPGK
RYR1_HUMAN   569 ESPEVLNIIQENHIKSIISLLDKHGRNHKVLDVLCSLCVCNGVAVRSNQDLITENLLPGR
RYR2_HUMAN   581 ESPEALNIIKEGHIKSIISLLDKHGRNHKVLDVLCSLCVCHGVAVRSNQHLICDNLLPGR
RYR3_HUMAN   568 ESPEALNLIAEGHIKSIISLLDKHGRNHKVLDILCSLCLCNGVAVRANQNLICDNLLPRR

oldUNC-68    639 DLLLQTSMRDHVSSMMPNVMLGVVEGSALFRKWYFEAEVEHIET-MTKQTPYLRIGWANS
UNC-68       639 DLLLQTSMRDHVSSMMPNVMLGVVEGSALFRKWYFEAEVEHIET-MTKQTPYLRIGWANS
RYR1_HUMAN   629 ELLLQTNLINYVTSIRPNIFVGRAEGTTQYSKWYFEVMVDEVTPFLTAQATHLRVGWALT
RYR2_HUMAN   641 DLLLQTRLVNHVSSMRPNIFLGVSEGSAQYKKWYYELMVDHTEPFVTAEATHLRVGWAST
RYR3_HUMAN   628 NLLLQTRLINDVTSIRPNIFLGVAEGSAQYKKWYFELIIDQVDPFLTAEPTHLRVGWASS

oldUNC-68    698 VGFKPFPGSGDKMGCNGVGDDFYSYGFDGKSMYFGGKSRRVG---HKLLEKGDVIGCSID
UNC-68       698 VGFKPFPGSGDKMGCNGVGDDFYSYGFDGKSMYFGGKSRRVG---HKLLEKGDVIGCSID
RYR1_HUMAN   689 EGYTPYPGAGEGWGGNGVGDDLYSYGFDGLHLWTGHVARPVTSPGQHLLAPEDVISCCLD
RYR2_HUMAN   701 EGYSPYPGGGEEWGGNGVGDDLFSYGFDGLHLWSGCIARTVSSPNQHLLRTDDVISCCLD
RYR3_HUMAN   688 SGYAPYPGGGEGWGGNGVGDDLYSYGFDGLHLWSGRIPRAVASINQHLLRSDDVVSCCLD

oldUNC-68    755 LTIPEIKFSVNGTYMSGSFKKFNIDGYFFPVMSLSAKVSCRFILGGNQGRLRYGPPTGFS
UNC-68       755 LTIPEIKFSVNGTYMSGSFKKFNIDGYFFPVMSLSAKVSCRFILGGNQGRLRYGPPTGFS
RYR1_HUMAN   749 LSVPSISFRINGCPVQGVFESFNLDGLFFPVVSFSAGVKVRFLLGGRHGEFKFLPPPGYA
RYR2_HUMAN   761 LSAPSISFRINGQPVQGMFENFNIDGLFFPVVSFSAGIKVRFLLGGRHGEFKFLPPPGYA
RYR3_HUMAN   748 LGVPSISFRINGQPVQGMFENFNTDGLFFPVMSFSAGVKVRFLMGGRHGEFKFLPPSGYA

oldUNC-68    815 AVVEAVN--GELQITDCLSFGDLGKNIFSGP-QTIFNNLEPFIPTPIDVSATQLNHHATE
UNC-68       815 AVVEAVN--GELQITDCLSFGDLGKNIFSGP-QTIFNNLEPFIPTPIDVSATQLNHHATE
RYR1_HUMAN   809 PCHEAVLPRERLHLEPIKEYRREGPRGPHLVGPSRCLSHTDFVPCPVDTVQIVLPPHLER
RYR2_HUMAN   821 PCYEAVLPKEKLKVEHSREYKQERTYTRDLLGPTVSLTQAAFTPIPVDTSQIVLPPHLER
RYR3_HUMAN   808 PCYEALLPKEKMRLEPVKEYKRDADGIRDLLGTTQFLSQASFIPCPVDTSQVILPPHLEK

oldUNC-68    872 MHQKYAENLHELWAMRKIELGWSYGETRSETSRKHPCLTKFEYLPETEKKYNILLALTTM
UNC-68       872 MHQKYAENLHELWAMRKIELGWSYGETRSETSRKHPCLTKFEYLPETEKKYNILLALTTM
RYR1_HUMAN   869 IREKLAENIHELWALTRIEQGWTYGPVRDDNKRLHPCLVDFHSLPEPERNYNLQMSGETL
RYR2_HUMAN   881 IREKLAENIHELWVMNKIELGWQYGPVRDDNKRQHPCLVEFSKLPEQERNYNLQMSLETL
RYR3_HUMAN   868 IRDRLAENIHELWGMNKIELGWTFGKIRDDNKRQHPCLVEFSKLPETEKNYNLQMSTETL

oldUNC-68    932 KTIEALGYHLITEDPPCR--LRAVRLGPNFQQQNGYKPGPLDTHEIQLPAELQPLTEALA
UNC-68       932 KTIEALGYHLITEDPPCR--LRAVRLGPNFQQQNGYKPGPLDTHEIQLPAELQPLTEALA
RYR1_HUMAN   929 KTLLALGCHVGMADEKAEDNLKKTKLPKTYMMSNGYKPAPLDLSHVRLTPAQTTLVDRLA
RYR2_HUMAN   941 KTLLALGCHVGISDEHAEDKVKKMKLPKNYQLTSGYKPAPMDLSFIKLTPSQEAMVDKLA
RYR3_HUMAN   928 KTLLALGCHIAHVNPAAEEDLKKVKLPKNYMMSNGYKPAPLDLSDVKLLPPQEILVDKLA

oldUNC-68    990 RNTHNIWAKEKIKRGWTFGLSEHVDATQKRSPHLVPYEQVDERIKQANRESAAENIRALQ
UNC-68       990 RNTHNIWAKEKIKRGWTFGLSEHVDATQKRSPHLVPYEQVDERIKQANRESAAENIRALQ
RYR1_HUMAN   989 ENGHNVWARDRVGQGWSYSAVQDIPA--RRNPRLVPYRLLDEATKRSNRDSLCQAVRTLL
RYR2_HUMAN  1001 ENAHNVWARDRIRQGWTYGIQQDVKN--RRNPRLVPYTLLDDRTKKSNKDSLREAVRTLL
RYR3_HUMAN   988 ENAHNVWAKDRIKQGWTYGIQQDLKN--KRNPRLVPYALLDERTKKSNRDSLREAVRTFV

oldUNC-68   1050 LFGIFLEPPAHEHDEVAEKELRARKDNTRTYRAEATYAVCGGKWYFEFEILTAGYMKIGW
UNC-68      1050 LFGIFLEPPAHEHDEVAEKELRARKDNTRTYRAEATYAVCGGKWYFEFEILTAGYMKIGW
RYR1_HUMAN  1047 GYGYNIEPPDQ---EPSQVENQSRCDRVRIFRAEKSYTVQSGRWYFEFEAVTTGEMRVGW
RYR2_HUMAN  1059 GYGYNLEAPDQD-HAARAEVCSGTGERFRIFRAEKTYAVKAGRWYFEFETVTAGDMRVGW
RYR3_HUMAN  1046 GYGYNIEPSDQ--ELADSAVEKVSIDKIRFFRVERSYAVRSGKWYFEFEVVTGGDMRVGW

oldUNC-68   1110 MDIGSTPEIQLGADDRSYAFDGYLGRKWHQGAETYGKEWKIGDVVGCFLDLNDRTISFSL
UNC-68      1110 MDIGSTPEIQLGADDRSYAFDGYLGRKWHQGAETYGKEWKIGDVVGCFLDLNDRTISFSL
RYR1_HUMAN  1104 ARPELRPDVELGADELAYVFNGHRGQRWHLGSEPFGRPWQPGDVVGCMIDLTENTIIFTL
RYR2_HUMAN  1118 SRPGCQPDQELGSDERAFAFDGFKAQRWHQGNEHYGRSWQAGDVVGCMVDMNEHTMMFTL
RYR3_HUMAN  1104 ARPGCRPDVELGADDQAFVFEGNRGQRWHQGSGYFGRTWQPGDVVGCMINLDDASMIFTL
```

```
oldUNC-68   1170 NGELLLDPSGSEMAFDNVVCGDGLVPAMTLGSGQRGRLNFGQQSNSLKFFTTCGLQEGYE
UNC-68      1170 NGELLLDPSGSEMAFDNVVCGDGLVPAMTLGSGQRGRLNFGQQSNSLKFFTTCGLQEGYE
RYR1_HUMAN  1164 NGEVLMSDSGSETAFREIEIGDGFLPVCSLGPGQVGHLNLGQDVSSLRFFAICGLQEGFE
RYR2_HUMAN  1178 NGEILLDDSGSELAFKDFDVGDGFIPVCSLGVAQVGRMNFGKDVSTLKYFTICGLQEGYE
RYR3_HUMAN  1164 NGELLITNKGSELAFADYEIENGFVPICCLGLSQIGRMNLGTDASTFKFYTMCGLQEGFE

oldUNC-68   1230 PFCVNMYRTMPMWFAKQLPRFEDISTLKSGSILEVSRIPATGNSPPCLKILQKVTISEGG
UNC-68      1230 PFCVNMYRTMPMWFAKQLPRFEDISTLKSGSILEVSRIPATGNSPPCLKILQKVTISEGG
RYR1_HUMAN  1224 PFAINMQRPVTTWFSKGLPQFEPVP--LEHPHYEVSRVDGTVDTPPCLRLTHRTWGSQ--
RYR2_HUMAN  1238 PFAVNTNRDITMWLSKRLPQFLQVP--SNHEHIEVTRIDGTIDSSPCLKVTQKSFGSQ--
RYR3_HUMAN  1224 PFAVNMNRDVAMWFSKRLPTFVNVP--KDHPHIEVMRIDGTMDSPPCLKVTHKTFGTQ--

oldUNC-68   1290 PSEKAKMEYIKLSLPVKCNDTFVKNKDKETIRRQLQEYKPRSQSVVSQIRAPGIPKEFDD
UNC-68      1290 PSEKAKMEYIKLSLPVKCNDTFVKNKDKETIRRQLQEYKPRSQSVVSQIRAPGIPKEFDD
RYR1_HUMAN  1280 -NSLVEMLFLRLSLPVQFHQHFRCTAGATPLAPPGLQPP---------------------
RYR2_HUMAN  1294 -NSNTDIMFYRLSMPIECAEVFSKTVAGGLPGAGLFGPK---------------------
RYR3_HUMAN  1280 -NSNADMIYCRLSMPVECHSSFS-------------------------------------

oldUNC-68   1350 NKEKKGFLRSMLGSKDSHESDDDRRSRTNSKQPSVDGDEPPAVRRSLLELPHDERQIAED
UNC-68      1350 NKEKKGFLRSMLGSKDSHESDDDRRSRTNSKQPSVDGDEPPAVRRSLLELPHDERQIAED
RYR1_HUMAN  1318 ------------------------------------AEDEARAAEPDPDYENLRRSAGGW
RYR2_HUMAN  1332 ------------------------------------NDLEDYDADSDFEVL---------
RYR3_HUMAN  1302 ------------------------------------------------------------

oldUNC-68   1410 SMRDLNDRHSEKPKKGGLLSRLRDSSNTRKNFRDSDRRKEEKAAQLRQMKANSRSFDAGS
UNC-68      1410 SMRDLNDRHSEKPKKGGLLSRLRDSSNTRKNFRDSDRRKEEKAAQLRQMKANSRSFDAGS
RYR1_HUMAN  1342 SEAENGKEGTAKEGAPGGTPQAGGEAQPARAENEKDATTEKNKKRGFLFKAKKVAMMTQP
RYR2_HUMAN  1347 ----------MKTAHGHLVPDRVDKDKEATKPEFNNHKDYAQEKPSRLKQRFLLRRTKPD
RYR3_HUMAN  1302 ------------------------------------------------------------

oldUNC-68   1470 LDTSTLPTGQKDVLASSEMPLSGPGRQLTIKRSSIKKNKKGKKAEIALEKMEREKKGSII
UNC-68      1470 LDTSTLPTGQKDVLASSEMPLSGPGRQLTIKRSSIKKNKKGKKAEIALEKMEREKKGSII
RYR1_HUMAN  1402 PATPTLPRLPHDVVPA--------------------------------------------
RYR2_HUMAN  1397 YSTSHSARLTEDVLAD--------------------------------------------
RYR3_HUMAN  1302 ----HSPCLDSEAFQK--------------------------------------------

oldUNC-68   1530 PMDAQLDVLQEGDAHALVHHKDKVDEYYYGIRIFPGQDPSQVWVGWVTTQYHYYNVNFDG
UNC-68      1530 PMDAQLDVLQEGDAHALVHHKDKVDEYYYGIRIFPGQDPSQVWVGWVTTQYHYYNVNFDG
RYR1_HUMAN  1418 ------------DNRDDPEIILNTTTYYYSVRVFAGQEPSCVWAGWVTPDYHQHDMSFDL
RYR2_HUMAN  1413 ------------DRDDYDFLMQTST-YYYSVRIFPGQEPANVWVGWITSDFHQYDTGFDL
RYR3_HUMAN  1314 ------------RKQMQEILSHTTTQCYYAIRIFAGQDPSCVWVGWVTPDYHLYSEKFDL

oldUNC-68   1590 SQGVRKCRFSEADHHGTTVDSVQSQNCYMVNVSELLATTPDVANTKVSGTLIGCIIDTSI
UNC-68      1590 SQGVRKCRFSEADHHGTTVDSVQSQNCYMVNVSELLATTPDVANTKVSGTLIGCIIDTSI
RYR1_HUMAN  1466 SK-VRVVTVTMGDEQGNVHSSLKCSNCYMVWGGDFVSPGQQGRISHTD-LVIGCLVDLAT
RYR2_HUMAN  1460 DR-VRTVTVTLGDEKGKVHESIKRSNCYMVCAGESMSPGQ--GRNNNG-LEIGCVVDAAS
RYR3_HUMAN  1362 NK-NCTVTVTLGDERGRVHESVKRSNCYMVWGGDIVASSQRSNRSNVD-LEIGCLVDLAM

oldUNC-68   1650 GELSFQVGSTDTGIKFKLEPGAMLFPAAFVTPTATEILQFELGRIKYTFPLSAAMFKSCE
UNC-68      1650 GELSFQVGSTDTGIKFKLEPGAMLFPAAFVTPTATEILQFELGRIKYTFPLSAAMFKSCE
RYR1_HUMAN  1524 GLMTFTANGKESNTFFQVEPNTKLFPAVFVLPTHQNVIQFELGKQKNIMPLSAAMFQSER
RYR2_HUMAN  1516 GLLTFIANGKELSTYYQVEPSTKLFPAVFAQATSPNVFQFELGRIKNVMPLSAGLFKSEH
RYR3_HUMAN  1420 GMLSFSANGKELGTCYQVEPNTKVFPAVFLQPTSTSLFQFELGKLKNAMPLSAAIFRSEE

oldUNC-68   1710 KSLVPFCPPRLTVEQIESVYWARVPNETLRTTALKLSEVRGWSVLCNDPVRIMSVYIPEK
UNC-68      1710 KSLVPFCPPRLTVEQIESVYWARVPNETLRTTALKLSEVRGWSVLCNDPVRIMSVYIPEK
RYR1_HUMAN  1584 KNPAPQCPPRLEMQMLMPVSWSRMPNHFLQVETRRAGERLGWAVQCQEPLTMMALHIPEE
RYR2_HUMAN  1576 KNPVPQCPPRLHVQFLSHVLWSRMPNQFLKVDVSRISERQGWLVQCLDPLQFMSLHIPEE
RYR3_HUMAN  1480 KNPVPQCPPRLDVQTIQPVLWSRMPNSFLKVETERVSERHGWVVQCLEPLQMMALHIPEE
```

```
oldUNC-68  1770 DQSLDILEMIEMPDMLEFHRQTLNLYCKLASHGNHKVAHTLCQHIDEDQIMYAIKSHYLS
UNC-68     1770 DQSLDILEMIEMPDMLEFHRQTLNLYCKLASHGNHKVAHTLCQHIDEDQIMYAIKSHYLS
RYR1_HUMAN 1644 NRCMDILELSERLDLQRFHSHTLRLYRAVCALGNNRVAHALCSHVDQAQLLHALEDAHLP
RYR2_HUMAN 1636 NRSVDILELTEQEELLKFHYHTLRLYSAVCALGNHRVAHALCSHVDEPQLLYAIENKYMP
RYR3_HUMAN 1540 NRCVDILELCEQEDLMRFHYHTLRLYSAVCALGNSRVAYALCSHVDLSQLFYAIDNKYLP

oldUNC-68  1830 GPMRQGFHDLLIGLHLMSHTAARNSMAKEYVIPLVPQLQIKNVLD-------PDSESRYP
UNC-68     1830 GPMRQGFHDLLIGLHLMSHTAARNSMAKEYVIPLVPQLQIKNVLD-------PDSESRYP
RYR1_HUMAN 1704 GPLRAGYYDLLISIHLESACRSRRSMLSEYIVPLTPETRAITLFPPGRSTENGHPRHGLP
RYR2_HUMAN 1696 GLLRAGYYDLLIDIHLSSYATARLMMNNEYIVPMTEETKSITLFP------DENKKHGLP
RYR3_HUMAN 1600 GLLRSGFYDLLISIHLASAKERKLMMKNEYIIPITSTTRNIRLFP------DESKRHGLP

oldUNC-68  1883 QITGESVSMLSQMASEPVKKHVS----REDEMKLLPPSVDFEALKKHVMESLQSATHHAV
UNC-68     1883 QITGESVSMLSQMASEPVKKHVS----REDEMKLLPPSVDFEALKKHVMESLQSATHHAV
RYR1_HUMAN 1764 GVGVTTSLRPPHHFSPPCFVAALPAAGAAEAPARLSPAIPLEALRDKALRMLGEAVRDGG
RYR2_HUMAN 1750 GIGLSTSLRPRMQFSSPSFVSIS------NECYQYSPEFPLDILKSKTIQMLTEAVKEGS
RYR3_HUMAN 1654 GVGLRTCLKPGFRFSTPCFVVTG------EDHQKQSPEIPLESLRTKALSMLTEAVQCSG

oldUNC-68  1939 MNCRDLIGGDNTNHFEPLFKLFDQLLVIGLINDEELECLLRLIHPQAFDENYETGTTQK-
UNC-68     1939 MNCRDLIGGDNTNHFEPLFKLFDQLLVIGLINDEELECLLRLIHPQAFDENYETGTTQK-
RYR1_HUMAN 1824 QHARDPVGGSVEFQFVPVLKLVSTLLVMGIFGDEDVKQILKMIEPEVFTEEEEEEDEEEE
RYR2_HUMAN 1804 LHARDPVGGTTEFLFVPLIKLFYTLLIMGIFHNEDLKHILQLIEPSVFKEAATPEEESDT
RYR3_HUMAN 1708 AHIRDPVGGSVEFQFVPVLKLIGTLLVMGVFDDDDVRQILLLIDPSVFGEHSAGTEEGAE

oldUNC-68  1998 -----------------------------------------GLTQLELAEPVKIQLVSIL
UNC-68     1998 -----------------------------------------GLTQLELAEPVKIQLVSIL
RYR1_HUMAN 1884 GEEEDEEEKEEDEEETAQEKEDEEKEEEEAAEGEKEEGLEEGLLQMKLPESVKLQMCHLL
RYR2_HUMAN 1864 LEKELSVDDAKLQGAGEEEAKGGKRPKE-------------GLLQMKLPEPVKLQMCLLL
RYR3_HUMAN 1768 KEEVTQVEEKAVEAG---EKAGKEAPVK-------------GLLQTRLPESVKLQMCELL

oldUNC-68  2017 DHLCDIQLRYRIESLVAFTEGFVGELQSDQCKRY-MEIKQTDMPPAEAAKKTKEFRCPPK
UNC-68     2017 DHLCDIQLRYRIESLVAFTEGFVGELQSDQCKRY-MEIKQTDMPPAEAAKKTKEFRCPPK
RYR1_HUMAN 1944 EYFCDQELQHRVESIAAFAERYVDKLQANQRSRYGLLIKAFSMTAAETARRTREFRSPPQ
RYR2_HUMAN 1911 QYLCDCQVRHRIEAIVAFSDDFVAKLQDNQRFRYNEVMQALNMSAALTARKTKEFRSPPQ
RYR3_HUMAN 1812 SYLCDCELQHRVEAIVAFGDIYVSKLQANQKFRYNELMQALNMSAALTARKTKEFRSPPQ

oldUNC-68  2076 EQMFRLLMCKVKEERDPELMEEDADVDQCPMAEGLQQQLRDFCELLVGKIGNVKEGDSDD
UNC-68     2076 EQMFRLLMCKVKEERDPELMEEDADVDQCPMAEGLQQQLRDFCELLVGKIGNVKEGDSDD
RYR1_HUMAN 2004 EQINMLLQFKDG-----------TDEEDCPLPEEIRQDLLDFHQDLLAHCGIQLDGEEE-
RYR2_HUMAN 1971 EQINMLLNFKD-------------DKSECPCPEEIRDQLLDFHEDLMTHCGIELDEDGSL
RYR3_HUMAN 1872 EQINMLLNFQL-------------GEN-CPCPEEIREELYDFHEDLLLHCGVPLEEEE--

oldUNC-68  2136 QLALIESEEGSWVDSFARIV-VKVPPPVLEEGMEMQKKGTQNFREIIVTMLREWAQADFI
UNC-68     2136 QLALIESEEGSWVDSFARIV-VKVPPPVLEEGMEMQKKGTQNFREIIVTMLREWAQADFI
RYR1_HUMAN 2052 EPEEETTLGSRLMSLLEKVRLVKKKEEKPEEERSAEESKPRSLQELVSHMVVRWAQEDFV
RYR2_HUMAN 2018 DGNSDLTIRGRLLSLVEKVTYLKKKQA--EKPVESDSKKSSTLQQLISETMVRWAQESVI
RYR3_HUMAN 1916 EEEEDTSWTGKLCALVYKIKGPPKPEK--EQPTEEEERCPTTLKELISQTMICWAQEDQI

oldUNC-68  2195 ESKSLIRNMFRLLLRQYSGIREIRDAMSQTYVFHERNEKDVTDFLVYLIQIRELLTVQFE
UNC-68     2195 ESKSLIRNMFRLLLRQYSGIREIRDAMSQTYVFHERNEKDVTDFLVYLIQIRELLTVQFE
RYR1_HUMAN 2112 QSPELVRAMFSLLHRQYDGLGELLRALPRAYTISPSSVEDTMSLLECLGQIRSLLIVQMG
RYR2_HUMAN 2076 EDPELVRAMFVLLHRQYDGIGGLVRALPKTYTINGVSVEDTINLLASLGQIRSLLSVRMG
RYR3_HUMAN 1974 QDSELVRMMFNLLRRQYDSIGELLQALRKTYTISHTSVSDTINLLAALGQIRSLLSVRMG

oldUNC-68  2255 HTEEAILKRGLWKLMNNRIFFQHPDLMRLLSVHENVMSIMMNILTAQQGTVEHEGDELKE
UNC-68     2255 HTEEAILKRGLWKLMNNRIFFQHPDLMRLLSVHENVMSIMMNILTAQQGTVEHEGDELKE
RYR1_HUMAN 2172 PQEENLMIQSIGNIMNNKVFYQHPNLMRALGMHETVMEVMVNVLGGGESKEIR-------
RYR2_HUMAN 2136 KEEEKLMIRGLGDIMNNKVFYQHPNLMRALGMHETVMEVMVNVLGGGESKEIT-------
RYR3_HUMAN 2034 KEEELLMINGLGDIMNNKVFYQHPNLMRVLGMHETVMEVMVNVLG-TEKSQIA-------
```

```
oldUNC-68   2315 KAPIKDASEMVVACSRFLCYFCRTSRQNQKAMFEHLSFLLDNATMLLARPSLRGSVPLDV
UNC-68      2315 KAPIKDASEMVVACSRFLCYFCRTSRQNQKAMFEHLSFLLDNATMLLARPSLRGSVPLDV
RYR1_HUMAN  2225 ------FPKMVTSCCRFLCYFCRISRQNQRSMFDHLSYLLENSGIGLG---MQGSTPLDV
RYR2_HUMAN  2189 ------FPKMVANCCRFLCYFCRISRQNQKAMFDHLSYLLENSSVGLASPAMRGSTPLDV
RYR3_HUMAN  2086 ------FPKMVASCCRFLCYFCRISRQNQKAMFEHLSYLLENSSVGLASPSMRGSTPLDV

oldUNC-68   2375 AYSSFMDNNELALALKEEELDKVAVYLSRCGLQPNSELITKGYPDIGWDPVEGERYIDFL
UNC-68      2375 AYSSFMDNNELALALKEEELDKVAVYLSRCGLQPNSELITKGYPDIGWDPVEGERYIDFL
RYR1_HUMAN  2276 AAASVIDNNELALALQEQDLEKVVSYLAGCGLQSCPMLVAKGYPDIGWNPCGGERYLDFL
RYR2_HUMAN  2243 AAASVMDNNELALALREPDLEKVVRYLAGCGLQSCQMLVSKGYPDIGWNPVEGERYLDFL
RYR3_HUMAN  2140 AASSVMDNNELALSLEEPDLEKVVTYLAGCGLQSCPMLLAKGYPDVGWNPIEGERYLSFL

oldUNC-68   2435 RFCVWINGENVEENANLVIRLLIRRPECLGVALKGEG-QGLFSAFKEAIALSEDIRLLEN
UNC-68      2435 RFCVWINGENVEENANLVIRLLIRRPECLGVALKGEG-QGLFSAFKEAIALSEDIRLLEN
RYR1_HUMAN  2336 RFAVFVNGESVEENANVVVRLLIRKPECFGPALRGEGGSGLLAAIEEAIRISEDPARDGP
RYR2_HUMAN  2303 RFAVFCNGESVEENANVVVRLLIRRPECFGPALRGEGGNGLLAAMEEAIKIAEDPSRDGP
RYR3_HUMAN  2200 RFAVFVNSESVEENASVVVKLLIRRPECFGPALRGEGGNGLLAAMQGAIKISENPALDLP

oldUNC-68   2494 DSHPSLLSSGLLGENPTYPSKDAEGEDYIDLGAATLDFYSSLVDLLAKCAPDPMAIQAGK
UNC-68      2494 DSHPSLLSSGLLGENPTYPSKDAEGEDYIDLGAATLDFYSSLVDLLAKCAPDPMAIQAGK
RYR1_HUMAN  2396 GIRRDRRRE--------HFGEEPPEENRVHLGHAIMSFYAALIDLLGRCAPEMHLIQAGK
RYR2_HUMAN  2363 -SPNSGSSK--------TLDTEEEEDDTIHMGNAIMTFYSALIDLLGRCAPEMHLIHAGK
RYR3_HUMAN  2260 -SQGYKREV--------STGDDEEEEEIVHMGNAIMSFYSALIDLLGRCAPEMHLIQTGK

oldUNC-68   2554 GDSLRARAILRSLISLDDLGQILALRFTIPNLAAPSIEAVRRANAVQASHTQKAVMAHTF
UNC-68      2554 GDSLRARAILRSLISLDDLGQILALRFTIPNLAAP-------------------------
RYR1_HUMAN  2448 GEALRIRAILRSLVPLEDLVGIISLPLQIPTLGKDG------------------------
RYR2_HUMAN  2414 GEAIRIRSILRSLIPLGDLVGVISIAFQMPTIAKDG------------------------
RYR3_HUMAN  2311 GEAIRIRSILRSLVPTEDLVGIISIPLKLPSLNKDG------------------------

oldUNC-68   2614 AMVASLASEVSNTRRSQTFTSGTNLAKRGLPCVEEVDTDDSKSSDYASVHTSFSSGNELF
UNC-68      2589 ------------------------------------------------------------
RYR1_HUMAN  2484 ------------------------------------------------------------
RYR2_HUMAN  2450 ------------------------------------------------------------
RYR3_HUMAN  2347 ------------------------------------------------------------

oldUNC-68   2674 APKAEKEKVDVLKLNENKYPIIQRRRSSRFNPIDNTGPLPGLLPNHKGSVLLFLDRVYGI
UNC-68      2589 -------------------------------SIEDTGPLPGLLPNHKGSVLLFLDRVYGI
RYR1_HUMAN  2484 -------------------------------ALVQPKMSASFVPDHKASMVLFLDRVYGI
RYR2_HUMAN  2450 -------------------------------NVVEPDMSAGFCPDHKAAMVLFLDRVYGI
RYR3_HUMAN  2347 -------------------------------SVSEPDMAANFCPDHKAPMVLFLDRVYGI

oldUNC-68   2734 DQQDMLFHVLEQSFLPDLRAATMMDSPRALESDTALALNRYLCNSVLPLLTNHSHFFADA
UNC-68      2618 DQQDMLFHVLEQSFLPDLRAATMMDSPRALESDTALALNRYLCNSVLPLLTNHSHFFADA
RYR1_HUMAN  2513 ENQDFLLHVLDVGFLPDMRAAASLDTATFSTTEMALALNRYLCLAVLPLITKCAPLFAGT
RYR2_HUMAN  2479 EVQDFLLHLLEVGFLPDLRAAASLDTAALSATDMALALNRYLCTAVLPLLTRCAPLFAGT
RYR3_HUMAN  2376 KDQTFLLHLLEVGFLPDLRASASLDTVSLSTTEAALALNRYICSAVLPLLTRCAPLFAGT

oldUNC-68   2794 EHHSALLDATLHTVYRMNRLKSLTKNQRDAVSDFLVAITRELPPAMMIKLLKKVITDILT
UNC-68      2678 EHHSALLDATLHTVYRMNRLKSLTKNQRDAVSDFLVAITRELPPAMMIKLLKKVITDILT
RYR1_HUMAN  2573 EHRAIMVDSMLHTVYRLSRGRSLTKAQRDVIEDCLMSLCRYIRPSMLQHLLRRLVFDVPI
RYR2_HUMAN  2539 EHHASLIDSLLHTVYRLSKGCSLTKAQRDSIEVCLLSICGQLRPSMMQHLLRRLVFDVPL
RYR3_HUMAN  2436 EHCTSLIDSTLQTIYRLSKGRSLTKAQRDTIEECLLAICNHLRPSMLQQLLRRLVFDVPQ

oldUNC-68   2854 MNDMNVLVPLRLITLHYERCGKYYGSGN---HYGVASEQEKRLSMLLFDAIFDTLGSRPY
UNC-68      2738 MNDMNVLVPLRLITLHYERCGKYYGSGN---HYGVASEQEKRLSMLLFDAIFDTLGSRPY
RYR1_HUMAN  2633 LNEF-AKMPLKLLTNHYERCWKYYCLPTGWANFGVTSEEELHLTRKLFWGIFDSLAHKKY
RYR2_HUMAN  2599 LNEH-AKMPLKLLTNHYERCWKYYCLPGGWGNFGAASEEELHLSRKLFWGIFDALSQKKY
RYR3_HUMAN  2496 LNEY-CKMPLKLLTNHYEQCWKYYCLPSGWGSYGLAVEEELHLTEKLFWGIFDSLSHKKY
```

```
oldUNC-68   2911 DPELFGKALPCMTAIGSAISPDYTLTSGLEDVRNK-RREEEGAWIPRTVDVSRCEINRDL
UNC-68      2795 DPELFGKALPCMTAIGSAISPDYTLTSGLEDVRNK-RREEEGAWIPRTVDVSRCEINRDL
RYR1_HUMAN  2692 DPELYRMAMPCLCAIAGALPPDYVDASYSSKAEKKATVDAEGNFDPRPVETLNVIIPEKL
RYR2_HUMAN  2658 EQELFKLALPCLSAVAGALPPDYMESNYVSMMEKQSSMDSEGNFNPQPVDTSNITIPEKL
RYR3_HUMAN  2555 DPDLFRMALPCLSAIAGALPPDYLDTRITATLEKQISVDADGNFDPKPINTMNFSLPEKL

oldUNC-68   2970 EKMTELFAEHFHDSWASRKLEKGWVHGDLYSRANFTHPRLKPFALLKDFEKSFYKERCSE
UNC-68      2854 EKMTELFAEHFHDSWASRKLEKGWVHGDLYSRANFTHPRLKPFALLKDFEKSFYKERCSE
RYR1_HUMAN  2752 DSFINKFAEYTHEKWAFDKIQNNWSYGENIDEELKTHPMLRPYKTFSEKDKEIYRWPIKE
RYR2_HUMAN  2718 EYFINKYAEHSHDKWSMDKLANGWIYGEIYSDSSKVQPLMKPYKLLSEKEKEIYRWPIKE
RYR3_HUMAN  2615 EYIVTKYAEHSHDKWACDKSQSGWKYGISLDENVKTHPLIRPFKTLTEKEKEIYRWPARE

oldUNC-68   3030 CLKALMAWNYSFEMMDR-------DANDRASAARTLSGTSISNFAPKPIDLSSMTLEKDM
UNC-68      2914 CLKALMAWNYSFEMMDR-------DANDRASAARTLSGTSISNFAPKPIDLSSMTLEKDM
RYR1_HUMAN  2812 SLKAMIAWEWTIEKAREGEEEKTEKKKTRKISQSAQTYDPREGYNPQPPDLSAVTLSREL
RYR2_HUMAN  2778 SLKTMLAWGWRIERTREGDSMALYNRTRRISQTSQVSVDAAHGYSPRAIDMSNVTLSRDL
RYR3_HUMAN  2675 SLKTMLAVGWTVERTKEGEALVQQRENEKLRSVSQANQ--GNSYSPAPLDLSNVVLSREL

oldUNC-68   3083 VNAAEKMAEHSHLIWAKKVMNDLNTKGGFMPIPLVPWDLLTDFERRKDRFRASEILKFLQ
UNC-68      2967 VNAAEKMAEHSHLIWAKKVMNDLNTKGGFMPIPLVPWDLLTDFERRKDRFRASEILKFLQ
RYR1_HUMAN  2872 QAMAEQLAENYHNTWGRKKKQELEAKGGGTHPLLVPYDTLTAKEKARDREKAQELLKFLQ
RYR2_HUMAN  2838 HAMAEMMAENYHNIWAKKKKMELESKGGGNHPLLVPYDTLTAKEKAKDREKAQDILKFLQ
RYR3_HUMAN  2733 QGMVEVVAENYHNIWAKKKKLELESKGGGSHPLLVPYDTLTAKEKFKDREKAQDLFKFLQ

oldUNC-68   3143 YHGYHVNCPKDEQSQNDRLKSEGERTSVEKRFAYNLLEKLITYLEQASLKMKSVKPSQEL
UNC-68      3027 YHGYHVNCPKDEQSQNDRLKSEGERTSVEKRFAYNLLEKLITYLEQASLKMKSVKPSQEL
RYR1_HUMAN  2932 MNGYAVTRGLKDMELDS---------SSIEKRFAFGFLQQLLRWMDISQEFIAHLEAVVSS
RYR2_HUMAN  2898 INGYAVSRGFKDLELDT---------PSIEKRFAYSFLQQLIRYVDEAHQYILEFDG-GSR
RYR3_HUMAN  2793 VNGIIVSRGMKDMELDA---------SSMEKRFAYKFLKKILKYVDSAQEFIAHLEAIVSS

oldUNC-68   3203 TRRNSFKKEGQDVKFFEKVVLPLMHAYFNAHKNYFLEGSS--IVQTGTASNKEKEMVANL
UNC-68      3087 TRRNSFKKEGQDVKFFEKVVLPLMHAYFNAHKNYFLEGSS--IVQTGTASNKEKEMVANL
RYR1_HUMAN  2984 GRVEKSPHE-QEIKFFAKILLPLINQYFTNHCLYFLSTPAKVLGSGGHASNKEKEMITSL
RYR2_HUMAN  2949 GKGEHFPYE-QEIKFFAKVVLPLIDQYFKNHRLYFLSAASRPLCSGGHASNKEKEMVTSL
RYR3_HUMAN  2845 GKTEKSPRD-QEIKFFAKVLLPLVDQYFTSHCLYFLSSPLKPLSSSGYASHKEKEMVAGL

oldUNC-68   3261 FCRLAALLRIKNRAFGSVAKITVKCLQGLTQALDLRTLVKVNSDIVRTSLLTFFNNCADD
UNC-68      3145 FCRLAALLRIKNRAFGSVAKITVKCLQGLTQALDLRTLVKVNSDIVRTSLLTFFNNCADD
RYR1_HUMAN  3043 FCKLAALVRHRVSLFGTDAPAVVNCLHILARSLDARTVMKSGPEIVKAGLRSFFESASED
RYR2_HUMAN  3008 FCKLGVLVRHRISLFGNDATSIVNCLHILGQTLDARTVMKTGLESVKSALRAFLDNAAED
RYR3_HUMAN  2904 FCKLAALVRHRISLFGSDSTTMVSCLHILAQTLDTRTVMKSGSELVKAGLRAFFENAAED

oldUNC-68   3321 LYASVNELKKCIYYSEIEEYDDGGQYSLIRGQALKSWNSFEFANQMIVPVLTTMFAHLAR
UNC-68      3205 LYASVNELK------------DGGQYSLIRGQALKSWNSFEFANQMIVPVLTTMFAHLAR
RYR1_HUMAN  3103 IEKMVENLRL--------------GKVSQARTQVKGVGQNLTYTTVALLPVLTTLFQHIAQ
RYR2_HUMAN  3068 LEKTMENLKQ--------------GQFTHTRNQPKGVTQIINYTTVALLPMLSSLFEHIGQ
RYR3_HUMAN  2964 LEKTSENLKL--------------GKFTHSRTQIKGVSQNINYTTVALLPILTSIFEHVTQ

oldUNC-68   3381 NHFGTDLLLDDIQAACYKILDSLYMVTGLSSSISHRKSISGESEKHRPGLGQCLAAFASC
UNC-68      3253 NHFGTDLLLDDIQAACYKILDSLYMVTGLSSSISHRKSISGESEKHRPGLGQCLAAFASC
RYR1_HUMAN  3150 HQFGDDVILDDVQVSCYRTLCSIYSLG---------TTKNTYVEKLRPALGECLARLAAA
RYR2_HUMAN  3115 HQFGEDLILEDVQVSCYRILTSLYALG---------TSKSIYVERQRSALGECLAAFAGA
RYR3_HUMAN  3011 HQFGMDLLLGDVQISCYHILCSLYSLG---------TGKNIYVERQRPALGECLASLAAA

oldUNC-68   3441 FPVAFLEPEFNKSNKFSVLAKSQDQS---VQVQEMLQNLSTHIPHLEKLLTDLETVANNN
UNC-68      3313 FPVAFLEPEFNKSNKFSVLAKSQDQS---VQVQEMLQNLSTHIPHLEKLLTDLETVANNN
RYR1_HUMAN  3201 MPVAFLEPQLNEYNACSVYTTKSPRERAILGLPNSVEEMCPDIPVLERLMADIGGLAESG
RYR2_HUMAN  3166 FPVAFLETHLDKHNIYSIYNTKSSRERAALSLPTNVEDVCPNIPSLEKLMEEIVELAESG
RYR3_HUMAN  3062 IPVAFLEPTLNRYNPLSVFNTKTPRERSILGMPDTVEDMCPDIPQLEGLMKEINDLAESG
```

```
oldUNC-68   3498 TMYSDVPNVYDVDLPLMCSYMAHWFSVGPDGKRDKEDQRASVVQTTSVSCDHINRIFNAL
UNC-68      3370 TMYSDVPNVYDVDLPLMCSYMAHWFSVGPDGKRDKEDQRASVVQTTSVSCDHINRIFNAL
RYR1_HUMAN  3261 ARYTEMPHVIEITLPMLCSYLPRWWERGPEAPPS-ALPAGAPPPCTAVTSDHLNSLLGNI
RYR2_HUMAN  3226 IRYTQMPHVMEVILPMLCSYMSRWWEHGPEN-----NPERAEMCCTALNSEHMNTLLGNI
RYR3_HUMAN  3122 ARYTEMPHVIEVILPMLCNYLSYWWERGPEN-----LPPSTGPCCTKVTSEHLSLILGNI

oldUNC-68   3558 LKMIRNHVGIENAPWLCRVNFFAVQIIQNVTSDPVREFVLPIAERLRRMSEKAYKEEEHM
UNC-68      3430 LKMIRNHVGIENAPWLCRVNFFAVQIIQNVTSDPVREFVLPIAERLRRMSEKAYKEEEHM
RYR1_HUMAN  3320 LRIIVNNLGIDEASWMKRLAVFAQPIVSRARPELLQSHFIPTIGRLRKRAGKVVSEEEQL
RYR2_HUMAN  3281 LKIIYNNLGIDEGAWMKRLAVFSQPIINKVKPQLLKTHFLPLMEKLKKKAATVVSEEDHL
RYR3_HUMAN  3177 LKIINNNLGIDEASWMKRIAVYAQPIISKARPDLLRSHFIPTLEKLKKKAVKTVQEEEQL

oldUNC-68   3618 RTHP-DDADEG--TVAEDNARLVRDTYAFFPILMKYTDLHRAQWLKTPTWETDGVYENVA
UNC-68      3490 RTHP-DDADEG--TVAEDNARLVRDTYAFFPILMKYTDLHRAQWLKTPTWETDGVYENVA
RYR1_HUMAN  3380 RLEAKAEAQEGELLVRDEFSVLCRDLYALYPLLIRYVDNNRAQWLTEPNPSAEELFRMVG
RYR2_HUMAN  3341 KAEARGDMSEAELLILDEFTTLARDLYAFYPLLIRFVDYNRAKWLKEPNPEAEELFRMVA
RYR3_HUMAN  3237 KADGKGDTQEAELLILDEFAVLCRDLYAFYPMLIRYVDNNRSNWLKSPDADSDQLFRMVA

oldUNC-68   3675 VIFRIWSQSQHFKREELNYVAQFEEDAAATGGGDMKTGKAAIAERKKKRREGQDKMKEYF
UNC-68      3547 VIFRIWSQSQHFKREELNYVAQFEEDAAATGGGDMKTGKAAIAERKKKRREGQ-------
RYR1_HUMAN  3440 EIFIYWSKSHNFKREEQNFVVQNEINNMSFLTADNKSKMAKAGDIQSGGSDQERTKKKRR
RYR2_HUMAN  3401 EVFIYWSKSHNFKREEQNFVVQNEINNMSFLITDTKSKMSKAAVS-----DQERKKMKRK
RYR3_HUMAN  3297 EVFILWCKSHNFKREEQNFVIQNEINNLAFLTGDSKSKMSKAMQVKSGGQDQERKKTKRR

oldUNC-68   3735 DRNGRPIIKKDKHAASIVIACLKRLLPVGLNVFGGRELDIVQQSKEKFIQKETEEKIREF
UNC-68      3600 -------IKKDKHAASIVIACLKRLLPVGLNVFGGRELDIVQQSKEKFIQKETEEKIREF
RYR1_HUMAN  3500 G-------DRYSVQTSLIVATLKKMLPIGLNMCAPTDQDLITLAKTRYALKDTDEEVREF
RYR2_HUMAN  3456 G-------DRYSMQTSLIVAALKRLLPIGLNICAPGDQELIALAKNRFSLKDTEDEVRDI
RYR3_HUMAN  3357 G-------DLYSIQTSLIVAALKKMLPIGLNMCTPGDQELISLAKSRYSHRDTDEEVREH

oldUNC-68   3795 IKGLLEIPVKTDPTDKNAWQLSLYRKIGKSQMRGKDEMSQDAVIEKIFNMGQVSAILHTI
UNC-68      3653 IKGLLEIPVKTDPTDKNAWQLSLYRKIGKSQMRGKDEMSQDAVIEKIFNMGQVSAILHTT
RYR1_HUMAN  3553 LHNNLHLQGKVEGSPSLRWQMALYRGVPGR---EEDADDPEKIVRRVQEVSAVLYYLDQ-
RYR2_HUMAN  3509 IRSNIHLQGKLE-DPAIRWQMALYKDLPNR---TDDTSDPEKTVERVLDIANVLFHLEQK
RYR3_HUMAN  3410 LRNNLHLQEKSD-DPAVKWQLNLYKDVLK----SEEPFNPEKTVERVQRISAAVFHLEQ-

oldUNC-68   3855 TRTKDRRRRAHHRWKYNRKNINIAQVNHSDRNVVLSISCYVLSHLDGASANATVASMEKS
UNC-68      3713 EHP---------------------------------------------------------
RYR1_HUMAN  3609 ------------------------------------------------------------
RYR2_HUMAN  3565 SKRVGRRH----------------------------------------------------
RYR3_HUMAN  3464 ------------------------------------------------------------

oldUNC-68   3915 GLDATKTCRCGVFPHGSTVWNSKMTSRMEKEDIWKKVLTLQRKRMAISLITASHLYKTEL
UNC-68      3716 --------------------------QMQLSQAWKKVVSTQRKRAVVACFRMVPLYGIPR
RYR1_HUMAN  3609 ----------------------TEHPYKSKKAVWHKLLSKQRRRAVVACFRMTPLYNLPT
RYR2_HUMAN  3573 -------------------YCLVEHPQRSKKAVWHKLLSKQRKRAVVACFRMAPLYNLPR
RYR3_HUMAN  3464 ----------------------VEQPLRSKKAVWHKLLSKQRKRAVVACFRMAPLYNLPR

oldUNC-68   3975 HRGINFFLPAFSRLWME-EEDAGQDQLIADICSGVEEEEGPRIEIIMEEGVPIVASSEDT
UNC-68      3750 HRGINFFLPAFSRLWME-EEDAGQDQLIADICSGVEEEEGPRIEIIMEEGVPIVASSEDT
RYR1_HUMAN  3647 HRACNMFLESYKAAWILTEDHSFEDRMIDDLSKAGEQEE------------------EEE
RYR2_HUMAN  3614 HRAVNLFLQGYEKSWIETEEHYFEDKLIEDLAK-PGAEP------------------PEE
RYR3_HUMAN  3502 HRSINLFLHGYQRFWIETEEYSFEEKLVQDLAKSPKVEE------------------EEE

oldUNC-68   4034 KEKETNPDPLKQLIRCFQRAATSEETAASAIHEDSLYIRFADVMAKSIHIEEEDGEDG--
UNC-68      3809 KEKETNPDPLKQLIRCFQRAATSEETAASAIHEDSLYIRFADVMAKSIHIEEEDGEDG--
RYR1_HUMAN  3689 EVEEKKPDPLHQLVLHFSRTALTEK---SKLDEDYLYMAYADIMAKSCHLEEGGENGEAE
RYR2_HUMAN  3655 DEGTKRVDPLHQLILLFSRTALTEK---CKLEEDFLYMAYADIMAKSCHD-EEDDDG---
RYR3_HUMAN  3544 EETEKQPDPLHQIILYFSRNALTER---SKLEDDPLYTSYSSMMAKSCQSGEDEEED---
```

```
oldUNC-68   4092 EEGEIDQAAKEEQSQALRGEQAVLASRGAAIMCLMYLSASGGEPNEMVAQTLQLGIHLLS
UNC-68      3867 EEGEIDQAAKEEQSQALRGEQAVLASRGAAIMCLMYLSASGGEPNEMVAQTLQLGIHLLS
RYR1_HUMAN  3746 EEVEVSFEEKQMEKQRLLYQQARLHTRGAAEMVLQMISACKGETGAMVSSTLKLGISILN
RYR2_HUMAN  3708 EEEVKSFEEKEMEKQKLLYQQARLHDRGAAEMVLQTISASKGETGPMVAATLKLGIAILN
RYR3_HUMAN  3598 EDKEKTFEEKEMEKQKTLYQQARLHERGAAEMVLQMISASKGEMSPMVVETLKLGIAILN

oldUNC-68   4152 GGNVEIQKMLIEYLQLKKDVRFFTSMAGLMNKCSVLNLEMFERQIKVPTDTNVDAHGANE
UNC-68      3927 GGNVEIQKMLIEYLQLKKDVRFFTSMAGLMNKCSVLNLEMFERQIKA-------------
RYR1_HUMAN  3806 GGNAEVQQKMLDYLKDKKEVGFFQSIQALMQTCSVLDLNAFERQNKA-------------
RYR2_HUMAN  3768 GGNSTVQQKMLDYLKEKKDVGFFQSLAGLMQSCSVLDLNAFERQNKA-------------
RYR3_HUMAN  3658 GGNAGVQQKMLDYLKEKKDAGFFQSLSGLMQSCSVLDLNAFERQNKA-------------

oldUNC-68   4212 LLLRHLSSKIRHRSRQDRLGAQAEGLGMGAELAS-----GDNQNLNDADFTCSLFRFLQL
UNC-68      3974 -----------------------EGLGMGAELAS-----GDNQNLNDADFTCSLFRFLQL
RYR1_HUMAN  3853 -----------------------EGLGMVNEDGTVINRQNGEKVMADDEFTQDLFRFLQL
RYR2_HUMAN  3815 -----------------------EGLGMVTEEG------SGEKVLQDDEFTCDLFRFLQL
RYR3_HUMAN  3705 -----------------------EGLGMVTEEGTLIVRERGEKVLQNDEFTRDLFRFLQL

oldUNC-68   4267 TCEGHNLEFQNYLRTQPGHTTSVNLINCTVDYLLRLQESVMDFYWHYSSKEVIDEGGKEY
UNC-68      4006 TCEGHNLEFQNYLRTQPGHTTSVNLINCTVDYLLRLQESVMDFYWHYSSKEVIDEGGKEY
RYR1_HUMAN  3890 LCEGHNNDFQNYLRTQTGNTTTINIIICTVDYLLRLQESISDFYWYYSGKDVIEEQGKRN
RYR2_HUMAN  3846 LCEGHNSDFQNYLRTQTGNNTTVNIIISTVDYLLRVQESISDFYWYYSGKDVIDEQGQRN
RYR3_HUMAN  3742 LCEGHNSDFQNFLRTQMGNTTTVNVIISTVDYLLRLQESISDFYWYYSGKDIIDESGQHN

oldUNC-68   4327 FLRAIQVCSQVFNTLTESIQGPCVGNQMTLANSRLWDAINGFFFLFAHMMEKLYKNSTQL
UNC-68      4066 FLRAIQVCSQVFNTLTESIQGPCVGNQMTLANSRLWDAINGFFFLFAHMMEKLYKNSTQL
RYR1_HUMAN  3950 FSKAMSVAKQVFNSLTEYIQGPCTGNQQSLAHSRLWDAVVGFLHVFAHMMMKLAQDSSQI
RYR2_HUMAN  3906 FSKAIQVAKQVFNTLTEYIQGPCTGNQQSLAHSRLWDAVVGFLHVFAHMQMKLSQDSSQI
RYR3_HUMAN  3802 FSKALAVTKQIFNSLTEYIQGPCIGNQQSLAHSRLWDAVVGFLHVFANMQMKLSQDSSQI

oldUNC-68   4387 ELLREFLNLQKDMIVLMLSMLEGNVLNGSIGKQMVDALVESQPSVEKILKFSDMFLKLKD
UNC-68      4126 ELLREFLNLQKDMIVLMLSMLEGNVLNGSIGKQMVDALVESQPSVEKILKFSDMFLKLKD
RYR1_HUMAN  4010 ELLKELLDLQKDMVVMLLSLLEGNVVNGMIARQMVDMLVESSSNVEMILKFFDMFLKLKD
RYR2_HUMAN  3966 ELLKELMDLQKDMVVMLLSMLEGNVVNGTIGKQMVDMLVESSNNVEMILKFFDMFLKLKD
RYR3_HUMAN  3862 ELLKELLDLLQDMVVMLLSLLEGNVVNGTIGKQMVDTLVESSTNVEMILKFFDMFLKLKD

oldUNC-68   4447 LTTSQAFQDFDTNQDGWISPKEFQRAMESQKMYTVEDITYLMMCTDVNNDGKVDYMEFTE
UNC-68      4186 LTTSQAFQDFDTNQDGWISPKEFQRAMESQKMYTVEDITYLMMCTDVNNDGKVDYMEFTE
RYR1_HUMAN  4070 IVGSEAFQDYVTDPRGLISKKDFQKAMDSQKQFSGPEIQFLLSCSEADENEMINCEEFAN
RYR2_HUMAN  4026 LTSSDTFKEYDPDGKGVISKRDFHKAMESHKHYTQSETEFLLSCAETDENETLDYEEFVK
RYR3_HUMAN  3922 LTSSDTFKEYDPDGKGIISKKEFQKAMEGQKQYTQSEIDFLLSCAEADENDMFNYVDFVD

oldUNC-68   4507 RFHNPARDIGFNLAVLLVNLKEHITNDPRLEKIIEKAQTLLEYFDPFLGRIEIMGSSKRV
UNC-68      4246 RFHNPARDIGFNLAVLLVNLKEHITNDPRLEKIIEKAQTLLEYFDPFLGRIEIMGSSKRV
RYR1_HUMAN  4130 RFQEPARDIGFNVAVLLTNLSEHVPHDPRLHNFLELAESILEYFRPYLGRIEIMGASRRI
RYR2_HUMAN  4086 RFHEPAKDIGFNVAVLLTNLSEHMPNDTRLQTFLELAESVLNYFQPFLGRIEIMGSAKRI
RYR3_HUMAN  3982 RFHEPAKDIGFNVAVLLTNLSEHMPNDSRLKCLLDPAESVLNYFEPYLGRIEIMGGAKKI

oldUNC-68   4567 EKIYFEIQESWLEQWGKQQIRDSKNSFLFNVLQDDGGDQGKLEAFINFCEDTIFEMQHAA
UNC-68      4306 EKIYFEIQESWLEQWGKQQIRDSKNSFLFNVLQDDGGDQGKLEAFINFCEDTIFEMQHAA
RYR1_HUMAN  4190 ERIYFEISETNRAQWEMPQVKESKRQFIFDVVNE-GGEAEKMELFVSFCEDTIFEMQIAA
RYR2_HUMAN  4146 ERVYFEISESSRTQWEKPQVKESKRQFIFDVVNE-GGEKEKMELFVNFCEDTIFEMQLAA
RYR3_HUMAN  4042 ERVYFEISESSRTQWEKPQVKESKRQFIFDVVNE-GGEQEKMELFVNFCEDTIFEMQLAS

oldUNC-68   4627 AISSGDSDTKMERAIKQRDYFLQQTSADHISETFKSGYNYGISAASALSPQNISTTMRNV
UNC-68      4366 AISSGDSDTKMERAIKQRDYFLQQTSADHISETFKSGYNYGISAASALSPQNISTTMRNV
RYR1_HUMAN  4249 QISEPEGEPETDEDEGAGAAEAGAEGAEEGAAGLEGTAATAAAGATARVVAAAGRALRGL
RYR2_HUMAN  4205 QISESDLNERSANKEESEK-----ERPEEQGPRMAFFSILTVRSALFALRYNILTLMRML
RYR3_HUMAN  4101 QISESDSADRPEEEEEDEDSSYVLEIAGEEEEDGSLEPASAFAMACASVKRNVTDFLKRA
```

```
oldUNC-68   4687 SSS--------VRQMSWTQLLYAVIILFIRAGLAIGWAGYLLLMTIFRFGYFLTTSSEEE
UNC-68      4426 SSS--------VRQMSWTQLLYAVIILFIRAGLAIGWAGYLLLMTIFRFGYFLTTSSEEE
RYR1_HUMAN  4309 SYRSLRRRVRRLRRLTAREAATAVAALLWAAVTRAGAAGAGAAAGALGLLWGSLFGGGLV
RYR2_HUMAN  4260 SLKSLKKQMKKVKKMTVKDMVTAFFSSYWSIFMTLLHFVASVFRGFFRIICSLLLGGSLV
RYR3_HUMAN  4161 TLKNLRKQYRNVKKMTAKELVKVLFSFFWMLFVGLFQLLFTILGGIFQILWSTVFGGGLV

oldUNC-68   4739 EAARQEKEQAKMNNEHPSFNPPIIQEFHHSHVGVTAFGVGMNADHLNVN-----------
UNC-68      4478 EAARQEKEQAKMNNEHPSFNPPIIQEFHHSHVGVTAFGVGMNADHLNVN-----------
RYR1_HUMAN  4369 EGAKKVT-VTELLAGMPDPTSDEVHGEQPAGPGGDADGEGASEGAGDAAEGAGDEEEAVH
RYR2_HUMAN  4320 EGAKKIK-VAELLANMPDPTQDEVRGDGEEGER-KPLEAALPSEDLTDLKELTEESDLLS
RYR3_HUMAN  4221 EGAKNIR-VTKILGDMPDPTQFGIHDDTMEAERAEVMEPGITTELVHFIKGEKGDTDIMS

oldUNC-68   4788 ----------------------------SLPDFVPPERPETPETVLEEEKP--------L
UNC-68      4527 ----------------------------SLPDFVPPERPETPETVLEEEKP--------L
RYR1_HUMAN  4428 EAGPGGADGAVAVTDGGPFRPEGAGGLGDMGDTTPAEPPTPEGSPILKRKLGVDGVEEEL
RYR2_HUMAN  4378 DIF-----GLDLKREGGQYKLIPHNPNAGLSDLMSN--PVPMPEVQEKFQE-----QKAK
RYR3_HUMAN  4280 DLF-----GLHPKKEG-SLKHGPEVGLGDLSEIIGKDEPPTLESTVQKKRK-----AQAA

oldUNC-68   4812 NQETSPPTSPTSPASKAPSIYESIGAP---------------QMVQLQSEADFQQGQYEP
UNC-68      4551 NQETSPPTSPTSPASKAPSIYESIGAP---------------QMVQLQSEADFQQGQYEP
RYR1_HUMAN  4488 PPEPEPEPEPELEPEKADAENGEKEEVPEPTP---------EPPKKQAPPSPPPKKEEAG
RYR2_HUMAN  4426 EEEKEEKEETKSEPEKAEGEDGEKEEKAKEDK--------GKQKLRQLHTHRYGEPEVPE
RYR3_HUMAN  4329 EMKAANEAEGKVESEKADMEDGEKEDKDKEEEQAEYLWTEVTKKKKRRCGQKVEKPEAFT

oldUNC-68   4857 KIAESNSTKSRGSILNMLARNFKTIEKITLYLAFFINVILLFHRVDISH----------A
UNC-68      4596 KIAESNSTKSRGSILNMLARNFKTIEKITLYLAFFINVILLFHRVDISH----------A
RYR1_HUMAN  4539 GEFWGELEVQRVKFLNYLSRNFYTLRFLALFLAFAINFILLFYKVSDSPPGEDDMEGSAA
RYR2_HUMAN  4478 SAFWKKIIAYQQKLLNYFARNFYNMRMLALFVAFAINFILLFYKVSTSSVVEG------K
RYR3_HUMAN  4389 ANFFKGLEIYQTKLLHYLARNFYNLRFLALFVAFAINFILLFYKVTEEPLEEE------T

oldUNC-68   4907 ENAEAASEGDDDEDALESIFITG-----MQFPYVEYEITGWMLAQILYWISVLHLSTSFA
UNC-68      4646 ENAEAASEGDDDEDALESIFITG-----MQFPYVEYEITGWMLAQILYWISVLHLSTSFA
RYR1_HUMAN  4599 GDVSGAGSGGSSGWGLGAGEEAEGDEDENMVYYFLEESTGYMEP-ALRCLSLLHTLVAFL
RYR2_HUMAN  4532 ELPTRSSSENAKVTSLDSSSHRI-----IAVHYVLEESSGYMEP-TLRILAILHTVISFF
RYR3_HUMAN  4443 EDVANLWN------SFNDEEEEE-----AMVFFVLQESTGYMAP-TLRALAIIHTIISLV

oldUNC-68   4962 LLVSFYQLKIPLITFKREKEIARKLMFDGCWITEEDSEELGIVDTFMWYLDRIVVSAKSF
UNC-68      4701 LLVSFYQLKIPLITFKREKEIARKLMFDGCWITEEDSEELGIVDTFMWYLDRIVVSAKSF
RYR1_HUMAN  4658 CIIGYNCLKVPLVIFKREKELARKLEFDGLYITEQPED-----DDVKGQWDRLVLNTPSF
RYR2_HUMAN  4586 CIIGYYCLKVPLVIFKREKEVARKLEFDGLYITEQPSE-----DDIKGQWDRLVINTQSF
RYR3_HUMAN  4491 CVVGYYCLKVPLVVFKREKEIARKLEFDGLYITEQPSE-----DDIKGQWDRLVINTPSF

oldUNC-68   5022 PMMYWDKFVRRKTRSKFKDQVDEETLTSILGEEKMSTDS--------------------S
UNC-68      4761 PMMYWDKFVRRKTRSKFKDQVDEETLTSILGEEKMSTDS--------------------S
RYR1_HUMAN  4713 PSNYWDKFVKRKVLDKHGDIYGRERIAELLGMDLATLEITAHNERK-PNPPPGLLTWLMS
RYR2_HUMAN  4641 PNNYWDKFVKRKVMDKYGEFYGRDRISELLGMDKAALDFSDAREKKKPKKDSSLSAVLNS
RYR3_HUMAN  4546 PNNYWDKFVKRKVINKYGDLYGAERIAELLGLDKNALDFSPVEETK--AEAASLVSWLSS

oldUNC-68   5062 YDYRYSCWLWIGVILTNGQFLYRVGYLLCSACGVFLSPFFYAFHLIDVVLSFPMLKAILQ
UNC-68      4801 YDYRYSCWLWIGVILTNGQFLYRVGYLLCSACGVFLSPFFYAFHLIDVVLSFPMLKAILQ
RYR1_HUMAN  4772 IDVKYQIWK-FGVIFTDNSFLYLGWYMVMSLLGHYNN-FFFAAHLLDIAMGVKTLRTILS
RYR2_HUMAN  4701 IDVKYQMWK-LGVVFTDNSFLYLAWYMTMSVLGHYNN-FFFAAHLLDIAMGFKTLRTILS
RYR3_HUMAN  4604 IDMKYHIWK-LGVVFTDNSFLYLAWYTTMSVLGHYNN-FFFAAHLLDIAMGFKTLRTILS

oldUNC-68   5122 SVTHNLQQLILTIMMTLVVVYLYTVIAFNFFRKFYVQEGEEGEEPDRKCHNMLTCFIYHF
UNC-68      4861 SVTHNLQQLILTIMMTLVVVYLYTVIAFNFFRKFYVQEGEEGEEPDRKCHNMLTCFIYHF
RYR1_HUMAN  4830 SVTHNGKQLVMTVGLLAVVVYLYTVVAFNFFRKFYNK-SEDEDEPDMKCDDMMTCYLFHM
RYR2_HUMAN  4759 SVTHNGKQLVLTVGLLAVVVYLYTVVAFNFFRKFYNK-SEDGDTPDMKCDDMLTCYMFHM
RYR3_HUMAN  4662 SVTHNGKQLVLTVGLLAVVVYLYTVVAFNFFRKFYNK-SEDDDEPDMKCDDMMTCYLFHM
```

```
oldUNC-68   5182 YAGVRAGGGIGDELESPYGDDLEYPRMFYDISFFFFVIIILLAIMQGLIIDAFGELRDQQ
UNC-68      4921 YAGVRAGGGIGDELESPYGDDLEYPRMFYDISFFFFVIIILLAIMQGLIIDAFGELRDQQ
RYR1_HUMAN  4889 YVGVRAGGGIGDEIEDPAGDEYELYRVVFDITFFFFVIVILLAIIQGLIIDAFGELRDQQ
RYR2_HUMAN  4818 YVGVRAGGGIGDEIEDPAGDEYEIYRIIFDITFFFFVIVILLAIIQGLIIDAFGELRDQQ
RYR3_HUMAN  4721 YVGVRAGGGIGDEIEDPAGDPYEMYRIVFDITFFFFVIVILLAIIQGLIIDAFGELRDQQ

oldUNC-68   5242 ESATEKLESSCFICDIGKETFDRMPRGFEIHTTKEHNFANYLFFLQHLVNKDETEYTGQE
UNC-68      4981 ESATEKLESSCFICDIGKETFDRMPRGFEIHTTKEHNFANYLFFLQHLVNKDETEYTGQE
RYR1_HUMAN  4949 EQVKEDMETKCFICGIGSDYFDTTPHGFETHTLEEHNLANYMFFLMYLINKDETEHTGQE
RYR2_HUMAN  4878 EQVKEDMETKCFICGIGNDYFDTVPHGFETHTLQEHNLANYLFFLMYLINKDETEHTGQE
RYR3_HUMAN  4781 EQVREDMETKCFICGIGNDYFDTTPHGFETHTLQEHNLANYLFFLMYLINKDETEHTGQE

oldUNC-68   5302 TYVREKYDNRDWDFFPVGECFVKQYEDQL---
UNC-68      5041 TYVREKYDNRDWDFFPVGECFVKQYEDQLLQS
RYR1_HUMAN  5009 SYVWKMYQERCWDFFPAGDCFRKQYEDQLS--
RYR2_HUMAN  4938 SYVWKMYQERCWEFFPAGDCFRKQYEDQLN--
RYR3_HUMAN  4841 SYVWKMYQERCWDFFPAGDCFRKQYEDQLG—
```

**CLUSTALX2.1 Multiple Alignment;** representation by BoxShade (Fraction of sequence 0.5).
The denotation of the CRISPR-Cas9 mutations is based on an out-dated version of UNC-68 protein (oldUNC-68) while UNC-68 (NCBI Reference Sequence: NP_001343811.1) stands for an actual version of the protein. UniProtKB/Swiss-Prot: P21817 (hRyR1), Q92736 (hRyR2) and Q15413 (hRyR3).

CPVT-related point mutations tested in this manuscript are marked red and the area of the putative CASQ2 interaction site is labelled blue. Similarity is marked by shading.
