# Supplementary material for: An Optogenetic Arrhythmia Model—Insertion of Several Catecholaminergic Polymorphic Ventricular Tachycardia Mutations Into Caenorhabditis elegans UNC-68 Disturbs Calstabin-Mediated Stabilization of the Ryanodine Receptor Homolog
Source: Front Physiol. 2022 Mar 25;13:691829. doi: 10.3389/fphys.2022.691829 (PMC8990320; doi:10.3389/fphys.2022.691829)
Supplement: Supplementary file 5 [file Data_Sheet_3.PDF]

1 Supplementary Figures

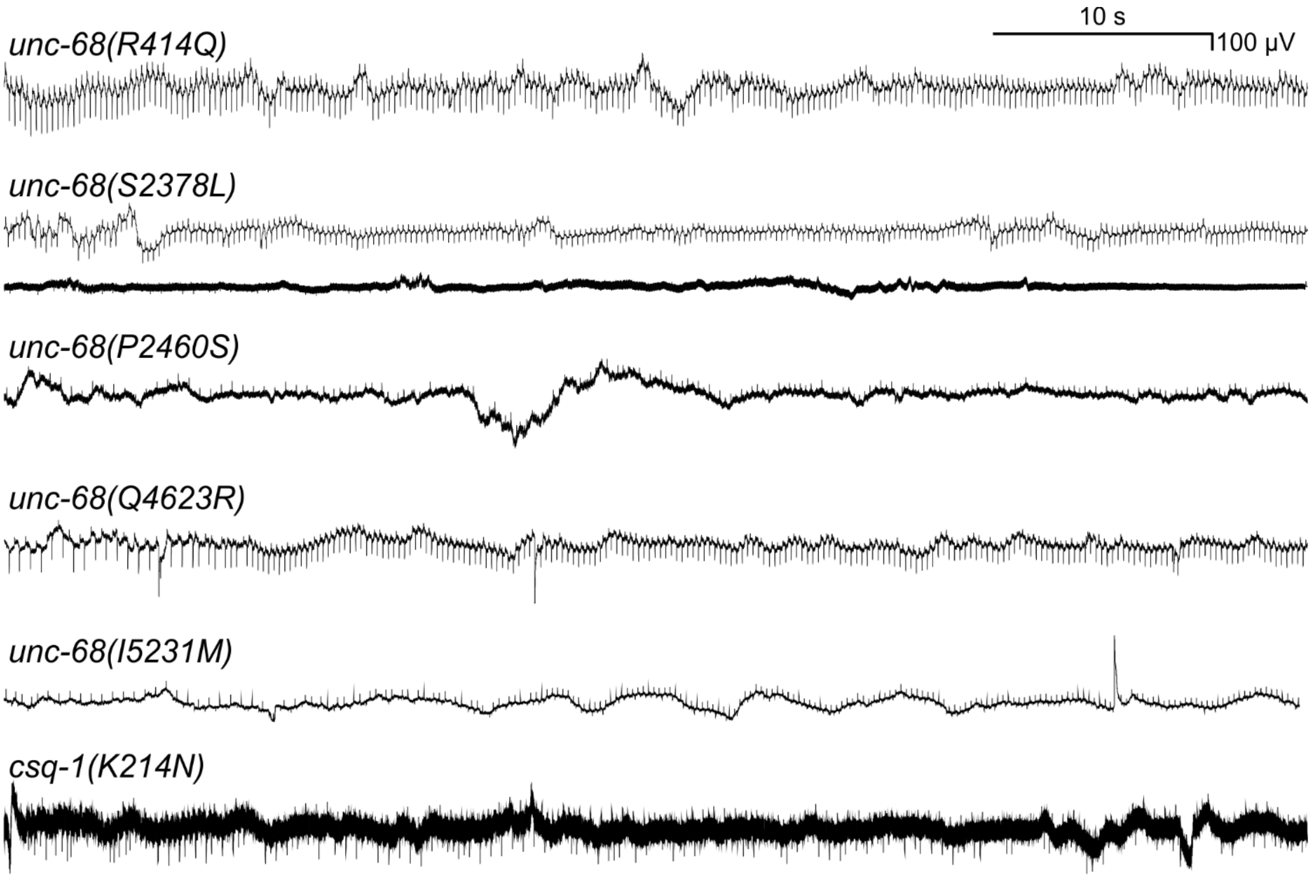

Figure S 1 Original recordings of EPGs recorded with the ScreenChip™ System. Displayed are exemplary raw data from NemAcquire plotted in OriginPro. Application of 4 Hz blue light pulses (1.5 mW/mm², 50 ms) for 60 s. For allele S2378L two examples are depicted because that allele showed strong variations between different recorded animals.

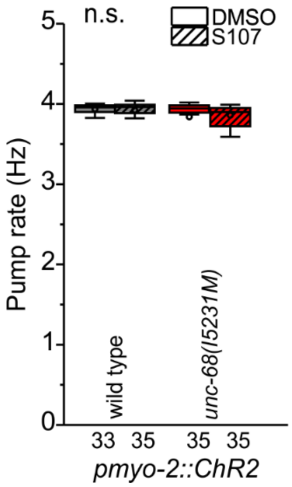

Figure S 2 Effect of the benzothiazepine S107 on *unc-68(I5231M)*. In ScreenChip™ recordings of EPGs under blue light stimulation (4 Hz, 50 ms) the effect of S107 (50 µM, 30 min pre-incubation, shaded boxes, as depicted) in comparison to the vehicle DMSO (0.1% in M9 buffer) on pump rate of UNC-68 mutants I5231M was determined. S107 has no effect since UNC-68 function is not affected by the mutation itself. Box plots include mean (open circle) and median (line). N-numbers are located either below the corresponding boxes. One-way ANOVA with Bonferroni post-hoc test was performed: n.s.=not significant.

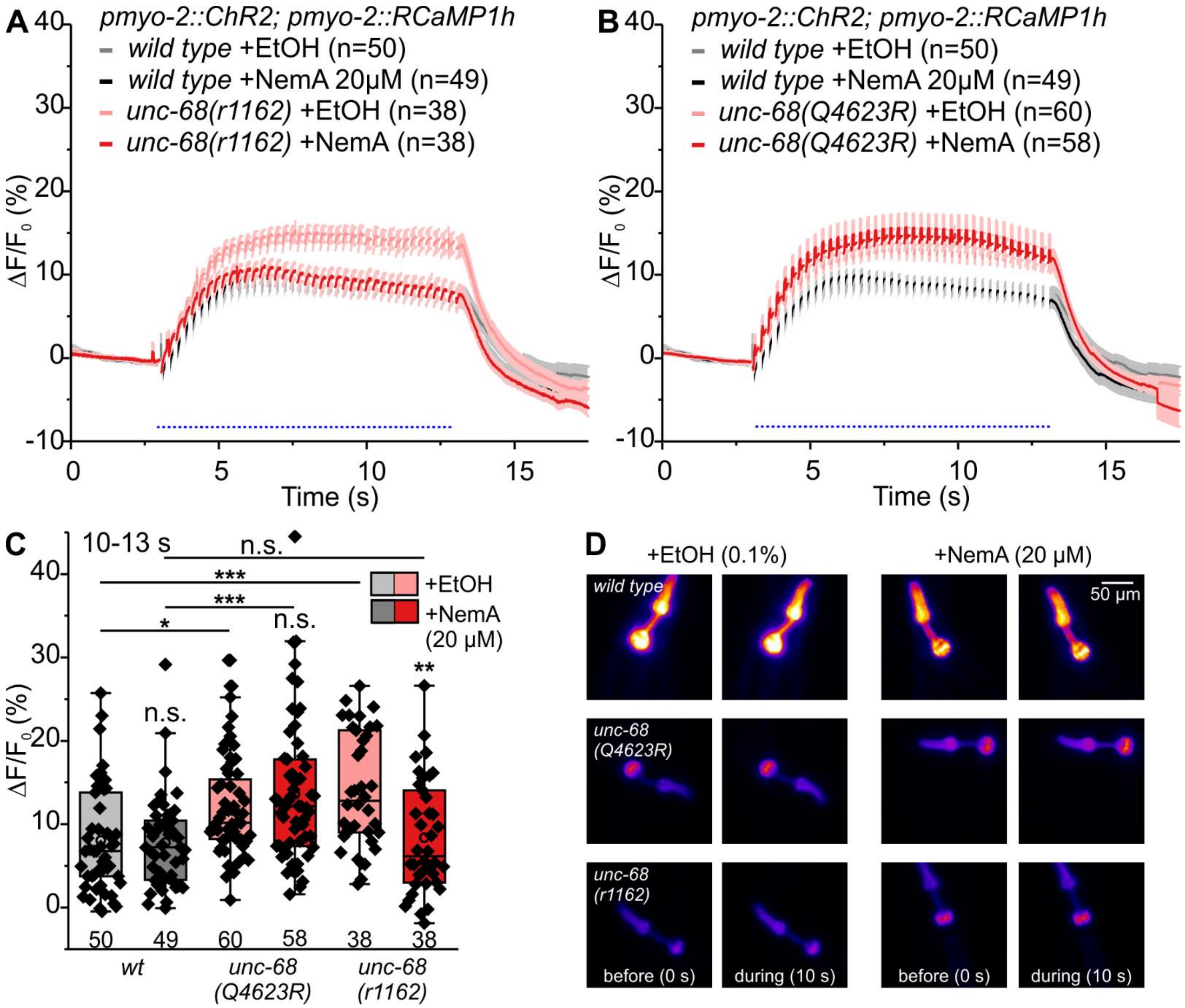

Figure S 3 Effect of Nemadipine-A (NemA) on *unc-68(r1162)* and *unc-68(Q4623R)* in RCaMP1h Ca^{2+} imaging. (A, B) Increase of $\Delta F/F_0$ during stimulation (4 Hz, 50 ms, 470 nm) and **(C)** amplitude of $\Delta F/F_0$ during plateau phase (time period: 10-13 s). **(A, C)** For the *unc-68* null allele (*r1162*) NemA (20 µM, 20 min), a specific inhibitor of the *C. elegans* voltage gated Ca^{2+} channel EGL-19, reversed the increase of $\Delta F/F_0$ (%) upon light stimulation in comparison to the vehicle ethanol (EtOH, 0.1% in M9 buffer). **(B, C)** While there was no effect on allele Q4623R. **(D)** Exemplary false-color representation of fluorescence intensity. **(A, B)** Depicted is the mean of $\Delta F/F_0$ (%) with SEM, time periods (~50 ms) including unspecific peaks induced by blue light stimulation are not displayed here for better outline. Box plots include every data point (rhomb), mean (open circle) and median (line). Significance levels (asterisks) indicating the comparison to the respective vehicle control are located above the corresponding NemA boxes, while the comparison to the respective wt animals is displayed above the connecting lines. N-numbers as indicated. One-way ANOVA with Bonferroni post-hoc test was performed for comparisons of the mean values during the plateau phase of the fluorescence rise (10-13 s): n.s.=not significant, $^{*}P \leq 0.05$, $^{**}P \leq 0.01$ and $^{***}P \leq 0.001$.

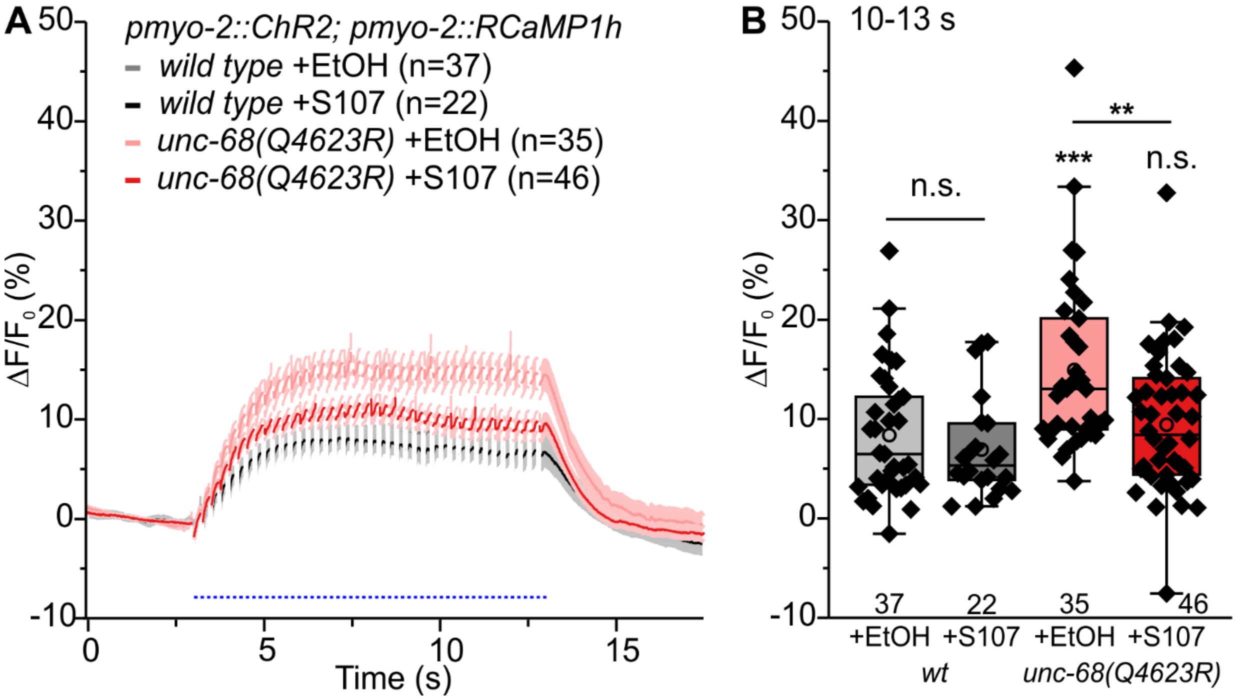

Figure S 4 Effect of the benzothiazepine S107 on *unc-68(Q4623R)* in RCaMP1h Ca^{2+} imaging. Increase of $\Delta F/F_0$ during stimulation (4 Hz, 50 ms, 470 nm) and **(B)** amplitude of $\Delta F/F_0$ during plateau phase (time period: 10-13 s). For allele Q4623R the increase of $\Delta F/F_0$ (%) upon light stimulation is reduced by S107 (50 µM, 30 min pre-incubation) in comparison to the vehicle ethanol (EtOH, 0.1% in M9 buffer). Depicted is the mean of $\Delta F/F_0$ (%) with SEM **(A)**, time periods (~50 ms) including unspecific peaks induced by blue light stimulation are not displayed here for better outline. Box plots include every data point (rhomb), mean (open circle) and median (line). Significance levels (asterisks) indicating the comparison to the respective wt control with or without S107 are located above the respective boxes, while the comparison between with and without S107 is displayed above the connecting lines. N-numbers as indicated. One-way ANOVA with Bonferroni post-hoc test was performed for comparisons of the mean values during the plateau phase of the fluorescence rise (10-13 s): n.s.=not significant, $*P \leq 0.05$, $**P \leq 0.01$ and $***P \leq 0.001$.

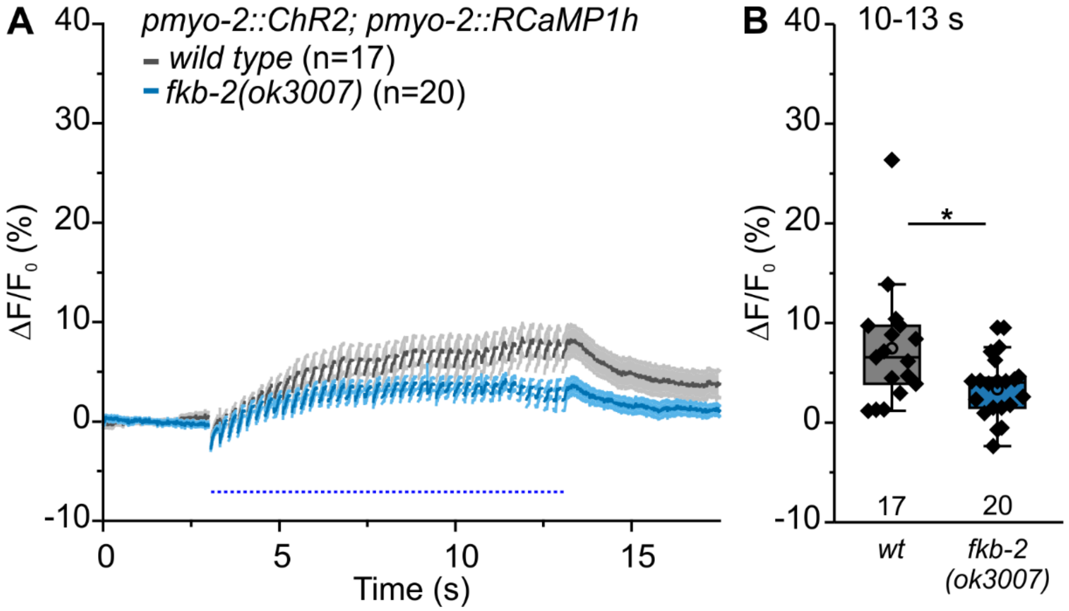

Figure S 5 RCaMP1h Ca^{2+} imaging in PMC of *fkb-2* deletion allele. (A) Increase of $\Delta F/F_0$ during optogenetic stimulation (4 Hz, 50 ms, 470 nm) and **(B)** amplitude of $\Delta F/F_0$ during plateau phase (time period: 10-13 s) of *fkb-2(ok3007)* deletion is reduced in comparison to wild type. Only worms that were able to follow 4 Hz stimulation have been included for analysis. Depicted is the mean of $\Delta F/F_0$ (%) with SEM, time periods (~50 ms) including unspecific peaks induced by blue light stimulation are not displayed here for better outline. Box plots include every data point (rhomb), mean (open circle) and median (line). N-numbers and significance level (asterisk) as indicated. One-way ANOVA with Bonferroni post-hoc test was performed for comparisons of the mean values during the plateau phase of the fluorescence rise (10-13 s): $*P \leq 0.05$.

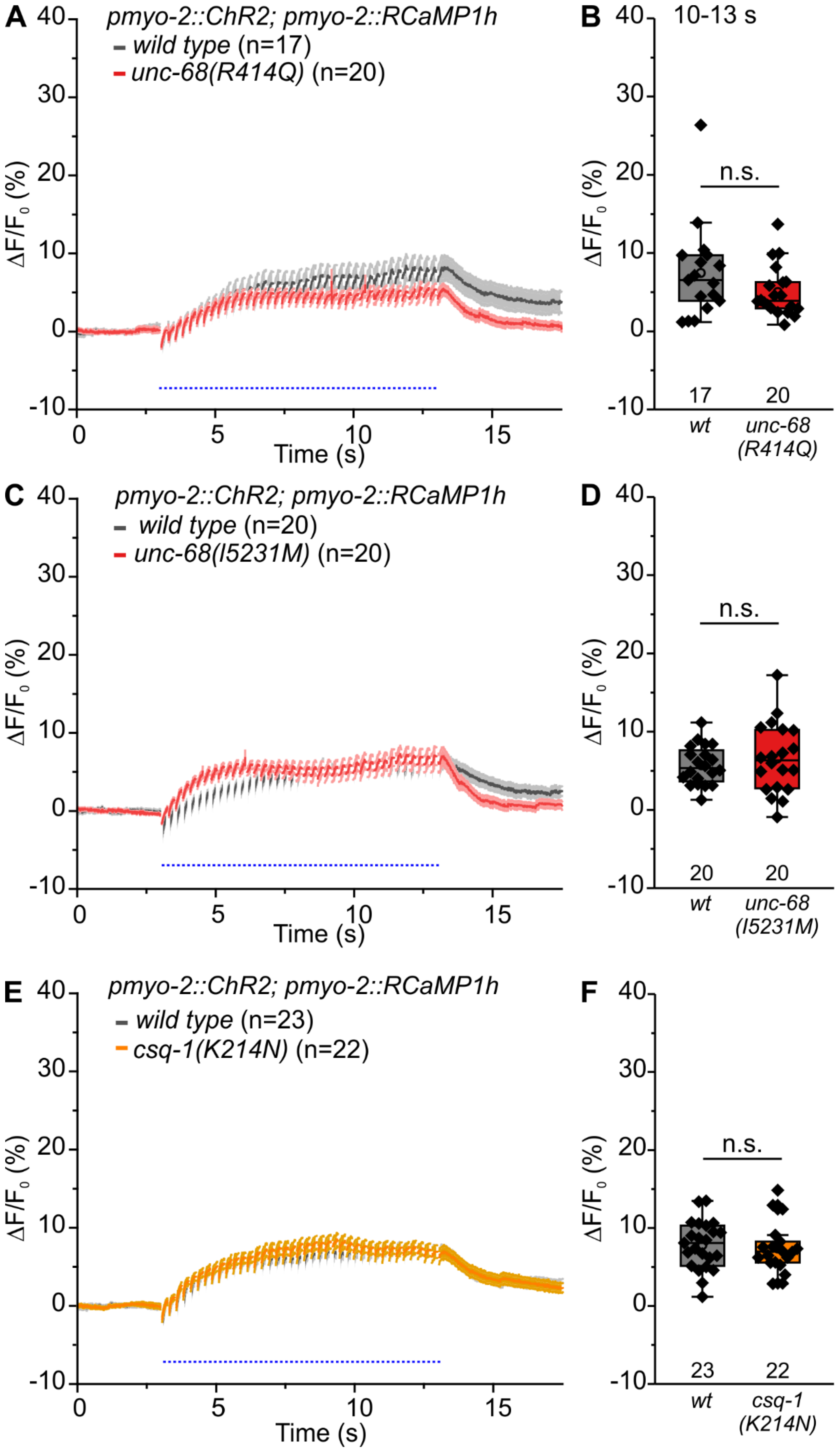
A
pmyo-2::ChR2; pmyo-2::RCaMP1h
wild type (n=17)
unc-68(R414Q) (n=20)
ΔF/F0 (%)
Time (s)
B
10-13 s
n.s.
17
20
wt
unc-68 (R414Q)
C
pmyo-2::ChR2; pmyo-2::RCaMP1h
wild type (n=20)
unc-68(I5231M) (n=20)
D
n.s.
20
20
wt
unc-68 (I5231M)
E
pmyo-2::ChR2; pmyo-2::RCaMP1h
wild type (n=23)
csq-1(K214N) (n=22)
F
n.s.
23
22
wt
csq-1 (K214N)

Figure S 6 RCaMP1h Ca^{2+} imaging in PMC of *unc-68* and *csq-1* alleles without an arrhythmic phenotype in the pharynx. Increase of $\Delta F/F_0$ during optogenetic stimulation (4 Hz, 50 ms, 470 nm) and amplitude of $\Delta F/F_0$ during plateau phase (time period: 10-13 s) of *unc-68(R414Q)* **(A, B)**, *unc-68(I5231M)* **(C, D)** and *csq-1(K214N)* **(E, F)**. Only worms that were able to follow 4 Hz stimulation have been included for analysis. Depicted is the mean of $\Delta F/F_0$ (%) with SEM, time periods (~50 ms) including unspecific peaks induced by blue light stimulation are not displayed here for better outline. Box plots include every data point (rhomb), mean (open circle) and median (line). N-numbers and significance levels (asterisks) as indicated. One-way ANOVA with Bonferroni post-hoc test was performed for comparisons of the mean values during the plateau phase of the fluorescence rise (10-13 s): n.s. = not significant.

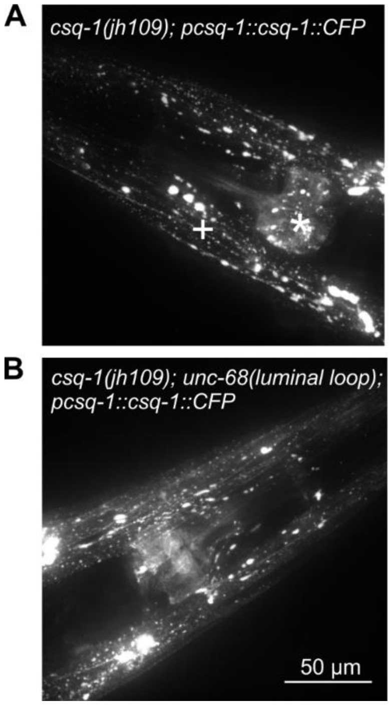

Figure S 7 Fluorescence micro-graphs of csq-1::CFP in csq-1 deletion background either with UNC-68 wt (A) or neutral luminal loop (B) display CSQ-1 localisation. A *csq-1(jh109)* allele carrying deletion strain, expressing an extrachromosomal CSQ-1::CFP rescue construct under the native CSQ-1 promotor, was crossed with the strain expressing the mutated luminal loop of *unc-68*. Fluorescent micrographs show no obviously different CSQ-1 distribution pattern between wt and neutral luminal loop bearing PMC (*) or BWM cells (+). Scale bar: 50 µm.

2 Supplementary Videos

Video S1 Spontaneous pumping on food of wt allele (ZX1662)

Video S2 Spontaneous pumping on food of allele S2378L

3 Supplementary Material and Methods

3.1 RCaMP1h Ca^{2+} imaging drug incubation

Young adult animals were placed onto a coverslip with a 5 µl drop of either S107 (50 µM) or the vehicle Ethanol (0.1% in M9 buffer) for 30 min pre-incubation. For Nemadipine-A (NemA, 20 µM) a 20 min pre-incubation was performed. Afterwards this coverslip was placed upside down onto 5 µl polystyrene beads on a10 % agarose pad (microscope slide).

3.2 Fluorescence microscopy

Young adult animals were placed onto 10 % agarose pads (M9 buffer) on microscope slides and immobilized with polysterene beads (0.1 µm diameter, at 2.5 % w/v, Sigma-Aldrich, USA). Images were acquired at an inverted confocal spinning disc microscope (Zeiss Axio Observer Z1; spinning disc unit CSU-X1A 5000 (D), Yokogawa, Japan), equipped with a 63x oil immersion objective (Zeiss C-Apochromat 63x/1.2 W autocorr M27), a laser diode, exciting at 445 nm for CFP excitation, a Cy3 filter set (AHF Analysentechnik, Germany) and an EMCCD camera (Zeiss EMCCD Kamera QImaging Rolera em-c2 (D)). Z-stacks were acquired Zen and merged with ImageJ (National Institutes of Health, USA; https://imagej.nih.gov/ij/index.html).

3.3 Supplementary strains:

ZX1520: *csq-1(jh109); zxEx746[pcsq-1(2,4 kb)::csq-1::CFP; pmyo-3::mCherry]*, **ZX2776:** *unc-68(zx10[I5231M]); zxIs20 [pmyo-2::ChR2(H134R)::mCherry; pges-1::nls::GFP]; zxIs124[pmyo-2::RCaMP35; pmyo-3::CFP]*, **ZX2915:** *pha-1(zx2); unc-68(zx5[R414Q]); zxIs20 [pmyo-2::ChR2(H134R)::mCherry; pges-1::nls::GFP]; zxIS124[pmyo-2::RCaMP35; pmyo-3::CFP]*, **ZX2916:** *fkb-2(ok3007) I.; zxIs20 [pmyo-2::ChR2(H134R)::mCherry; pges-1::nls::GFP]; zxIS124[pmyo-2::RCaMP35; pmyo-3::CFP]*, **ZX2917:** *csq-1(zx7[K214N]) ; zxIs20 [pmyo-2::ChR2(H134R)::mCherry; pges-1::nls::GFP]; zxIS124[pmyo-2::RCaMP35; pmyo-3::CFP]*, **ZX2781:** *csq-1(jh109); unc-68(zx9[neutral luminal loop]); zxEx746[pcsq-1(2,4 kb)::csq-1::CFP; pmyo-3::mCherry]*. ZX2781 was generated by crossing ZX2745 and ZX1520.
